# A Wittig-olefination–Claisen-rearrangement approach to the 3-methylquinoline-4-carbaldehyde synthesis

**DOI:** 10.3762/bjoc.8.197

**Published:** 2012-10-11

**Authors:** Mukund G Kulkarni, Mayur P Desai, Deekshaputra R Birhade, Yunus B Shaikh, Ajit N Dhatrak, Ramesh Gannimani

**Affiliations:** 1 Department of Chemistry, University of Pune, Ganeshkhind, Pune 411007, India; Fax: +91 (020) 25691728

**Keywords:** acetal, Claisen rearrangement, oxidative cleavage, ring-closure, Wittig olefination

## Abstract

Efficient syntheses are described for the synthetically important 3-methylquinoline-4-carbaldehydes **6a**–**h** from *o*-nitrobenzaldehydes **1a**–**h** employing a Wittig-olefination–Claisen-rearrangement protocol. The Wittig reaction of *o*-nitrobenzaldehydes with crotyloxymethylene triphenylphosphorane afforded crotyl vinyl ethers **2a**–**h**, which on heating under reflux in xylene underwent Claisen rearrangement to give 4-pentenals **3a**–**h**. Protection of the aldehyde group of the 4-pentenals as acetals **4a**–**h** and subsequent oxidative cleavage of the terminal olefin furnished nitroaldehydes **5a**–**h**. Reductive cyclization of these nitroaldehydes yielded the required 3-methylquinoline-4-carbaldehydes **6a**–**h** in excellent yields. Therefore, an efficient method was developed for the preparation of 3-methylquinoline-4-carbaldehydes from *o*-nitrobenzaldehydes in a simple five-step procedure.

## Introduction

Quinoline aldehydes are important synthetic intermediates in the synthesis of heterocyclic compounds that are used in the manufacturing of dyes [1] and pharmaceuticals [2–3]. 3-Substituted and 2,3-di-substituted quinoline-4-carbaldehyde derivatives are used in the synthesis of immunosuppressant KF20444 [4] and 5-HT_3_ receptor antagonists [5]. Quinoline mevalonolactones, prepared from 3-methylquinoline-4-carbaldehyde, act as inhibitors of HMG-CoA reductase [6]. 3-Substituted quinoline-4-carbaldehyde derivatives are used in the development of molecular probes for the identification of extra interaction sites in the midgorge and peripheral sites of butyrylcholinesterase (BuChE) [7]. These derivatives are also exploited in the synthesis of DNA binders [8], macrolides [9], antitumor agents [10] and for the treatment of viral and parasitic infections [11].

Though there are a number of quite efficient methods for the preparation of quinoline-4-carbaldehyde [12–24], only a few methods [6,9] are available for the preparation of 3-methylquinoline-4-carbaldehyde derivatives. In connection with the synthesis of camptothecin, we needed a general, high-yielding method for the synthesis of 3-methylquinoline-4-carbaldehyde. It was considered that a properly substituted 2-(2-nitrophenyl)pent-4-enal [25–27] could be a fitting intermediate for this purpose. Such an intermediate is easily accessible through the Wittig-olefination–Claisen-rearrangement protocol developed in our group [28–29].

## Results and Discussion

**Scheme 1:**
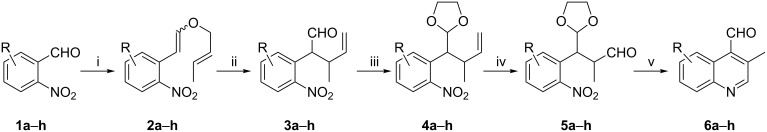
Reagents and conditions: (i) Ph_3_P^+^CH_2_OCH_2_CH=CHCH_3_Cl^−^, *t-*BuOK, dry THF, 0 °C; (ii) xylene, reflux, 5–7 h; (iii) 3 equiv ethylene glycol, cat. *p*-TSA, toluene, reflux, 3–4 h; (iv) cat. potassium osmate, 2 equiv NMO, 2 equiv NaIO_4_, aq THF, 3–4 h; (v) 5 equiv Zn, AcOH, reflux, 0.5 h.

Reaction of the *o*-nitrobenzaldehydes **1a**–**h** with crotyloxymethylene triphenylphosphorane under optimized reaction conditions (Scheme 1) gave crotyl vinyl ethers in good yields (Table 1). The geometrical isomers of the crotyl vinyl ethers **2a**–**d** were well separated on TLC, and it was possible to separate them by column chromatography. In the case of other crotyl vinyl ethers **2e**–**h**, all attempts to separate these (*E*)*-* or (*Z*)*-*isomers were unsuccessful.

**Table 1:** Synthesis of 3-methylquinoline-4-carbaldehydes.

Aldehydes **1**	% Yields of				
	**2**^a^	**3**^b^	**4**^b^	**5**^b^	**6**

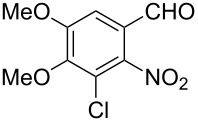 **1a**	90 (*Z*/*E* = 1:1.48)	85 (dr = 1:1.21)	92 (dr = 1:1.5)	91 (dr = 1:2.46)	85
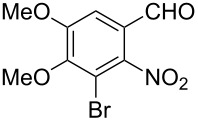 **1b**	91 (*Z*/*E* = 1:1.42)	83 (dr = 1:1.36)	91 (dr = 1:1.12)	90 (dr = 1:1.03)	84
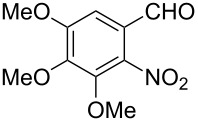 **1c**	92 (*Z*/*E* = 1:1.87)	84 (dr = 1:1.74)	90 (dr = 1:1.24)	91 (dr = 1:1.52)	85
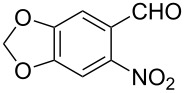 **1d**	93 (*Z*/*E* = 1:1.93)	86 (dr = 1:1.63)	92 (dr = 1:1.66)	91 (dr = 1:1.41)	83
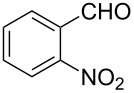 **1e**	91^c^	85 (dr = 1:1.16)	91 (dr = 1:2.63)	90 (dr = 1:6)	80^d^
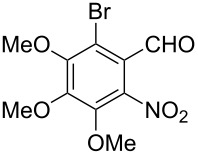 **1f**	90 (only (*E*)-isomer)	83 (dr = 1:9.01)	92 (dr = 1:1)	91 (dr = 1:6.58)	79
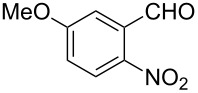 **1g**	92 (*Z*/*E* = 1:2.06)	83 (dr = 1:2.42)	91 (dr = 1:1.11)	90 (dr = 1:1.5)	80
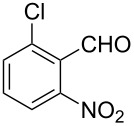 **1h**	91 (*Z*/*E* = 1:1.94)	85 (dr = 1:1.87)	90 (dr = 1:1.86)	91 (dr = 1:1.44)	82

^a^**2a**–**d** are separable geometrical isomers, whereas **2e**, **2g** and **2h** are mixtures of inseparable geometrical isomers. The *Z*/*E* ratio of the geometrical isomers of **2g**–**h** was calculated from their NMR signals. ^b^**3a**–**h**, **4a**–**h** and **5a**–**h** are mixtures of diastereomers, and the diastereomeric ratio (dr) was calculated from their NMR signals. ^c^The *Z*/*E* ratio of the geometrical isomers of **2e** cannot be calculated from their NMR signals. ^d^Known compound [6,9].

Claisen rearrangement on either (*E*)- or (*Z*)-isomers **2a**–**d** also led to a diastereomeric mixture of 4-pentenals. However, these diastereomers remained inseparable. The crotyl vinyl ethers **2e**–**h**, on heating under reflux in anhydrous xylene, underwent the Claisen rearrangement smoothly to give the diastereomeric mixture of the corresponding 4-pentenals **3e**–**h** in good yields (Table 1).

Treatment of the 4-pentenals **3a**–**h** with ethylene glycol furnished the corresponding acetals **4a**–**h** in good yields (Table 1). From the NMR spectra of these acetals, it was clear that they were also a mixture of diastereomers, although they appeared to be homogeneous on TLC. All attempts to separate the diastereomers at this stage were also unsuccessful. Subjecting these acetals to oxidative cleavage in aq THF furnished the aldehydes **5a**–**h** in good yields (Table 1). The NMR of these aldehydes revealed them again to be a mixture of diastereomers, although they appeared to be homogeneous on TLC. Reductive cyclization of these nitroaldehydes furnished the required 3-methylquinoline-4-carbaldehydes **6a**–**h**.

## Conclusion

A new and efficient methodology for the construction of a 3-methylquinoline-4-carbaldehyde framework, with 50–55% overall yield, through a Wittig-olefination–Claisen-rearrangement protocol has been developed.

## Experimental

### General

Silica gel (100–200 mesh) was used for column chromatography. IR spectra were recorded on a Perkin Elmer model 1600 series FTIR instrument. ^1^H and ^13^C NMR (ppm, TMS, internal standard) in CDCl_3_ were recorded on a JEOL FX 90Q, Varian Mercury 300 MHz and 75 MHz, respectively. CHN analysis was performed on a Thermo FLASH EA model 1112 series. TLC was checked either under UV light and/or charring after dipping into anisaldehyde solution.

#### General procedure for the Wittig olefination

To a suspension of the *o*-nitrobenzaldehyde (20 mmol) and crotyloxymethylenetriphenylphosphonium chloride (24 mmol, 1.2 equiv) in dry THF (40 mL) at 0 °C was added *t*-BuOK (24 mmol, 1.2 equiv) in small portions. After 40–45 min (TLC, ethyl acetate/petroleum ether 1:9), THF was removed under vacuum. Water (25 mL) was added to the reaction mixture, and then the aqueous layer was extracted with ethyl acetate (3 × 15 mL), the combined organic layer was dried over sodium sulfate, and ethyl acetate was evaporated under vacuum. The crude product, i.e., crotyl vinyl ether, was purified by using silica-gel column chromatography (mobile phase 1–3% ethyl acetate in petroleum ether). Crotyl vinyl ethers (**a**–**h** were obtained in 84–89% yield.

#### General procedure for the Claisen rearrangement

The crotyl vinyl ethers **2a**–**h** (17 mmol) obtained from the Wittig reaction were dissolved in anhydrous xylene (35 mL) and the solution was heated under reflux for 5–7 h (TLC, ethyl acetate/petroleum ether 1:9). Then, the solvent was removed under reduced pressure. The crude aldehyde was purified by using silica-gel column chromatography (mobile phase 2–5% ethyl acetate in pet. ether). 4-Pentenals **3a**–**h** were obtained in 83–89% yield.

#### General procedure for the protection of aldehyde

Aldehydes **3a**–**h** obtained from Claisen rearrangement (15 mmol) were dissolved in anhydrous toluene (25 mL). To this solution, a catalytic amount of *p*-TSA (1.5 mmol, 0.1 equiv) and ethylene glycol (45 mmol, 3 equiv) were added. The reaction mixture was heated under reflux for 3–4 h by using a Dean–Stark condenser (TLC, ethyl acetate/petroleum ether 1:9). After removal of the solvent under reduced pressure, water (20 mL) was added to the reaction mixture, and then the aqueous layer was extracted with ethyl acetate (3 × 15 mL), the combined organic layer was dried over sodium sulfate, and ethyl acetate was evaporated under vacuum. Finally, the product was purified by silica-gel column chromatography (mobile phase 1–3% ethyl acetate in petroleum ether). The products **4a**–**h** were obtained in 89–93% yield.

#### General procedure for the oxidative cleavage of alkene

Alkenes **4a**–**h** (13.5 mmol), obtained as described above, were dissolved in aq. THF (30 mL, THF/H_2_O 1:1). *N*-Methylmorpholine-*N*-oxide (NMO) (27 mmol, 2 equiv) and potassium osmate (0.027 mmol, 2 mol %) were added to this solution. The mixture was stirred at room temperature for 2–3 h until the starting compound disappeared (TLC, ethyl acetate/petroleum ether 1:9). Then, sodium metaperiodate was added (27 mmol, 2 equiv) and stirring was continued for 1 h (TLC, ethyl acetate/petroleum ether 1:9). THF was removed under reduced pressure. Water (20 mL) was added to the reaction mixture, and then the aqueous layer was extracted with ethyl acetate (3 × 10 mL), the combined organic layer was dried over sodium sulfate, and ethyl acetate was evaporated under vacuum. The crude product was obtained after removal of the solvent under reduced pressure. The product was purified by using silica-gel column chromatography (mobile phase 4–7% ethyl acetate in petroleum ether). The products **5a**–**h** were obtained in 89–95% yield.

#### General procedure for the reductive cyclization

Aldehydes **5a**–**h** (11 mmol) were dissolved in glacial acetic acid (20 mL) and heated under reflux with zinc dust (5 equiv) for 0.5 h (TLC, ethyl acetate/petroleum ether 1:9). Acetic acid was evaporated under vacuum, and chloroform was added to the residue. The solution was filtered through a celite bed. CHCl_3_ was removed under reduced pressure. Water (25 mL) was added to the reaction mixture, and then the aqueous layer was extracted with ethyl acetate (3 × 15 mL), the combined organic layer was dried over sodium sulfate, and ethyl acetate was evaporated under vacuum. The crude product was obtained and purified by silica-gel column chromatography (mobile phase 2–3% ethyl acetate in petroleum ether). The products **6a**–**h** were obtained in 84–87% yield.

## Supporting Information

File 1 IR, ^1^H NMR, ^13^C NMR and CHN analysis and spectral data of synthesized compounds.
The geometric isomeric ratios for **2g** and **2h** and diastereomeric ratios for **3a**–**h**, **4a**–**h** and **5a**–**h** were calculated from their NMR signals.
