# Supplementary material for: A Wittig-olefination–Claisen-rearrangement approach to the 3-methylquinoline-4-carbaldehyde synthesis
Source: Beilstein J Org Chem. 2012 Oct 11;8:1725–9. doi: 10.3762/bjoc.8.197 (PMC3511006; doi:10.3762/bjoc.8.197)
Supplement: File 1 — IR, 1H NMR, 13C NMR and CHN analysis and spectral data of synthesized compounds. The geometric isomeric ratios for 2g and 2h and diastereomeric ratios for 3a–h, 4a–h and 5a–h were calculated from their NMR signals. [file Beilstein_J_Org_Chem-08-1725-s001.pdf]

Supporting Information

for

A Wittig-olefination–Claisen-rearrangement approach to the 3-methylquinoline-4-carbaldehyde synthesis

Mukund G. Kulkarni*, Mayur P. Desai, Deekshaputra R. Birhade, Yunus B. Shaikh, Ajit N. Dhatrak and Ramesh Gannimani

Address: Department of Chemistry, University of Pune, Ganeshkhind, Pune 411007, India; Fax: +91 (020) 25691728

Email: Mukund G. Kulkarni* - mgkulkarni@chem.unipune.ac.in

* Corresponding author

IR, ^{1}H NMR, ^{13}C NMR and CHN analysis and spectral data of synthesized compounds

General: Silica gel (100–200 mesh) was used for column chromatography. IR spectra were recorded on a Perkin Elmer model 1600 series FTIR instrument. ^{1}H and ^{13}C NMR (ppm, TMS, internal standard) in $CDCl_3$ were recorded on JEOL FX 90Q, Varian Mercury 300 MHz and 75 MHz, respectively. CHN analysis was performed on a Thermo FLASH EA model 1112 series.

Data for compound 2a. Thick viscous liquid (90%), ((*Z*:*E*) = 1:1.48)

1-((*Z*)-2-((*E*)-But-2-en-1-yloxy)vinyl)-3-chloro-4,5-dimethoxy-2-nitrobenzene ((*Z*,*E*)-2a).

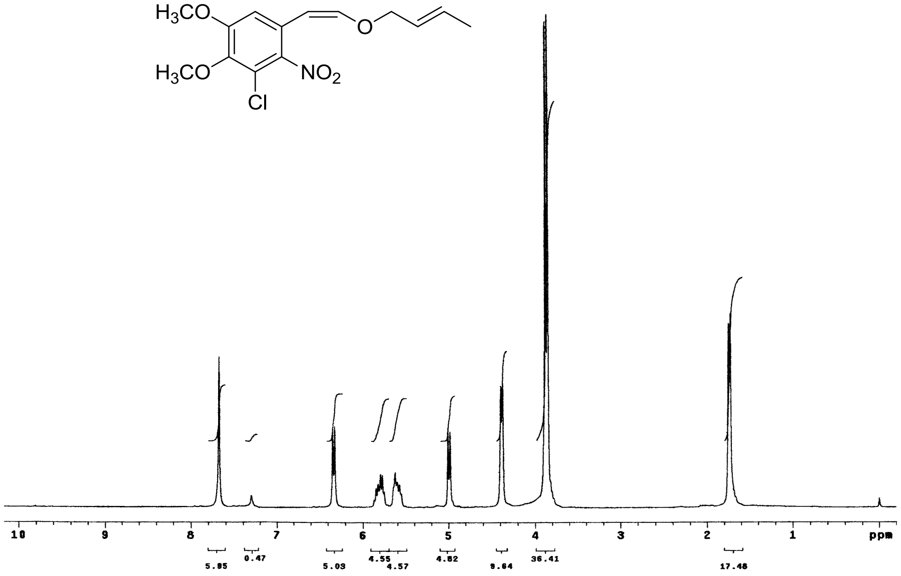

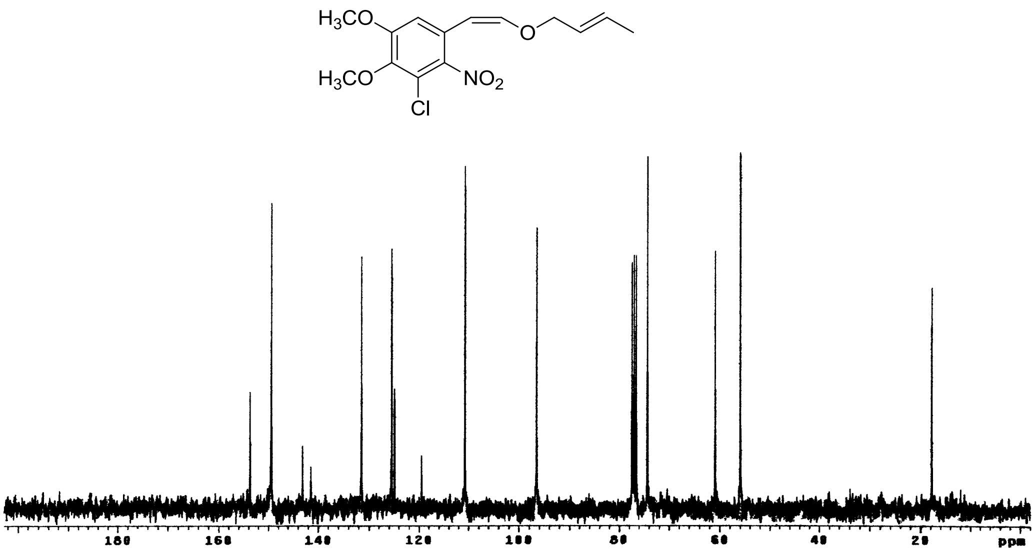

IR ($CHCl_3$) ν/cm^{-1} 2935.7, 1645.3, 1535.3, 1365.6, 1215.1, 1111.0; ^{1}H NMR ($CDCl_3$, 300 MHz, δ/ppm) 1.74 (d, *J* = 6.0 Hz, 3H, **H**$_3$C-CH=), 3.86 (s, 3H, **H**$_3$CO-Ar), 3.88 (s, 3H, **H**$_3$CO-Ar), 4.38

(d, J = 5.7 Hz, 2H, -C**H**$_2$-O-), 4.99 (d, J = 7.1 Hz, 1H, =C**H**-Ar), 5.56-5.63 (m, 1H, =C**H**-CH$_3$), 5.75-5.87 (m, 1H, =C**H**-CH$_2$-), 6.33 (d, J = 7.1 Hz, 1H, =C**H**-O-), 7.68 (s, 1H, **H**-Ar); ^{13}C NMR (CDCl$_3$, 75 MHz, δ/ppm) 17.78, 55.97, 60.92, 74.31, 96.53, 110.73, 119.50, 124.90, 125.48, 131.43, 141.42, 143.08, 149.39, 153.70; Anal. calcd for $C_{14}H_{16}ClNO_5$ C, 53.60; H, 5.14; N, 4.46; found C, 53.58; H, 5.21; N, 4.52.

1-((*E*)-2-((*E*)-But-2-en-1-yloxy)vinyl)-3-chloro-4,5-dimethoxy-2-nitrobenzene ((*E*,*E*)-2a).

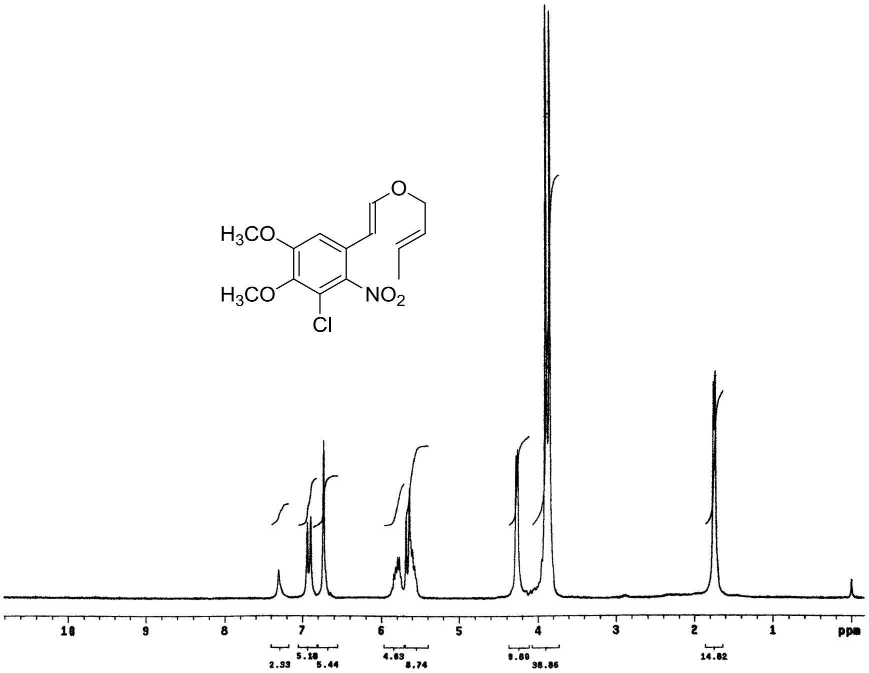

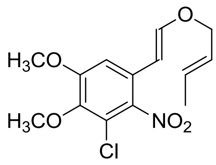

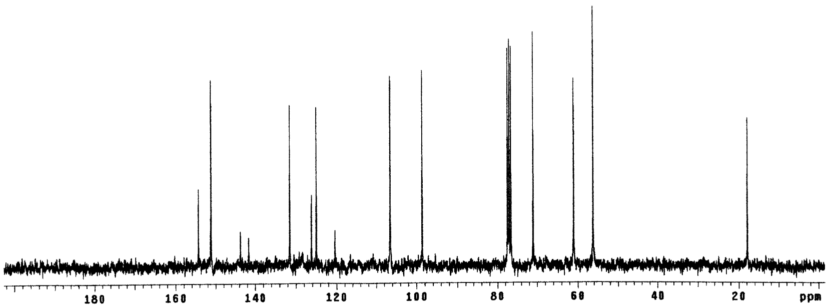

IR ($CHCl_3$) ν/cm^{-1} 2928.0, 1643.4, 1527.6, 1363.7, 1184.3; ^{1}H NMR ($CDCl_3$, 300 MHz, δ/ppm) 1.74 (d, J = 6.0 Hz, 3H, **H**$_3$C-CH=), 3.85 (s, 3H, **H**$_3$CO-Ar), 3.90 (s, 3H, **H**$_3$CO-Ar), 4.27 (d, J = 5.7 Hz, 2H, -**H**$_2$C-O-), 5.57-5.68 (m, 1H, =C**H**-CH_3), 5.66 (d, J = 12.6 Hz, 1H, =C**H**-Ar), 5.77-5.84 (m, 1H, =C**H**-CH_2-), 6.73 (s, 1H, **H**-Ar), 6.91 (d, J = 12.6 Hz, 1H, =C**H**-O-); ^{13}C NMR ($CDCl_3$, 75 MHz, δ/ppm) 17.86, 56.23, 61.01, 70.98, 98.60, 106.50, 120.40, 124.99, 126.17, 131.49, 141.61, 143.72, 151.14, 154.29; Anal. calcd for $C_{14}H_{16}ClNO_5$ C, 53.60; H, 5.14; N, 4.46; found C, 53.68; H, 5.10; N, 4.51.

Data for compound 2b. Thick viscous liquid (91%); ((*Z*:*E*) = 1:1.42)

3-Bromo-1-((*Z*)-2-((*E*)-but-2-en-1-yloxy)vinyl)-4,5-dimethoxy-2-nitrobenzene ((*Z*,*E*)-2b).

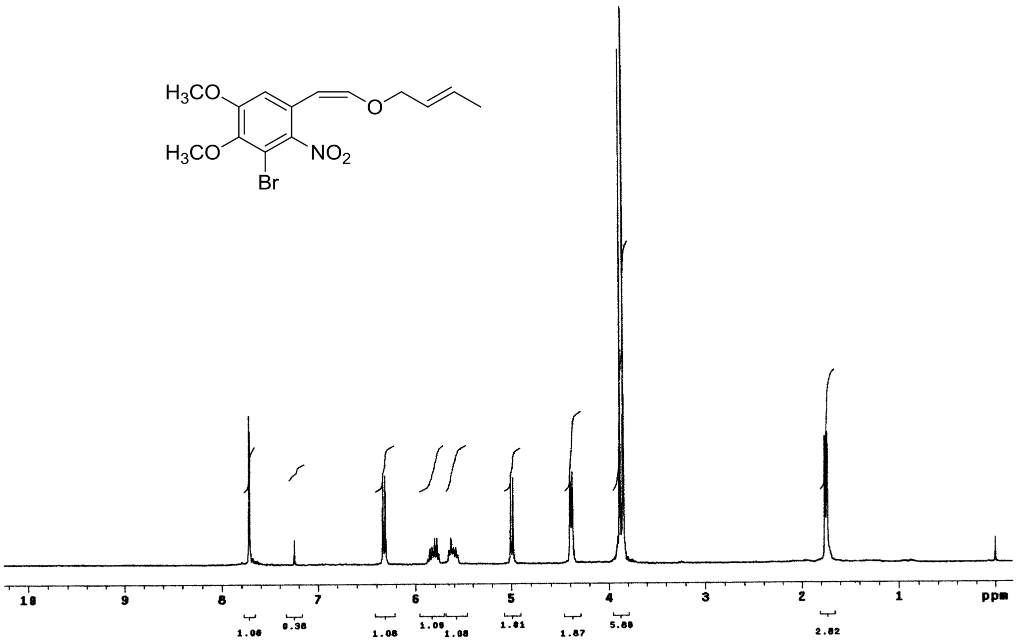

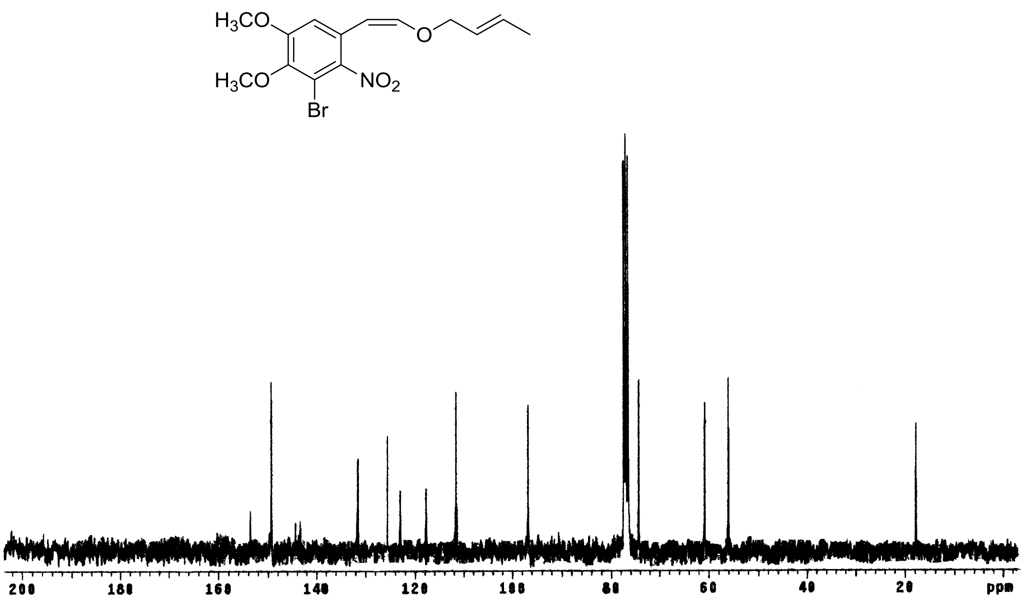

IR ($CHCl_3$) ν/cm^{-1} 2927.7, 1645.2, 1533.3, 1361.7, 1215.1, 1028.0; ^{1}H NMR ($CDCl_3$, 300 MHz, δ/ppm) 1.74 (d, *J* = 6.3 Hz, 3H, **H**$_3$C-CH=), 3.85 (s, 3H, **H**$_3$CO-Ar), 3.88 (s, 3H, **H**$_3$CO-Ar), 4.38 (d, *J* = 6.0 Hz, 2H, **-H**$_2$C-O-), 4.99 (d, *J* = 7.1 Hz, 1H, =C**H**-Ar), 5.55-5.65 (m, 1H, =C**H**-CH_3), 5.76-5.87 (m, 1H, =C**H**-CH_2-), 6.32 (d, *J* = 7.1 Hz, 1H, =C**H**-O-), 7.72 (s, 1H, Ar-**H**); ^{13}C NMR ($CDCl_3$, 75 MHz, δ/ppm) 17.84, 56.05, 60.84, 74.35, 96.82, 111.66, 118.01, 123.23, 125.55, 131.49, 143.34, 144.35, 149.31, 153.58; Anal. calcd for $C_{14}H_{16}BrNO_5$ C, 46.95; H, 4.50; N, 3.91; found C, 47.03; H, 4.47; N, 3.85.

3-Bromo-1-((*E*)-2-((*E*)-but-2-en-1-yloxy)vinyl)-4,5-dimethoxy-2-nitrobenzene ((*E*,*E*)-2b).

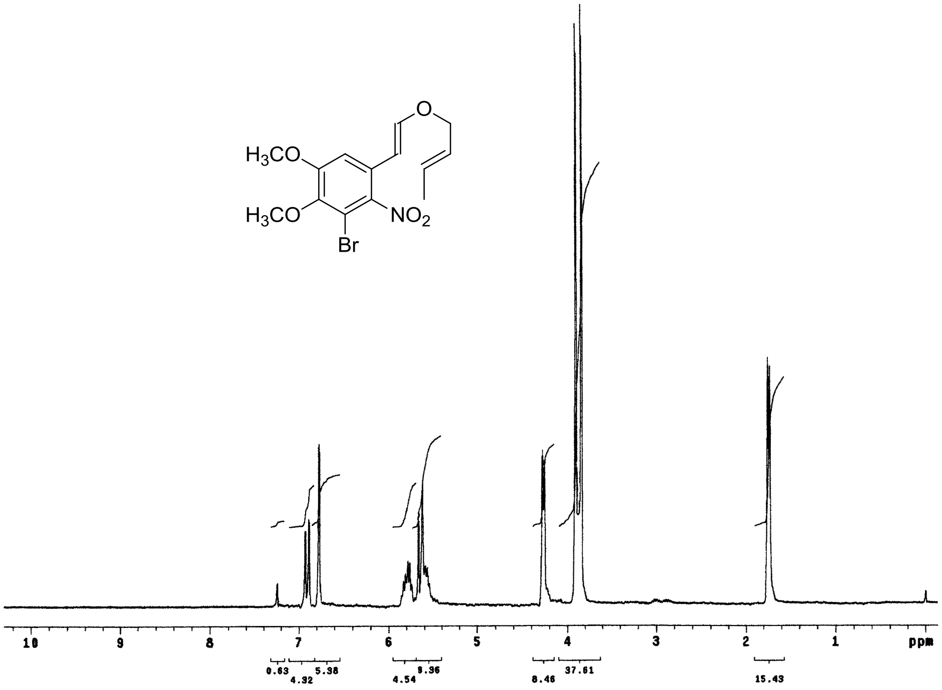

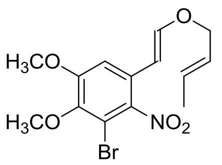

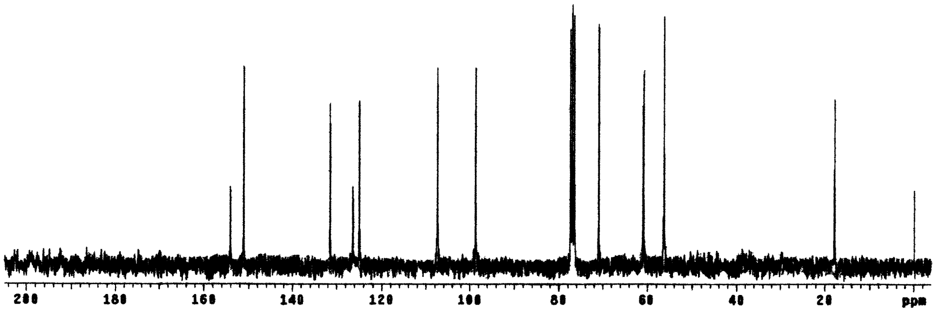

IR ($CHCl_3$) ν/cm^{-1} 2862.2, 1648.2, 1533.3, 1361.7, 1286.4, 1083.9; ^{1}H NMR ($CDCl_3$, 300 MHz, δ/ppm) 1.74 (d, *J* = 6.3 Hz, 3H, **H**$_3$C-CH=), 3.83 (s, 3H, **H**$_3$CO-Ar), 3.84 (s, 3H, **H**$_3$CO-Ar), 4.26 (d, *J* = 6.0 Hz, 2H, **-H**$_2$C-O-), 5.48-5.58 (m, 1H, =C**H**-CH$_3$), 5.64 (d, *J* = 12.6 Hz, 1H, =C**H**-Ar), 5.70-5.83 (m, 1H, =C**H**-CH$_2$-), 6.77 (s, 1H, Ar-**H**), 6.90 (d, *J* = 12.6 Hz, 1H,=C**H**-O-); ^{13}C NMR ($CDCl_3$, 75 MHz, δ/ppm) 17.86, 56.26, 60.85, 70.96, 98.65, 107.38, 124.98, 126.40, 131.48, 151.14, 154.11; Anal. calcd for $C_{14}H_{16}BrNO_5$ C, 46.95; H, 4.50; N, 3.91; found C, 47.02; H, 4.49; N, 3.87.

Data for compound 2c. Thick viscous liquid (92%); ((*Z*:*E*) = 1:1.87)

1-((*Z*)-2-((*E*)-But-2-en-1-yloxy)vinyl)-3,4,5-trimethoxy-2-nitrobenzene ((*Z*,*E*)-2c).

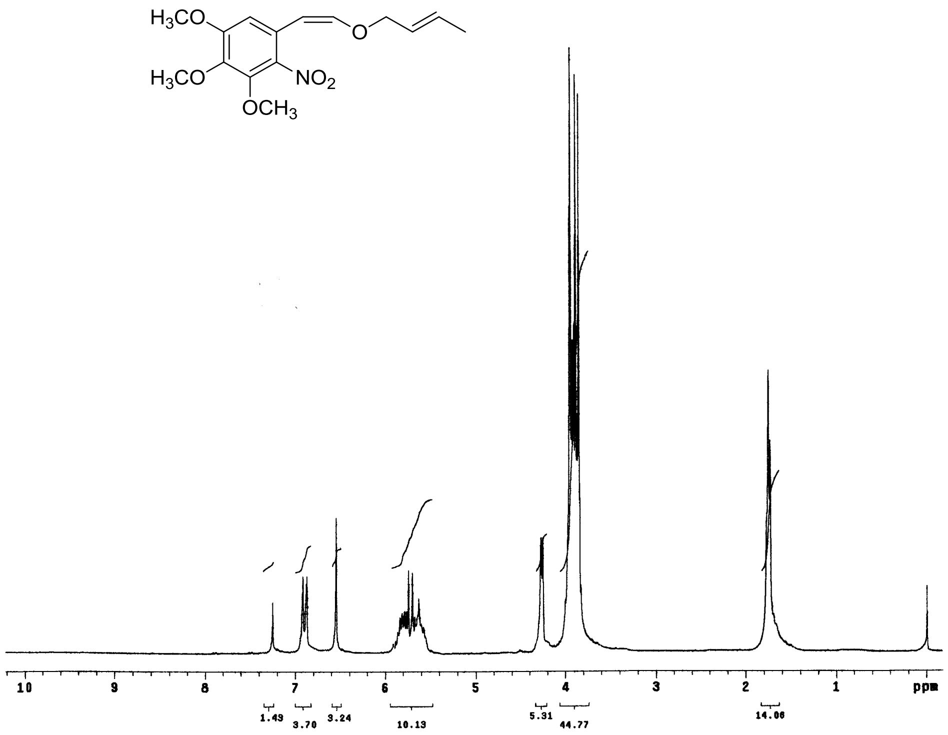

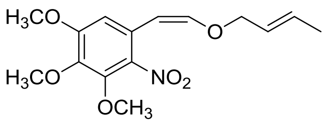

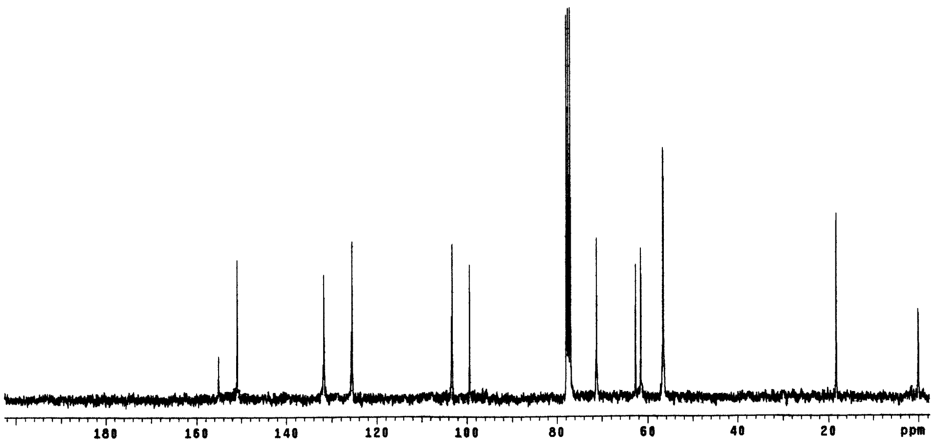

IR ($CHCl_3$) ν/cm^{-1} 2929.9, 1639.5, 1523.8, 1361.7, 1269.2, 1120.6; ^{1}H NMR ($CDCl_3$, 300 MHz, δ/ppm) 1.74 (d, *J* = 5.5 Hz, 3H, **H**$_3$C-CH=), 3.95 (s, 3H, **H**$_3$CO-Ar), 3.97 (s, 3H, **H**$_3$CO-Ar), 3.99 (s, 3H, **H**$_3$CO-Ar), 4.26 (d, *J* = 5.7 Hz, 2H, -C**H**$_2$-O-), 5.55-5.67 (m, 1H, =C**H**-CH_3), 5.72 (d, *J* = 12.6 Hz, 1H, =C**H**-Ar), 5.76-5.86 (m, 1H, =C**H**-CH_2-), 6.54 (s, 1H, **H**-Ar), 6.89 (d, *J* = 12.6 Hz, 1H, =C**H**-O-); ^{13}C NMR ($CDCl_3$, 75 MHz, δ/ppm) 18.30, 56.68, 61.54, 62.67, 71.18, 99.41, 103.23, 125.54, 131.77, 150.88, 155.10; Anal. calcd for $C_{15}H_{19}NO_6$ C, 58.25; H, 6.19; N, 4.53; found C, 58.21; H, 6.26; N, 4.49.

1-((*E*)-2-((*E*)-But-2-en-1-yloxy)vinyl)-3,4,5-trimethoxy-2-nitrobenzene ((*E*,*E*)-2c).

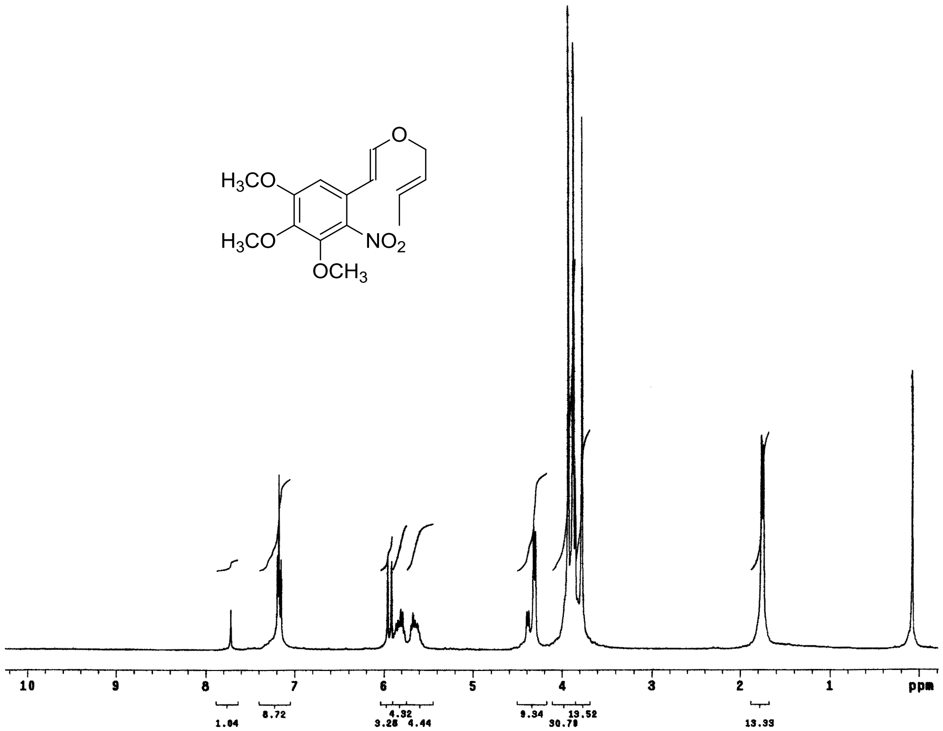

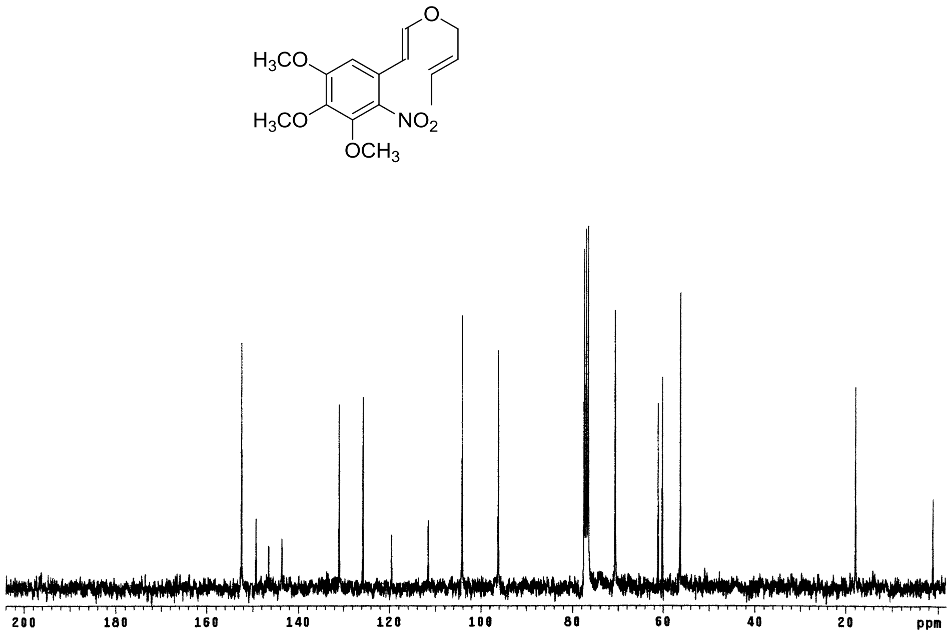

IR ($CHCl_3$) ν/cm^{-1} 2939.1, 1641.3, 1531.3, 1363.5, 1213.1, 1026.0; ^{1}H NMR ($CDCl_3$, 300 MHz, δ/ppm) 1.75 (d, *J* = 6.0 Hz, 3H, **H**$_3$C-CH=), 3.77 (s, 3H, **H**$_3$CO-Ar), 3.86 (s, 3H, **H**$_3$CO-Ar), 3.89 (s, 3H, **H**$_3$CO-Ar), 4.31 (d, *J* = 6.0 Hz, 2H, **-H**$_2$C-O-), 5.60-5.76 (m, 1H, =C**H**-CH_3), 5.78-5.88 (m, 1H, =C**H**-CH_2-), 5.93 (d, *J* = 12.6 Hz, 1H, =C**H**-Ar), 7.16 (d, *J* = 12.6 Hz, 1H, =C**H**-O-), 7.18 (s, 1H, **H**-Ar); ^{13}C NMR ($CDCl_3$, 75 MHz, δ/ppm) 17.90, 56.23, 60.19, 61.13, 70.63, 96.15, 104.16, 111.60, 119.51, 125.70, 130.96, 143.61, 146.50, 149.31, 152.41; Anal. calcd for $C_{15}H_{19}NO_6$ C, 58.25; H, 6.19; N, 4.53; found C, 58.18; H, 6.26; N, 4.49.

Data for compound 2d. Thick viscous liquid, (93%); ((*Z*:*E*) = 1:1.93)

5-((*Z*)-2-((*E*)-But-2-en-1-yloxy)vinyl)-6-nitrobenzo[*d*][1,3]dioxole ((*Z*,*E*)-2d).

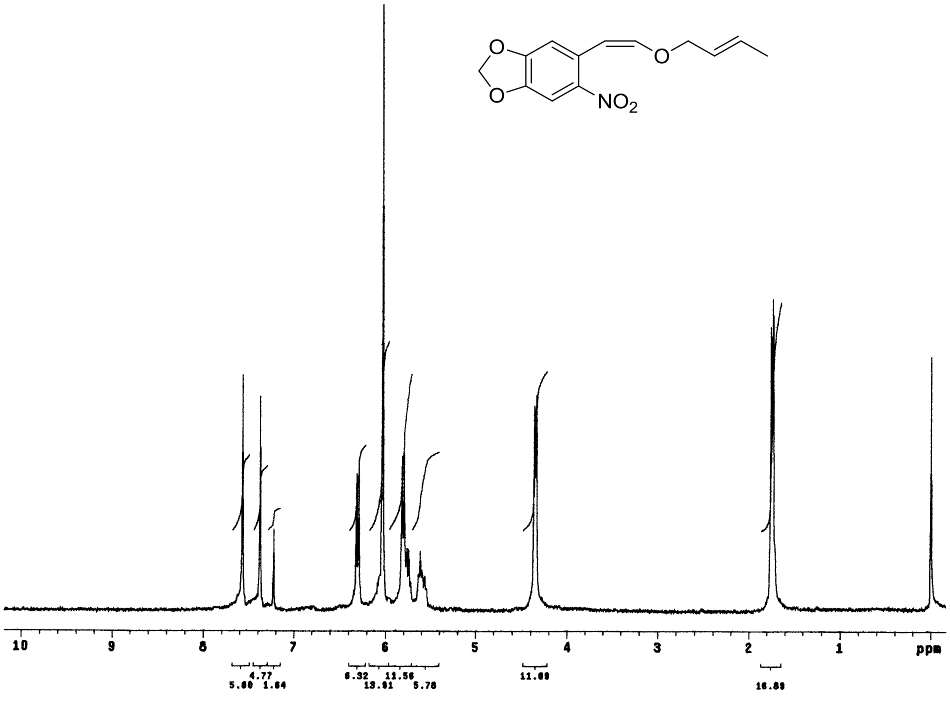

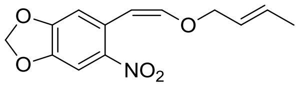

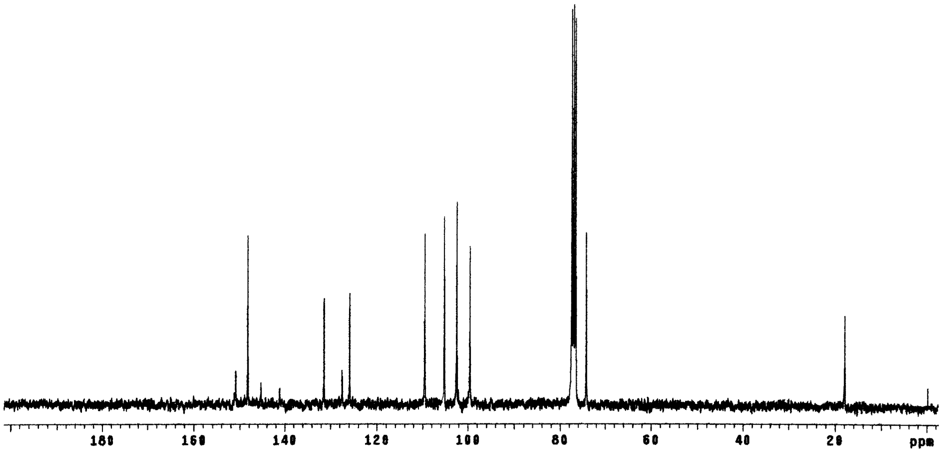

IR ($CHCl_3$) ν/cm^{-1} 2920.3, 1635.6, 1508.3, 1329.0, 1255.7, 1035.8; ^{1}H NMR ($CDCl_3$, 300 MHz, δ/ppm) 1.74 (d, *J* = 6.3 Hz, 3H, **H**$_3$C-CH=), 4.35 (d, *J* = 6.3 Hz, 2H, **-H**$_2$C-O-), 5.56-5.61 (m, 1H, =C**H**-CH_3), 5.74-5.81 (m, 1H, =C**H**-CH_2-), 5.80 (d, *J* = 7.3 Hz, 1H, =C**H**-Ar), 6.03 (s, 2H, -O-C**H**$_2$-O-), 6.30 (d, *J* = 7.3 Hz, 1H, =C**H**-O-), 7.37 (s, 1H, Ar-**H**), 7.57 (s, 1H, Ar-**H**); ^{13}C NMR ($CDCl_3$, 75 MHz, δ/ppm) 17.90, 74.23, 99.53, 102.43, 105.20, 109.54, 125.84, 127.45, 131.33, 141.11, 145.32, 148.23, 150.93; Anal. calcd for $C_{13}H_{13}NO_5$ C, 59.31; H, 4.98; N, 5.32; found C, 59.39; H, 5.03; N, 5.24.

5-((*E*)-2-((*E*)-but-2-en-1-yloxy)vinyl)-6-nitrobenzo[*d*][1,3]dioxole ((*E*,*E*)-2d).

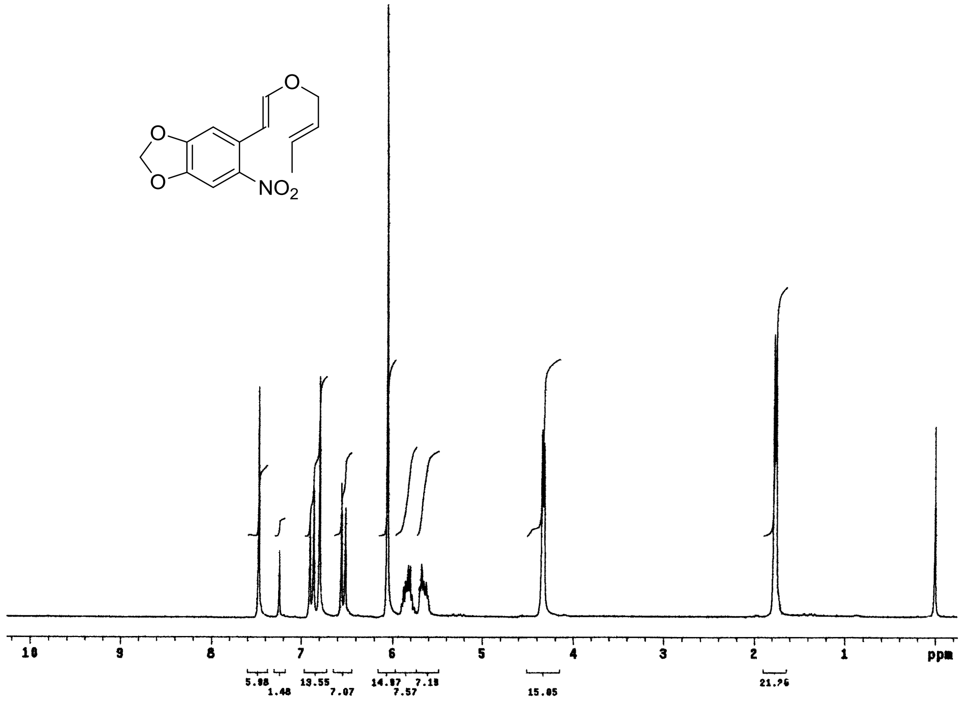

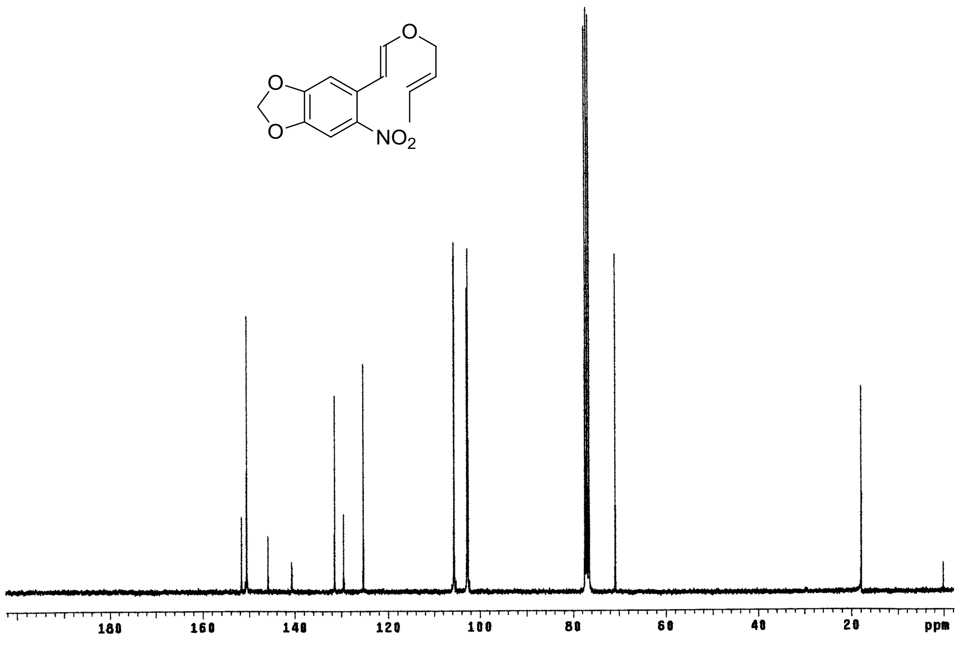

IR ($CHCl_3$) ν/cm^{-1} 2918.4, 1627.9, 1514.1, 1329.0, 1257.6, 1037.7; ^{1}H NMR ($CDCl_3$, 300 MHz, δ/ppm) 1.76 (d, *J* = 6.3 Hz, 3H, **H**$_3$C-CH=), 4.33 (d, *J* = 5.7 Hz, 2H, **-H**$_2$C-O-), 5.60-5.69 (m, 1H, =C**H**-CH_3), 5.78-5.87 (m, 1H, =C**H**-CH_2-), 6.05 (s, 2H, -O-C**H**$_2$-O-), 6.53 (d, *J* = 12.6 Hz, 1H, =C**H**-Ar), 6.79 (s, 1H, Ar-**H**), 6.88 (d, *J* = 12.6 Hz, 1H, =C**H**-O-), 7.47 (s, 1H, Ar-**H**); ^{13}C NMR ($CDCl_3$, 75 MHz, δ/ppm) 17.92, 70.82, 102.61, 102.84, 105.61, 125.31, 129.50, 131.40, 140.68, 145.89, 150.56, 151.69; Anal. calcd for $C_{13}H_{13}NO_5$ C, 59.31; H, 4.98; N, 5.32; found C, 59.38; H, 5.06; N, 5.30.

Data for compound 2e. Thick viscous liquid, (91%)

1-(2-((*E*)-But-2-en-1-yloxy)vinyl)-2-nitrobenzene ((*E*)-2e).

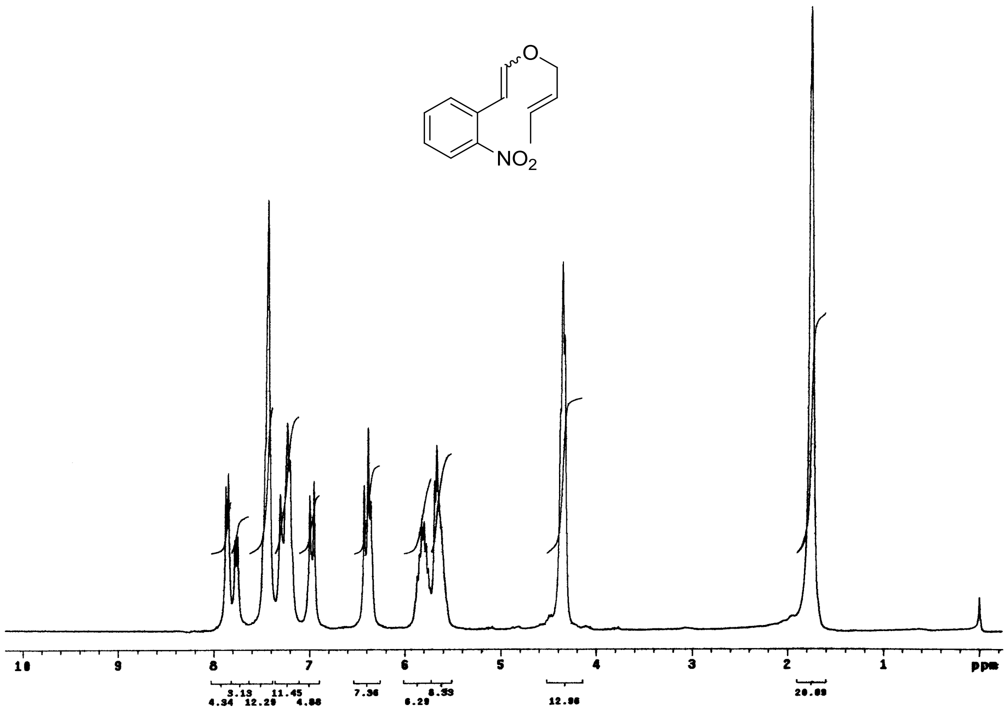

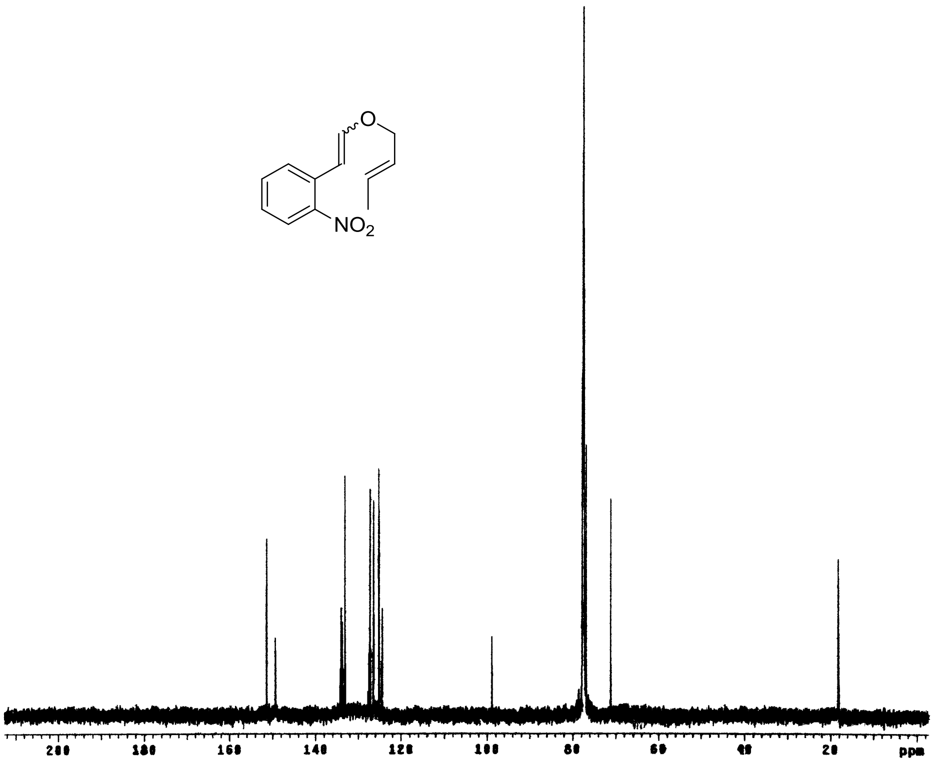

IR ($CHCl_3$) ν/cm^{-1} 3022.2, 1608.5, 1535.3, 1377.1, 1039.6; ^{1}H NMR ($CDCl_3$, 300 MHz, δ/ppm) 1.75 (bs, 6H, 2 × **H**$_3$C-CH=), 4.35 (bs, 4H, 2 × **-H**$_2$C-O-), 5.66-5.68 (m, 2H, 2 × =C**H**-CH$_3$), 5.75-5.87 (m, 2H, 2 × =C**H-**CH$_2$**-**), 6.35-6.43 (m, 2H, 2 × =C**H-**Ar), 6.97 (d, *J* = 12.5 Hz, 2H, 2 × =C**H-**O**-**), 7.20-7.30 (m, 2H, 2 × Ar-**H**), 7.42 (bs, 4H, 4 × Ar-**H**), 7.85 (dd, *J* = 7.9 and 7.7 Hz, 2H, 2 × Ar-**H**); ^{13}C NMR ($CDCl_3$, 75 MHz, δ/ppm) 17.91, 71.30, 98.10, 98.20, 124.11, 124.37, 125.21, 125.32, 126.23, 126.38, 127.13, 127.29, 132.98, 133.10, 133.61, 133.71, 149.21, 152.90; Anal. calcd for $C_{12}H_{13}NO_3$ C, 65.74; H, 5.98; N, 6.39; found C, 65.85; H, 6.07; N, 6.36.

Data for compound 2f. Thick viscous liquid, (90%)

1-Bromo-2-(2-((*E*)-but-2-en-1-yloxy)vinyl)-4,5,6-trimethoxy-3-nitrobenzene ((*E*)-2f).

(only (*E*)-isomer)

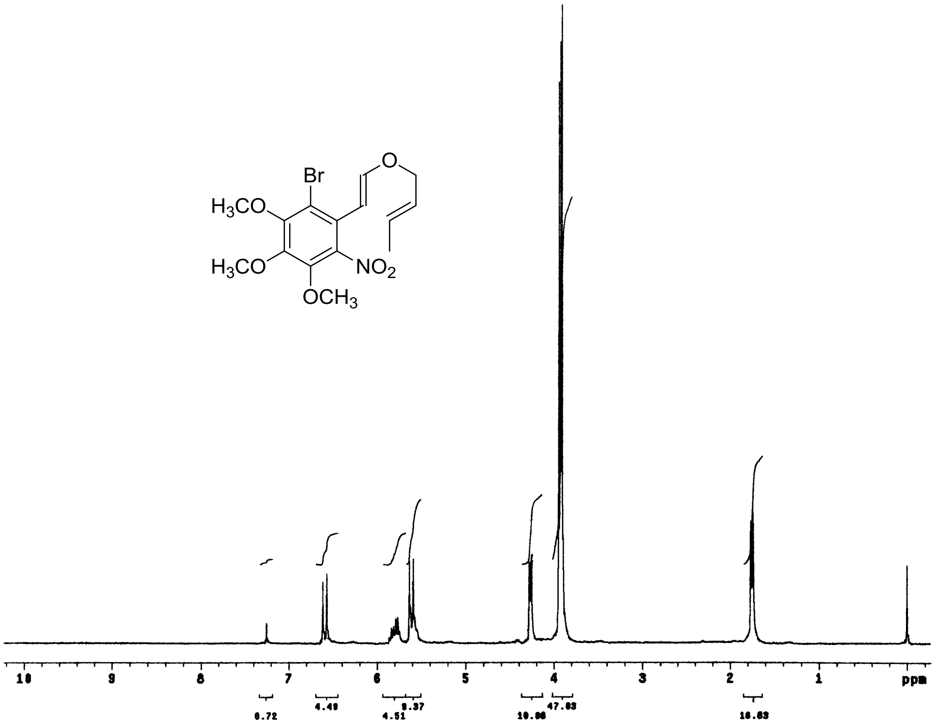

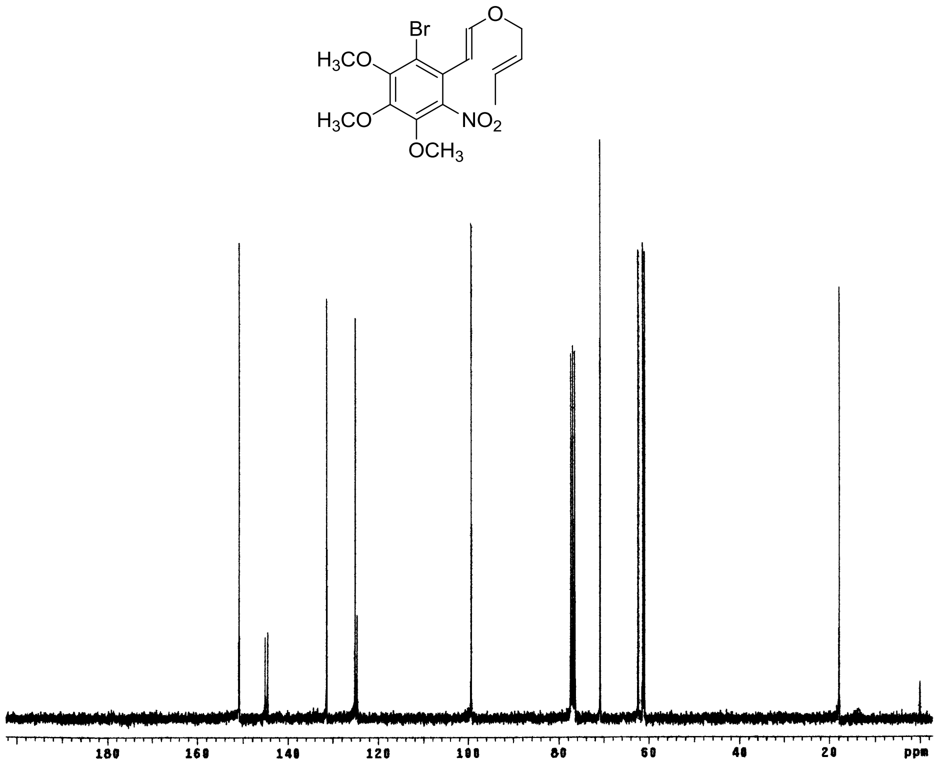

IR ($CHCl_3$) ν/cm^{-1} 2943.4, 1654.9, 1537.3, 1365.6, 1232.5, 1024.2; ^{1}H NMR ($CDCl_3$, 300 MHz, δ/ppm) 1.75 (d, *J* = 6.3 Hz, 3H, **H**$_3$C-CH=), 3.90 (s, 3H, **H**$_3$CO-Ar), 3.92 (s, 3H, **H**$_3$CO-Ar), 3.94 (s, 3H, **H**$_3$CO-Ar), 4.27 (d, *J* = 6.3 Hz, 2H, -**H**$_2$C-O-), 5.54-5.63 (m, 1H, =C**H**-CH_3), 5.61 (d, *J* = 12.9 Hz, 1H, =C**H**-Ar), 5.74-5.84 (m, 1H, =C**H**-CH_2-), 6.59 (d, *J* = 12.9 Hz, 1H, =C**H**-O-); ^{13}C NMR ($CDCl_3$, 75 MHz, δ/ppm) 17.89, 61.03, 61.35, 62.37, 70.88, 99.38, 124.71, 125.14, 131.41, 144.64, 145.19, 150.93; Anal. calcd for $C_{15}H_{18}BrNO_6$ C, 46.41; H, 4.67; N, 3.61; found C, 46.49; H, 4.76; N, 3.58.

Data for compound 2g. Thick viscous liquid, (92), ((*Z*:*E*) ratio = 1:2.06)

2-(2-((*E*)-But-2-en-1-yloxy)vinyl)-4-methoxy-1-nitrobenzene ((*E*)-82g).

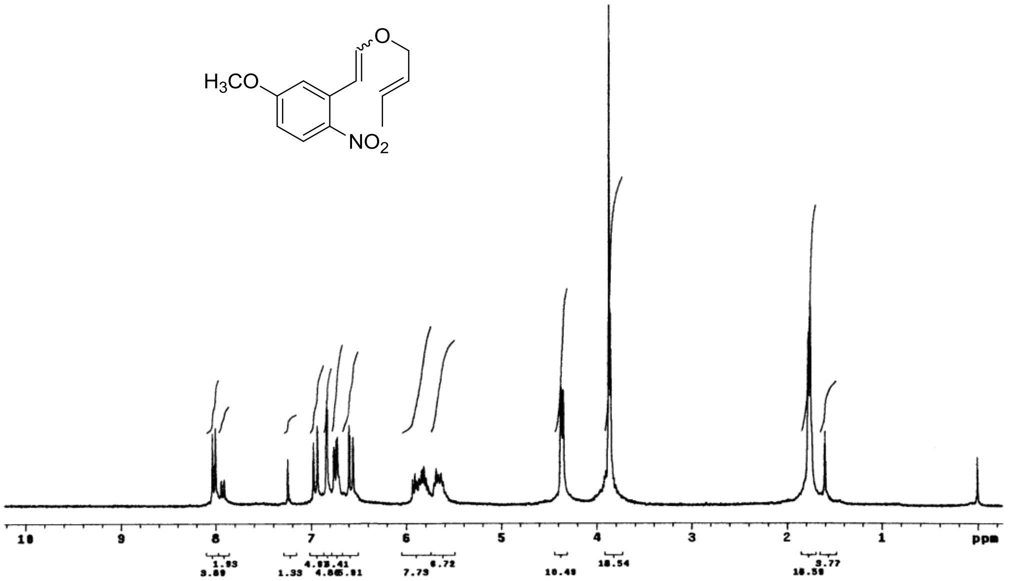

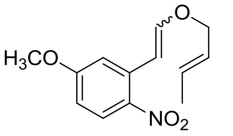

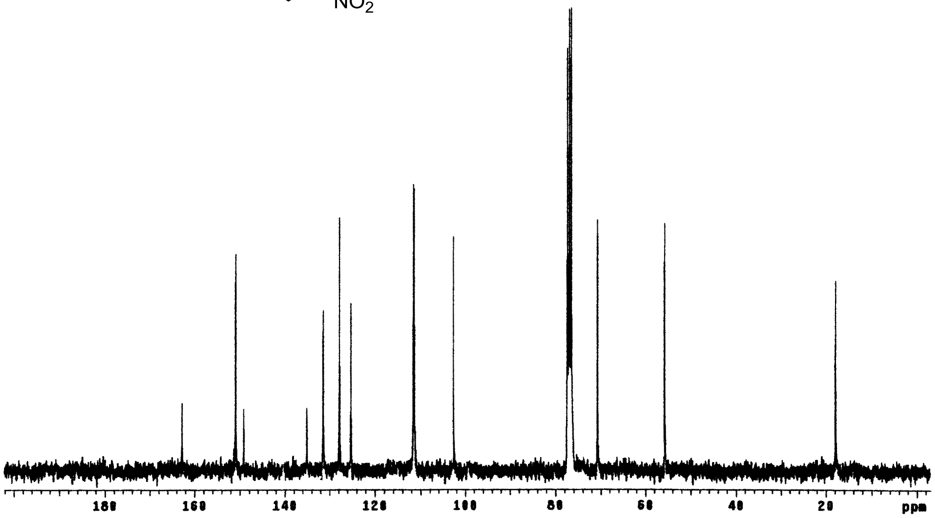

IR ($CHCl_3$) ν/cm^{-1} 2854.7, 1606.7, 1510.3, 1338.6, 1244.1, 1087.8; ^{1}H NMR ($CDCl_3$, 300 MHz, δ/ppm) 1.61 (d, *J* = 6.4 Hz, 3H, **H**$_3$C-CH=), 1.77 (d, *J* = 6.3 Hz, 3H, **H**$_3$C-CH=), 3.86 (s, 3H, **H**$_3$CO-Ar), 3.88 (s, 3H, **H**$_3$CO-Ar), 4.36 (d, *J* = 6.3 Hz, 4H, 2 × -**H**$_2$C-O-), 5.62-5.80 (m, 2H, 2 × =C**H**-CH$_3$), 5.82-5.93 (m, 2H, 2 × =C**H**-CH$_2$-), 6.57 (d, *J* = 12.6 Hz, 2H, 2 × =C**H**-Ar), 6.76 (d, *J* = 2.4 Hz, 2H, 2 × **H**-Ar), 6.83 (d, *J* = 2.4 Hz, 2H, 2 × **H**-Ar), 6.95 (d, *J* = 12.6 Hz, 2H, 2 × =C**H**-O-), 7.93 (d, *J* = 9.0 Hz, 2H, 2 × **H**-Ar), 8.02 (d, *J* = 9.0 Hz, 2H, 2 × **H**-Ar); ^{13}C NMR ($CDCl_3$, 75 MHz, δ/ppm) 17.94, 55.63, 55.77, 70.78, 102.69, 111.46, 111.61, 125.24, 127.77, 131.43, 135.07, 149.13, 150.92, 162.78; Anal. calcd for $C_{13}H_{15}NO_4$ C, 62.64; H, 6.07; N, 5.62; found C, 62.62; H, 6.12; N, 5.59.

Data for compound 2h. Thick viscous liquid, (91%), ((*Z*:*E*) ratio = 1:1.94)

2-(2-((*E*)-But-2-en-1-yloxy)vinyl)-1-chloro-3-nitrobenzene ((*E*)-2h).

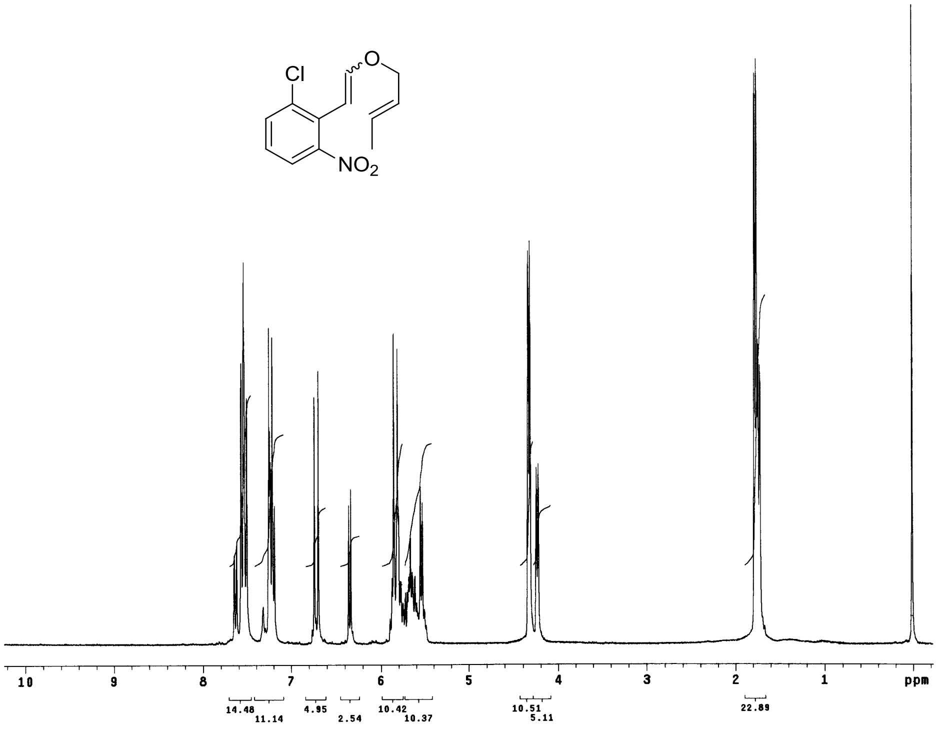

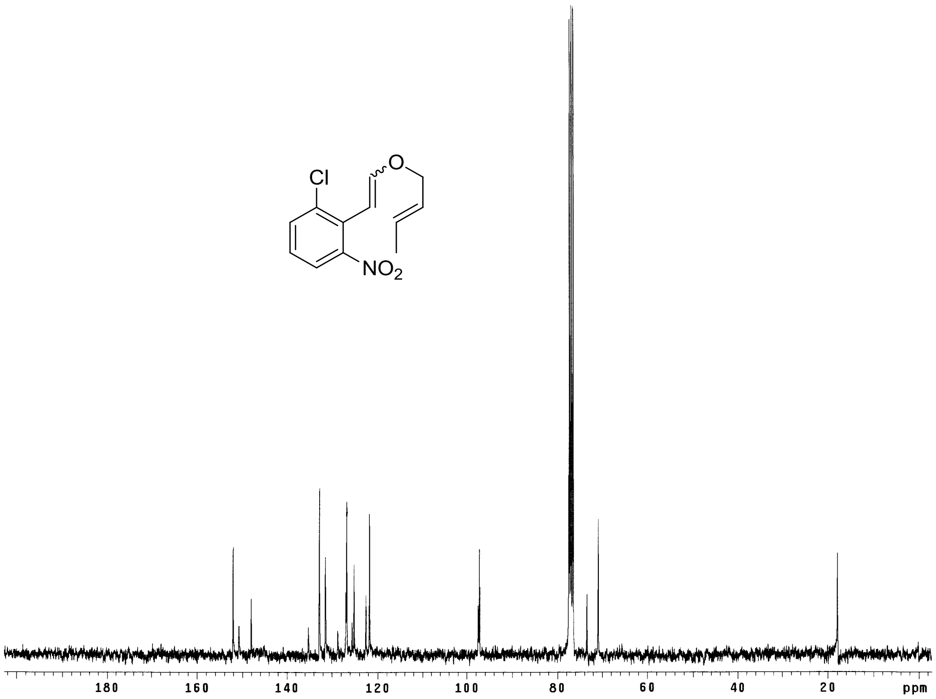

IR ($CHCl_3$) ν/cm^{-1} 2920.3, 1651.1, 1529.6, 1361.7, 1120.6; ^1H NMR ($CDCl_3$, 300 MHz, δ/ppm) 1.74 (d, *J* = 3.3 Hz, 3H, **H**$_3$C-CH=), 1.76 (d, *J* = 3.3 Hz, 3H, **H**$_3$C-CH=), 4.23 (d, *J* = 6.3 Hz, 2H, **-H**$_2$C-O-), 4.33 (d, *J* = 6.3 Hz, 2H, **-H**$_2$C-O-), 5.50-5.87 (m, 4H, 2 × =C**H-**CH_3 and 2 × =C**H-**CH_2**-**), 5.53 (d, *J* = 6.8 Hz, 1H, =C**H-**Ar), 5.83 (d, *J* = 13.2 Hz, 1H, =C**H-**Ar), 6.36 (d, *J* = 6.8 Hz, 1H, =C**H-**O-), 6.75 (d, *J* = 13.2 Hz, 1H, =C**H-**O-), 7.19-7.24 (m, 2H, 2 × Ar-**H**), 7.52-7.73 (m, 4H, 4 × Ar-**H**); ^{13}C NMR ($CDCl_3$, 75 MHz, δ/ppm) 17.94, 70.86, 73.42, 97.22, 97.49, 121.82, 122.55, 125.18, 125.61, 126.79, 126.97, 131.45, 132.76, 135.20, 148.06, 150.78, 152.09; Anal. calcd for $C_{12}H_{12}ClNO_3$ C, 56.81; H, 4.77; N, 5.52; found C, 56.87; H, 4.85; N, 5.45.

Data for compound 3a. Thick viscous liquid, (85%), diastereomeric ratio = 1:1.21

2-(3-Chloro-4,5-dimethoxy-2-nitrophenyl)-3-methylpent-4-enal (3a).

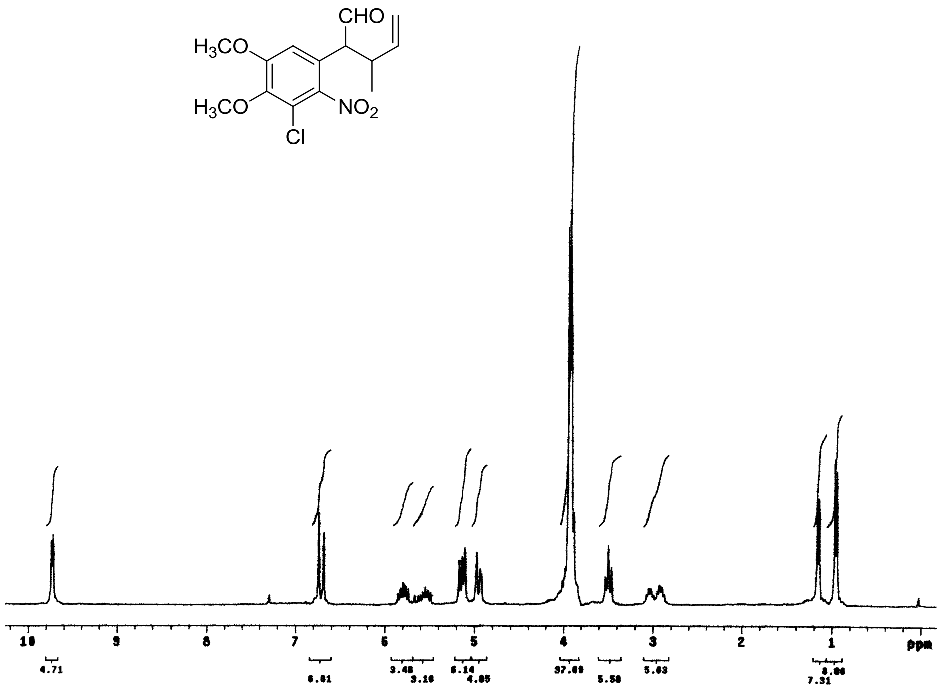

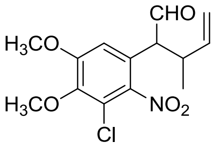

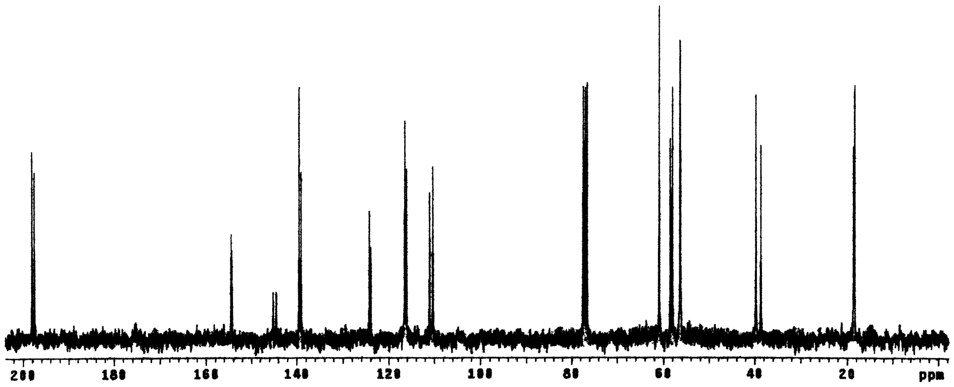

IR ($CHCl_3$) ν/cm^{-1} 2928.0, 1720.5, 1645.3, 1535.3, 1363.7, 1219.0; ^{1}H NMR ($CDCl_3$, 300 MHz, δ/ppm) 0.95 (d, *J* = 6.6 Hz, 3H, **H**$_3$C-CH-), 1.18 (d, *J* = 6.6 Hz, 3H, **H**$_3$C-CH-), 2.87-3.07 (m, 2H, 2 × -C**H**-CH$_3$), 3.49 (t, *J* = 10.1 Hz, 2H, 2 × -C**H**-Ar), 3.91 (s, 3H, OC**H**$_3$-Ar), 3.93 (s, 3H, OC**H**$_3$-Ar), 3.95 (s, 3H, OC**H**$_3$-Ar), 3.98 (s, 3H, OC**H**$_3$-Ar), 4.92-4.97 (m, 2H, **H**$_2$C=CH-), 5.10-5.16 (m, 2H, **H**$_2$C=CH-), 5.48-5.66 (m, 1H, -C**H**=CH$_2$), 5.73-5.85 (m, 1H, -C**H**=CH$_2$), 6.68 (s, 1H, **H**-Ar), 6.73 (s, 1H, **H**-Ar), 9.73 (d, *J* = 4.9 Hz, 2H, 2 × -C**H**O); ^{13}C NMR ($CDCl_3$, 75 MHz, δ/ppm) 18.36, 18.59, 38.63, 39.71, 56.36, 56.42, 58.07, 58.54, 60.96, 110.28, 111.00, 116.13, 116.49, 123.95, 124.25, 139.04, 139.47, 144.23, 145.08, 154.30, 154.48, 197.53, 198.04; Anal. calcd for $C_{14}H_{16}ClNO_5$ C, 53.60; H, 5.14; N, 4.46; found C, 53.68; H, 5.20; N, 4.38.

Data for compound 3b. Thick viscous liquid, (83%), diastereomeric ratio = 1:1.36

2-(3-Bromo-4,5-dimethoxy-2-nitrophenyl)-3-methylpent-4-enal (3b).

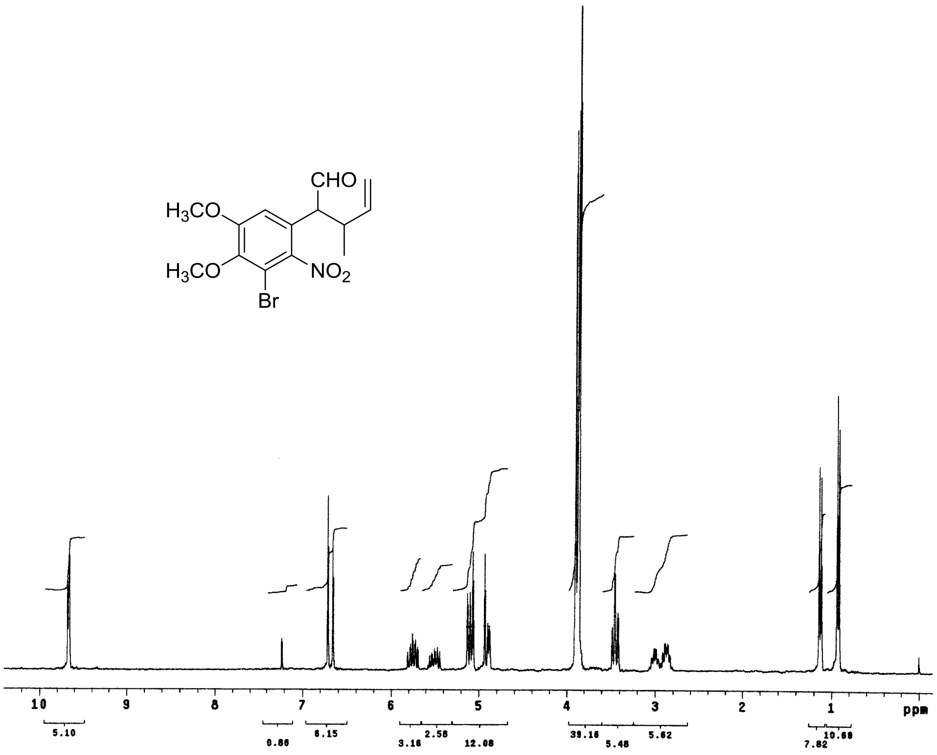

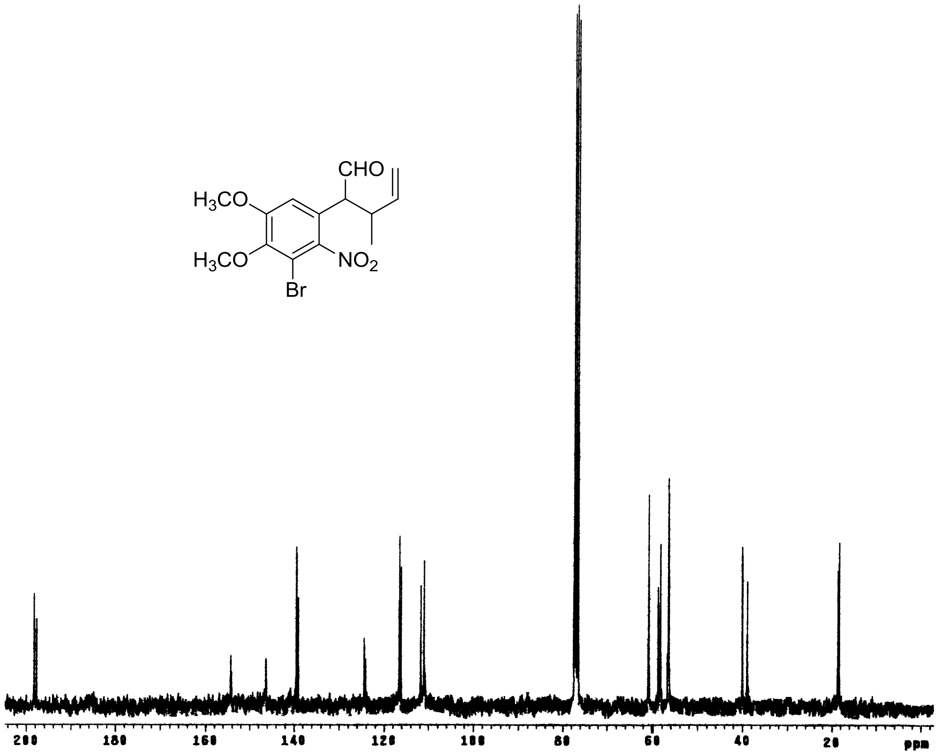

IR ($CHCl_3$) ν/cm^{-1} 2929.6, 1726.1, 1533.3, 1361.6, 1215.0, 1022.2; ^{1}H NMR ($CDCl_3$, 300 MHz, δ/ppm) 0.92 (d, *J* = 6.4 Hz, 3H, **H**$_3$C-CH-), 1.12 (d, *J* = 6.4 Hz, 3H, **H**$_3$C-CH-), 2.83-3.04 (m, 2H, 2 × -C**H**-CH$_3$), 3.41-3.48 (m, 2H, 2 × -C**H**-Ar), 3.86 (s, 6H, 2 × **H**$_3$CO-Ar), 3.87 (s, 3H, **H**$_3$CO-Ar), 3.90 (s, 3H, **H**$_3$CO-Ar), 4.88-4.95 (m, 2H, **H**$_2$C=CH-), 5.06-5.12 (m, 2H, **H**$_2$C=CH-), 5.44-5.55 (m, 1H, -C**H**=CH$_2$), 5.69-5.81 (m, 1H, -C**H**=CH$_2$), 6.66 (s, 1H, **H**-Ar), 6.72 (s, 1H, **H**-Ar), 9.67 (d, *J* = 5.3 Hz, 2H, 2 × -C**H**O); ^{13}C NMR ($CDCl_3$, 75 MHz, δ/ppm) 18.42, 18.68, 38.70, 39.83, 56.41, 56.46, 58.26, 58.74, 60.88, 111.02, 111.75, 116.23, 124.15, 124.46, 139.06, 139.47, 146.31, 154.15, 154.32, 197.71, 198.25; Anal. calcd for $C_{14}H_{16}BrNO_5$ C, 46.95; H, 4.50; N, 3.91; found C, 46.89; H, 4.42; N, 3.98.

Data for compound 3c. Thick viscous liquid, (84%), diastereomeric ratio = 1:1.74

3-Methyl-2-(3,4,5-trimethoxy-2-nitrophenyl)pent-4-enal (3c).

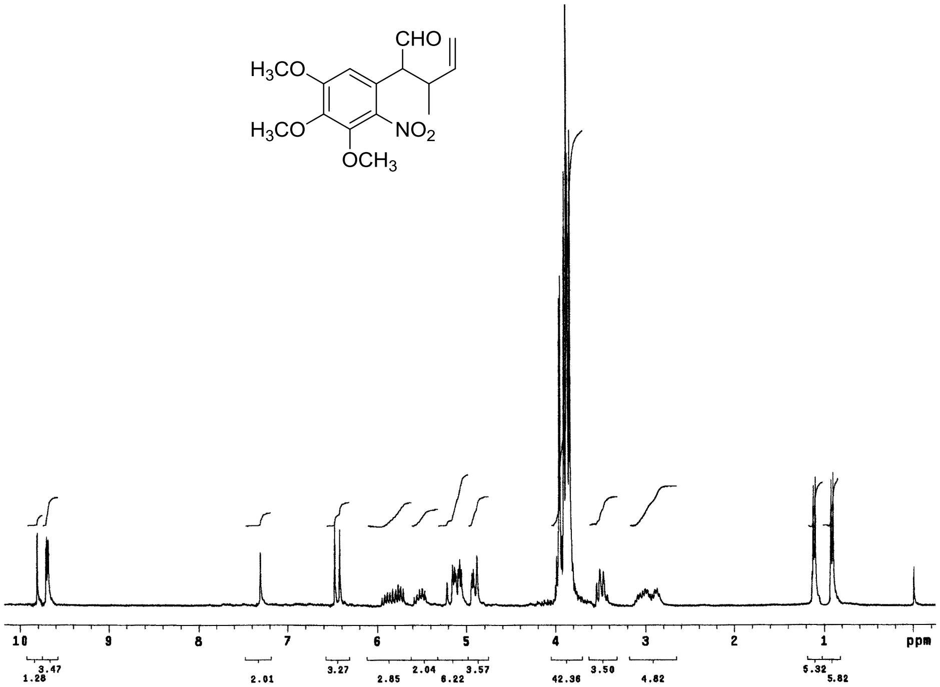

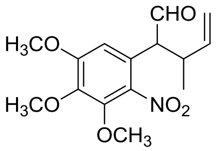

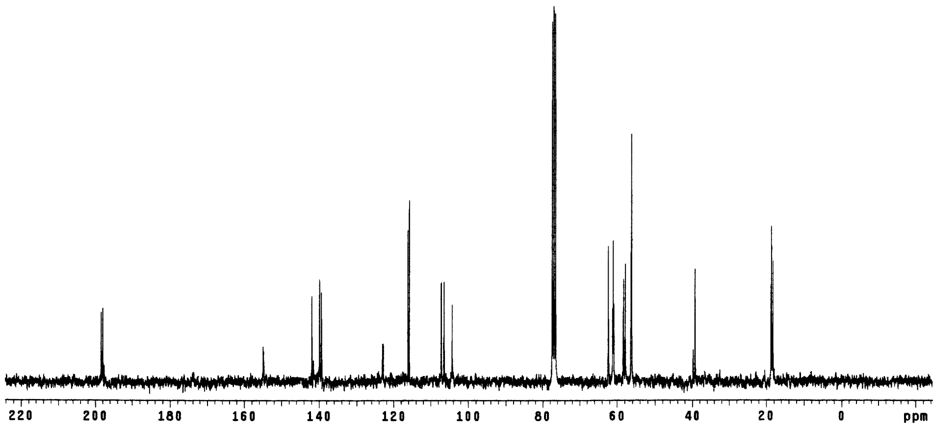

IR ($CHCl_3$) ν/cm^{-1} 2937.6, 1726.3, 1529.6, 1346.3, 1249.9, 1026.1; ^{1}H NMR ($CDCl_3$, 300 MHz, δ/ppm) 0.91 (d, *J* = 6.8 Hz, 3H, **H**$_3$C-CH-), 1.11 (d, *J* = 6.6 Hz, 3H, **H**$_3$C-CH-), 2.84-3.08 (m, 2H, 2 × -C**H**-CH_3), 3.42-3.54 (m, 2H, 2 × -C**H**-Ar), 3.86 (s, 6H, 2 × **H**$_3$CO-Ar), 3.895 (s, 3H, **H**$_3$CO-Ar), 3.899 (s, 3H, **H**$_3$CO-Ar), 3.904 (s, 3H, **H**$_3$CO-Ar), 3.907 (s, 3H, **H**$_3$CO-Ar), 4.88-4.94 (m, 2H, **H**$_2$C=CH-), 5.06-5.21 (m, 2H, **H**$_2$C=CH-), 5.45-5.58 (m, 1H, -C**H**=CH_2), 5.71-5.94 (m, 1H, -C**H**=CH_2), 6.42 (s, 1H, **H**-Ar), 6.48 (s, 1H, **H**-Ar), 9.71 (d, *J* = 1.3 Hz, 1H, -C**H**O), 9.81 (s, 1H, -C**H**O); ^{13}C NMR ($CDCl_3$, 75 MHz, δ/ppm) 18.68, 18.76, 38.41, 39.33, 56.27, 56.34, 57.89, 58.35, 61.06, 61.24, 62.36, 106.47, 107.20, 115.80, 116.19, 122.84, 123.09, 139.36, 139.88, 141.50, 141.92, 154.74, 154.93, 197.98, 198.41; Anal. calcd for $C_{15}H_{19}NO_6$ C, 58.25; H, 6.19; N, 4.53; found C, 58.32; H, 6.14; N, 4.62.

Data for compound 3d. Thick viscous liquid, (86%), diastereomeric ratio = 1:1.63

3-Methyl-2-(6-nitrobenzo[*d*][1,3]dioxol-5-yl)pent-4-enal (3d).

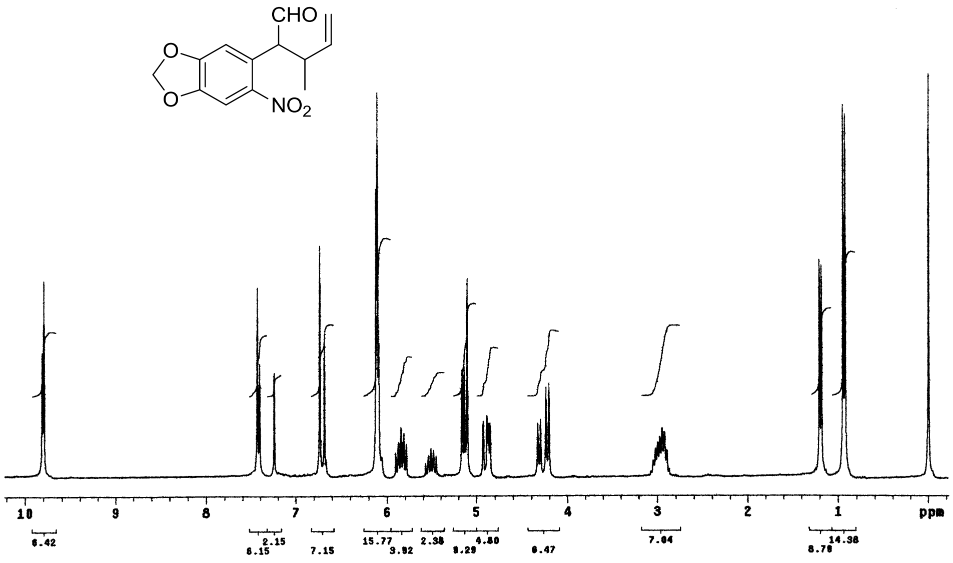

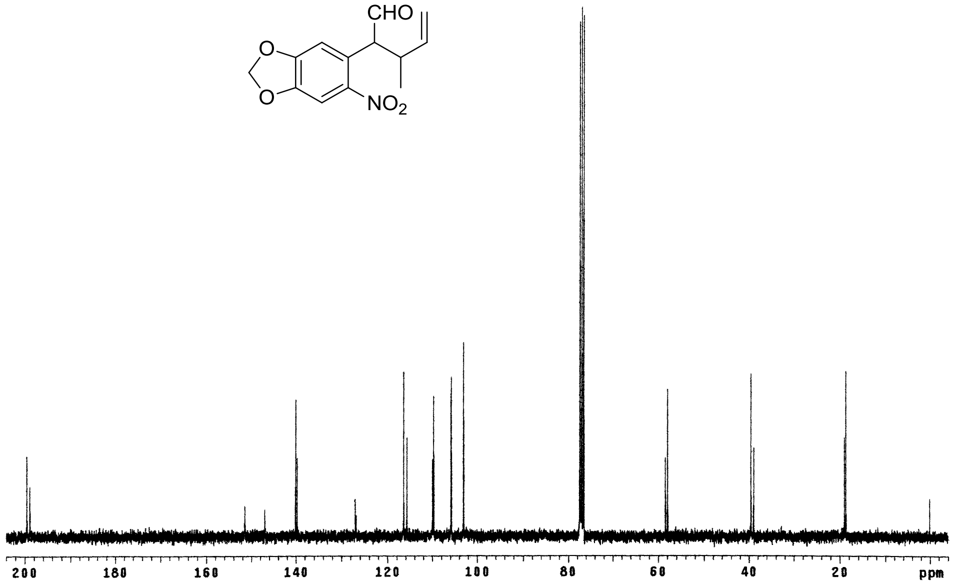

IR ($CHCl_3$) ν/cm^{-1} 2976.2, 2733.2, 1724.4, 1618.3, 1523.8, 1336.7, 1257.6, 1037.7; ^{1}H NMR ($CDCl_3$, 300 MHz, δ/ppm) 0.93 (d, J = 6.8 Hz, 3H, **H**$_3$C-CH-), 1.19 (d, J = 6.6 Hz, 3H, **H**$_3$C-CH-), 2.89-3.04 (m, 2H, 2 × -C**H**-CH$_3$), 4.22 (d, J = 9.6 Hz, 1H, -C**H**-Ar), 4.31 (d, J = 9.3 Hz, 1H, -C**H**-Ar), 4.85-4.92 (m, 2H, **H**$_2$C=CH-), 5.10-5.16 (m, 2H, **H**$_2$C=CH-), 5.45-5.57 (m, 1H, -C**H**=CH$_2$), 5.78-5.90 (m, 1H, -C**H**=CH$_2$), 6.10 (s, 2H, -O-C**H**$_2$-O-), 6.12 (s, 2H, -O-C**H**$_2$-O-), 6.68 (s, 1H, **H**-Ar),, 6.73 (s, 1H, **H**-Ar), 7.41 (s, 1H, **H**-Ar), 7.43 (s, 1H, **H**-Ar), 9.79 (s, 1H, -C**H**O), 9.81 (s, 1H, -C**H**O); ^{13}C NMR ($CDCl_3$, 75 MHz, δ/ppm) 18.71, 19.01, 38.95, 39.57, 57.97, 58.44, 103.02, 105.73, 105.83, 109.74, 109.99, 115.72, 116.44, 127.05, 139.74, 140.07, 147.02, 151.54, 198.85, 199.57; Anal. calcd for $C_{13}H_{13}NO_5$ C, 59.31; H, 4.98; N, 5.32; found C, 59.25; H, 4.94; N, 5.36.

Data for compound 3e. Thick viscous liquid, (85%), diastereomeric ratio = 1:1.16

3-Methyl-2-(2-nitrophenyl)pent-4-enal (3e).

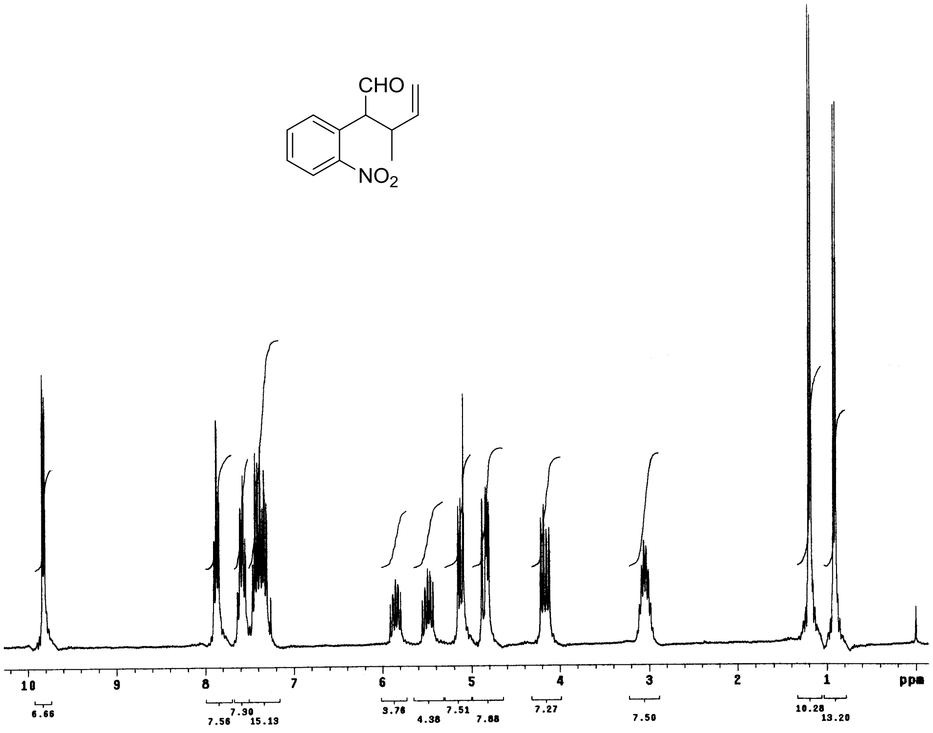

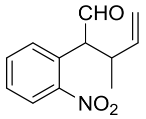

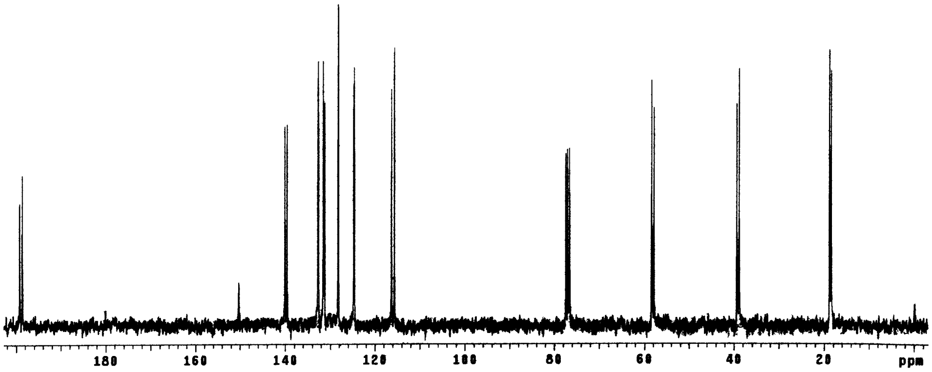

IR ($CHCl_3$) ν/cm^{-1} 2970.2, 2727.2, 1726.2, 1527.5, 1352.0; ^{1}H NMR ($CDCl_3$, 300 MHz, δ/ppm) 0.91 (d, *J* = 6.8 Hz, 3H, **H**$_3$C-CH-), 1.19 (d, *J* = 6.6 Hz, 3H, **H**$_3$C-CH-), 2.96-3.08 (m, 2H, 2 × -C**H**-CH$_3$), 4.11-4.21 (m, 2H, 2 × -C**H**-Ar), 4.80-4.88 (m, 2H, **H**$_2$C=CH-), 5.09-5.15 (m, 2H, **H**$_2$C=CH-), 5.43-5.55 (m, 1H, -C**H**=CH$_2$), 5.79-5.91 (m, 1H, -C**H**=CH$_2$), 7.27-7.46 (m, 4H, 4 × **H**-Ar), 7.54-7.63 (m, 2H, 2 × **H**-Ar), 7.84-7.90 (m, 2H, 2 × **H**-Ar), 9.81 (d, *J* = 1.1 Hz, 1H, -C**H**O), 9.83 (d, *J* = 1.3 Hz, 1H, -C**H**O); ^{13}C NMR ($CDCl_3$, 75 MHz, δ/ppm) 18.45, 18.83, 38.86, 39.37, 57.91, 58.42, 115.65 116.29, 124.61, 124.73, 128.15, 128.20, 131.24, 131.51, 132.60, 132.71, 139.55, 140.02, 150.25, 198.64, 199.24; Anal. calcd for $C_{12}H_{13}NO_3$ C, 65.74; H, 5.98; N, 6.39; found C, 65.68; H, 5.94; N, 6.45.

Data for compound 3f. Thick viscous liquid, (83%), diastereomeric ratio = 1:9.01

2-(2-Bromo-3,4,5-trimethoxy-6-nitrophenyl)-3-methylpent-4-enal (3f).

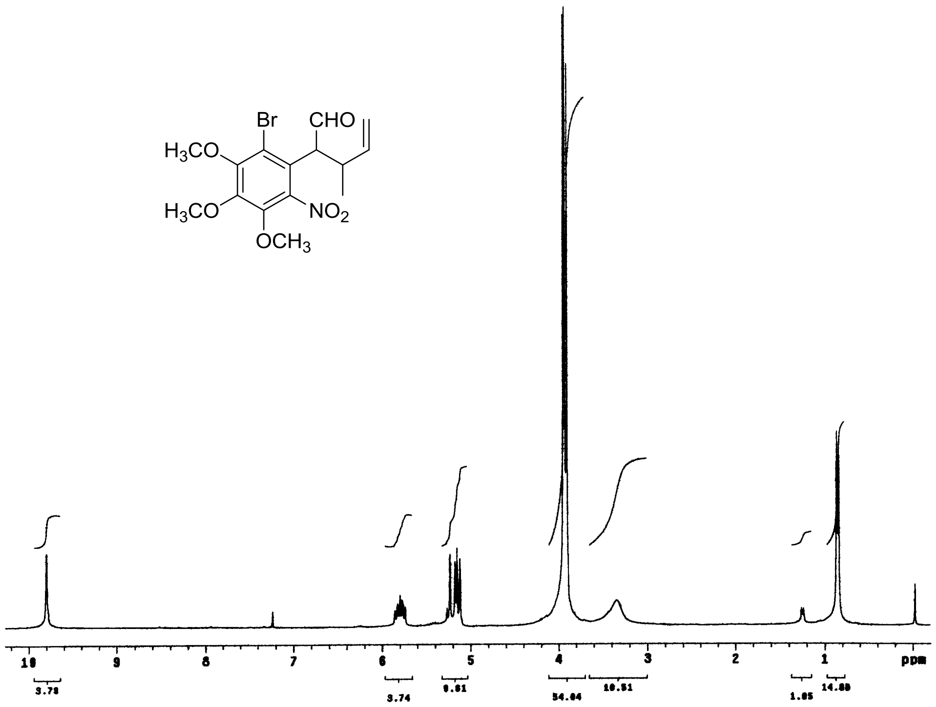

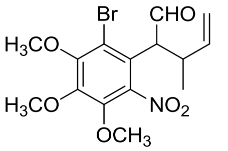

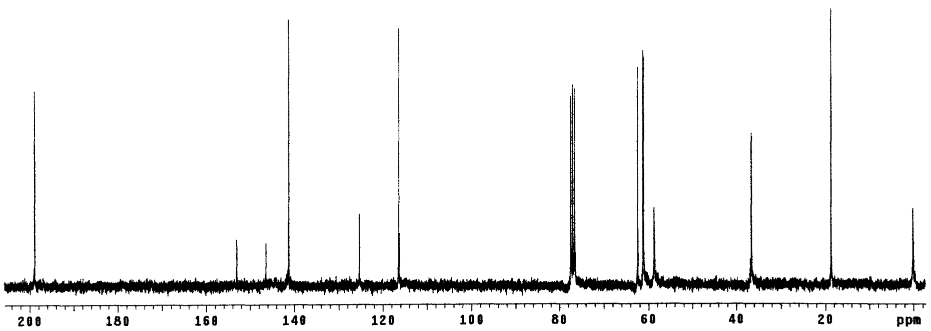

IR ($CHCl_3$) ν/cm^{-1} 2843.1, 1724.4, 1537.3, 1365.6, 1236.4; ^{1}H NMR ($CDCl_3$, 300 MHz, δ/ppm) 0.85 (d, *J* = 6.6 Hz, 3H, **H**$_3$C-CH-), 1.25 (d, *J* = 6.3 Hz, 3H, **H**$_3$C-CH-), 3.34-3.44 (m, 4H, 2 ×-C**H**-CH$_3$ and 2 × -C**H**-Ar), 3.90 (s, 3H, **H**$_3$CO-Ar), 3.92 (s, 3H, **H**$_3$CO-Ar), 3.93 (s, 6H, 2 × **H**$_3$CO-Ar), 3.94 (s, 6H, 2 × **H**$_3$CO-Ar), 5.11-5.26 (m, 4H, 2 × **H**$_2$C=CH-), 5.73-5.85 (m, 2H, 2 × -C**H**=CH$_2$), 9.80 (s, 2H, 2 × -C**H**O); ^{13}C NMR ($CDCl_3$, 75 MHz, δ/ppm) 18.72, 36.59, 58.72, 61.16, 61.25, 62.43, 116.36, 125.34, 141.24, 146.39, 153.04, 198.87; Anal. calcd for $C_{15}H_{18}BrNO_6$ C, 46.41; H, 4.67; N, 3.61; found C, 46.39; H, 4.76; N, 3.67.

Data for compound 3g. Thick viscous liquid, (83%), diastereomeric ratio = 1:2.42

2-(5-Methoxy-2-nitrophenyl)-3-methylpent-4-enal (3g).

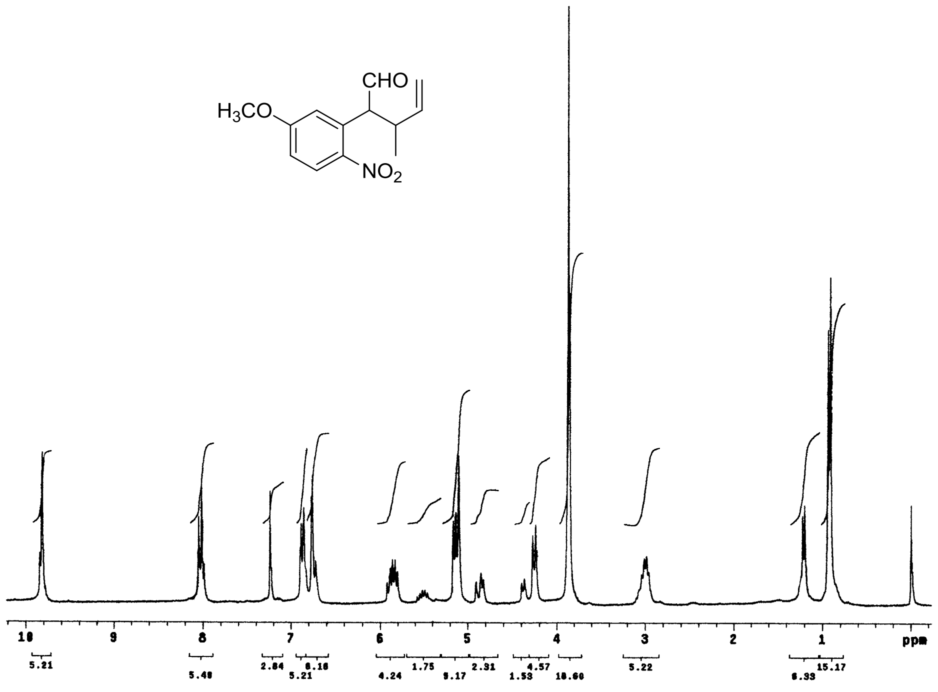

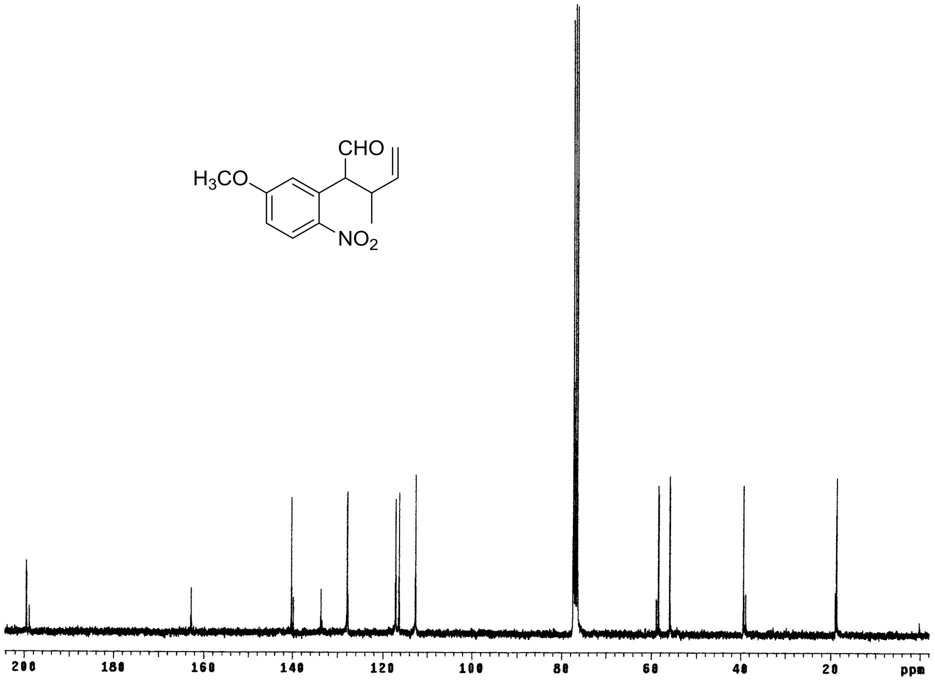

IR ($CHCl_3$) ν/cm^{-1} 2924.1, 1724.4, 1583.6, 1516.1, 1340.5, 1269.2, 1078.2; ^{1}H NMR ($CDCl_3$, 300 MHz, δ/ppm) 0.93 (d, *J* = 6.8 Hz, 3H, **H**$_3$C-CH-), 1.21 (d, *J* = 6.6 Hz, 3H, **H**$_3$C-CH-), 2.96-3.04 (m, 2H, 2 × -C**H**-CH_3), 3.86 (s, 3H, **H**$_3$CO-Ar), 3.88 (s, 3H, **H**$_3$CO-Ar), 4.26 (d, *J* = 9.3 Hz, 1H, -C**H**-Ar), 4.38 (d, *J* = 8.8 Hz, 1H, -C**H**-Ar), 4.82-4.90 (m, 2H, **H**$_2$C=CH-), 5.11-5.17 (m, 2H, **H**$_2$C=CH-), 5.46-5.54 (m, 1H, -C**H**=CH_2), 5.80-5.92 (m, 1H, -C**H**=CH_2), 6.75 (d, *J* = 12.6 Hz, 2H, 2 × **H**-Ar), 6.87 (d, *J* = 8.8 Hz, 2H, 2 × **H**-Ar), 7.99-8.09 (m, 2H, 2 × **H**-Ar), 9.82 (s, 1H, -C**H**O), 9.84 (s, 1H, -C**H**O); ^{13}C NMR ($CDCl_3$, 75 MHz, δ/ppm) 18.76, 19.01, 38.87, 39.38, 55.95, 58.43, 58.77, 112.64, 112.71, 116.37, 117.15, 127.75, 127.91, 133.65, 139.85, 140.26, 162.82, 198.89, 199.52; Anal. calcd for $C_{13}H_{15}NO_4$ C, 62.64; H, 6.07; N, 5.62; found C, 62.60; H, 6.16; N, 5.61.

Data for compound 3h. Thick viscous liquid, (85%), diastereomeric ratio = 1:1.87

2-(2-Chloro-6-nitrophenyl)-3-methylpent-4-enal (3h).

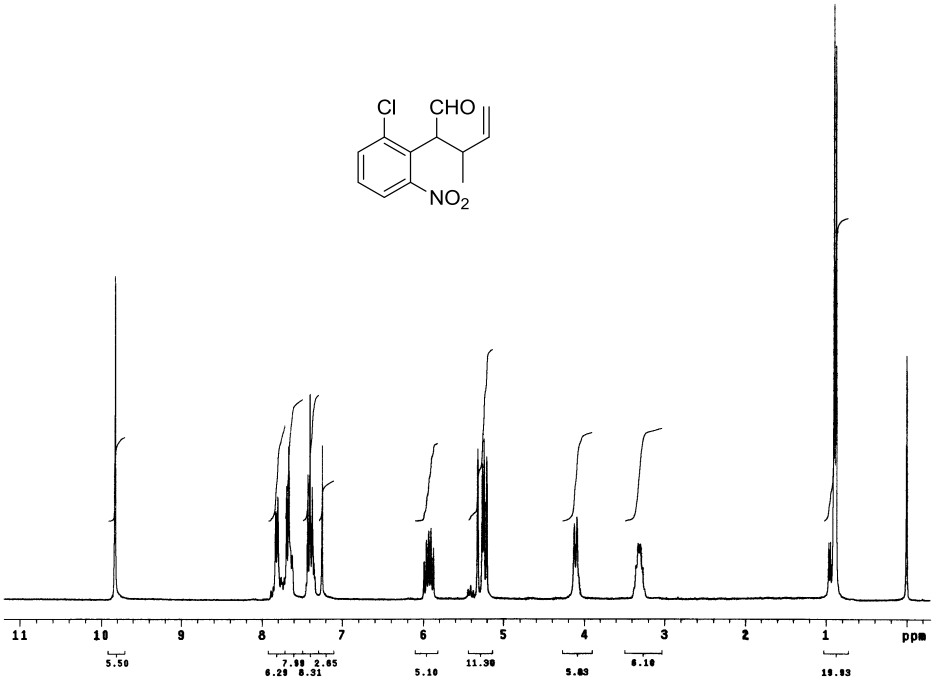

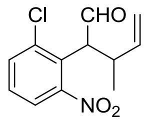

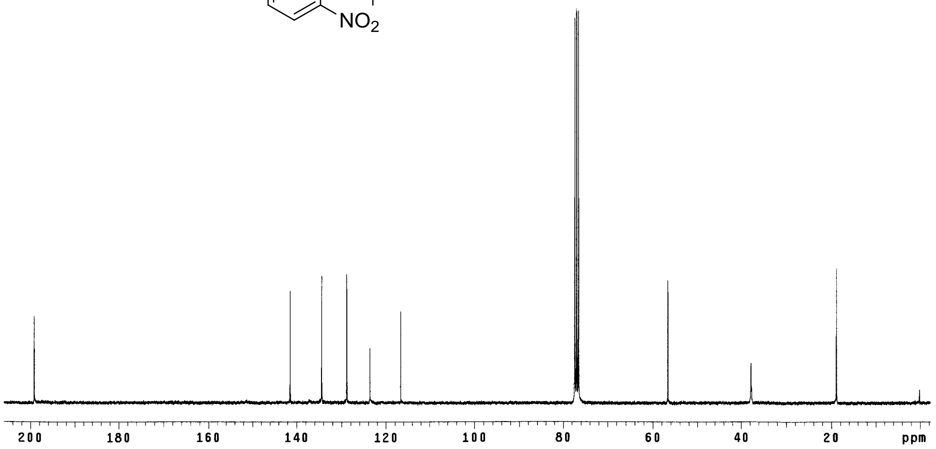

IR ($CHCl_3$) ν/cm^{-1} 2970.4, 2739.0, 1720.5, 1639.5, 1531.5, 1356.0; ^{1}H NMR ($CDCl_3$, 300 MHz, δ/ppm) 0.88 (d, *J* = 6.8 Hz, 3H, **H**$_3$C-CH-), 0.95 (d, *J* = 6.0 Hz, 3H, **H**$_3$C-CH-), 3.26-3.35 (m, 2H, 2 × -C**H**-CH_3), 4.10 (d, *J* = 10.1 Hz, 2H, 2 × -C**H**-Ar), 5.20-5.46 (m, 4H, 2 × **H**$_2$C=CH-), 5.87-5.99 (m, 2H, 2 × -C**H**=CH_2), 7.33-7.44 (m, 2H, 2 × **H**-Ar), 7.62-7.70 (m, 2H, 2 × **H**-Ar), 7.72-7.86 (m, 2H, 2 × **H**-Ar), 9.82 (s, 2H, 2 × -C**H**O); ^{13}C NMR ($CDCl_3$, 75 MHz, δ/ppm) 18.87, 37.77, 56.69, 116.67, 123.68, 128.86, 134.42, 141.47, 199.19; Anal. calcd for $C_{12}H_{12}ClNO_3$ C, 56.81; H, 4.77; N, 5.52; found C, 56.75; H, 4.86; N, 5.43.

Data for compound 4a. Thick viscous liquid, (92%), diastereomeric ratio = 1:1.5

2-(1-(3-Chloro-4,5-dimethoxy-2-nitrophenyl)-2-methylbut-3-en-1-yl)-1,3-dioxolane (4a).

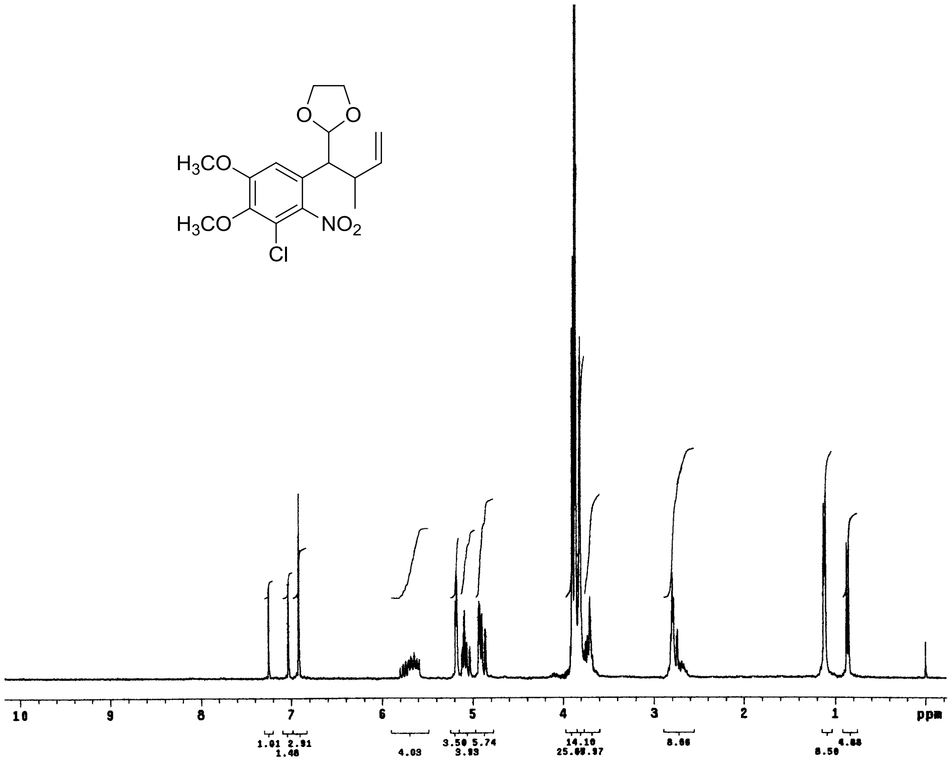

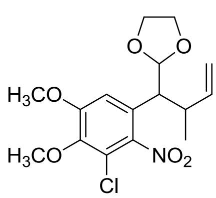

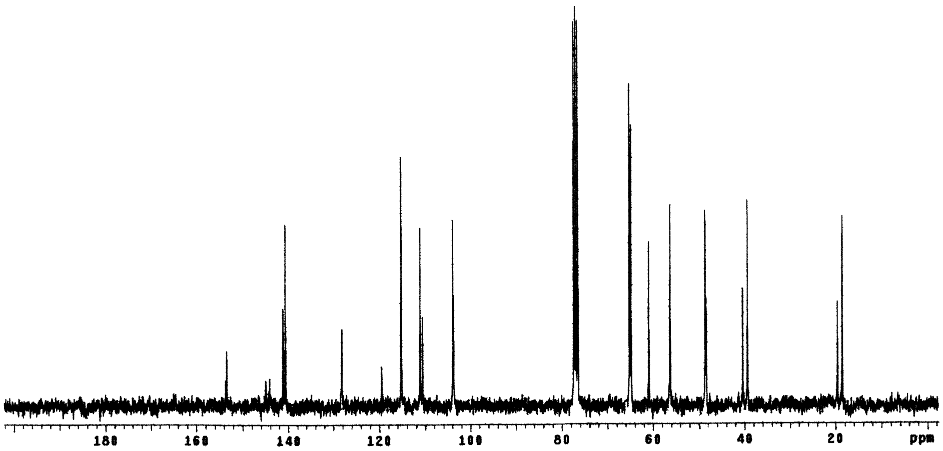

IR ($CHCl_3$) ν/cm^{-1} 2929.9, 1637.6, 1533.4, 1361.7, 1217.1, 1080.1; ^{1}H NMR ($CDCl_3$, 300 MHz, δ/ppm) 0.87 (d, *J* = 6.6 Hz, 3H, **H**$_3$C-CH-), 1.13 (d, *J* = 7.1 Hz, 3H, **H**$_3$C-CH-), 2.69-2.83 (m, 2H, 2 × -C**H**-CH_3), 3.67-3.93 (m, 22H, 4 × Ar-OC**H**$_3$, 2 × -C**H**$_2$-C**H**$_2$-O- and 2 × -C**H**-Ar), 4.86-5.11 (m, 4H, 2 × **H**$_2$C=CH-), 5.18 (d, 2H, *J* = 4.1 Hz, 2 × -O-C**H**-O-), 5.61-5.80 (m, 2H, 2 × -C**H**=CH_2), 6.95 (s, 1H, **H**-Ar), 7.06 (s, 1H, **H**-Ar); ^{13}C NMR ($CDCl_3$, 75 MHz, δ/ppm) 18.54, 19.57, 39.39, 40.44, 48.38, 48.60, 56.23, 56.33, 60.92, 64.84, 65.19, 103.75, 103.90, 110.61, 111.13, 115.23, 119.49, 128.15, 140.68, 141.17, 144.15, 144.99, 153.45, 153.70; Anal. calcd for $C_{16}H_{20}ClNO_6$ C, 53.71; H, 5.63; N, 3.91; found C, 53.65; H, 5.72; N, 3.87.

Data for compound 4b. Thick viscous liquid, (91%), diastereomeric ratio = 1:1.12

2-(1-(3-Bromo-4,5-dimethoxy-2-nitrophenyl)-2-methylbut-3-en-1-yl)-1,3-dioxolane (4b).

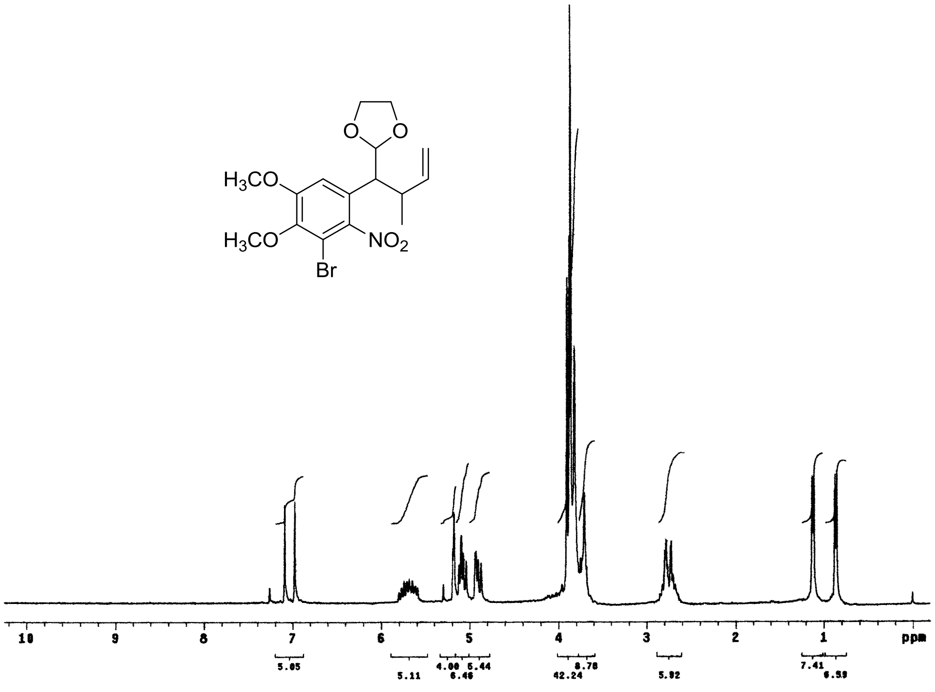

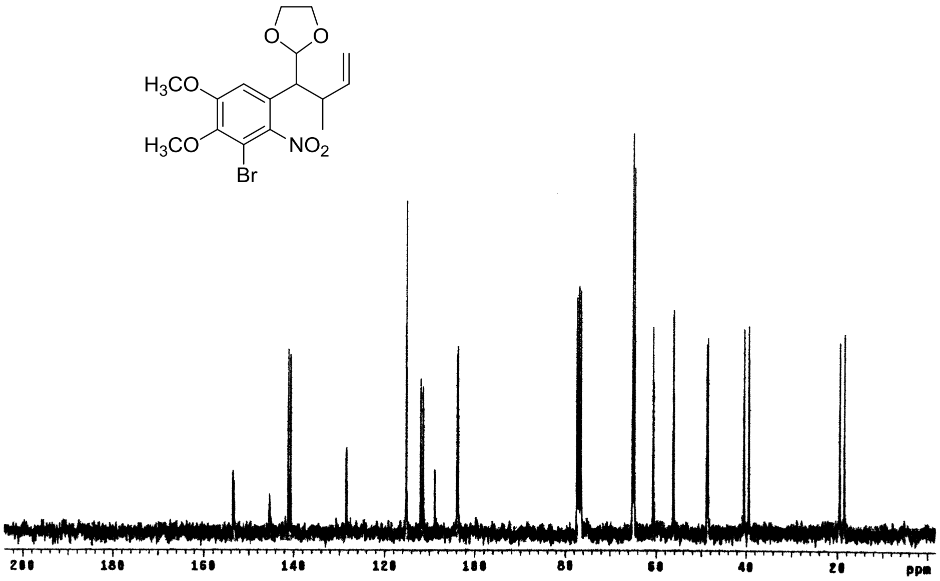

IR ($CHCl_3$) ν/cm^{-1} 2939.3, 1641.3, 1533.3, 1367.4, 1209.2, 1076.2; ^1H NMR ($CDCl_3$, 300 MHz, δ/ppm) 0.86 (d, J = 6.0 Hz, 3H, **H**$_3$C-CH-), 1.12 (d, J = 5.7 Hz, 3H, **H**$_3$C-CH-), 2.58-2.84 (m, 2H, 2 × -C**H**-CH_3), 3.68-3.91 (m, 22H, 4 × **H**$_3$CO-Ar, 2 × -C**H**$_2$-C**H**$_2$-O- and 2 × -C**H**-Ar), 4.87-5.12 (m, 4H, 2 × **H**$_2$C=CH-), 5.18 (d, J = 3.8 Hz, 1H, -O-C**H**-O-), 5.29 (d, J = 1.8 Hz, 1H, -O-C**H**-O-), 5.62-5.78 (m, 2H, 2 × -C**H**=CH_2), 6.94 (s, 1H, **H**-Ar), 7.06 (s, 1H, **H**-Ar); ^{13}C NMR ($CDCl_3$, 75 MHz, δ/ppm) 18.51, 19.55, 39.42, 40.48, 48.60, 48.82, 56.22, 56.32, 60.73, 64.81, 65.18, 103.75, 103.92, 108.83, 111.45, 111.98, 115.20, 128.40, 128.52, 140.66, 141.15, 145.26, 153.27, 153.53; Anal. calcd for $C_{16}H_{20}BrNO_6$ C, 47.78; H, 5.01; N, 3.48; found C, 47.70; H, 5.10; N, 3.40.

Data for compound 4c. Thick viscous liquid, (90%), diastereomeric ratio = 1:1.24

2-(2-Methyl-1-(3,4,5-trimethoxy-2-nitrophenyl)but-3-en-1-yl)-1,3-dioxolane (4c).

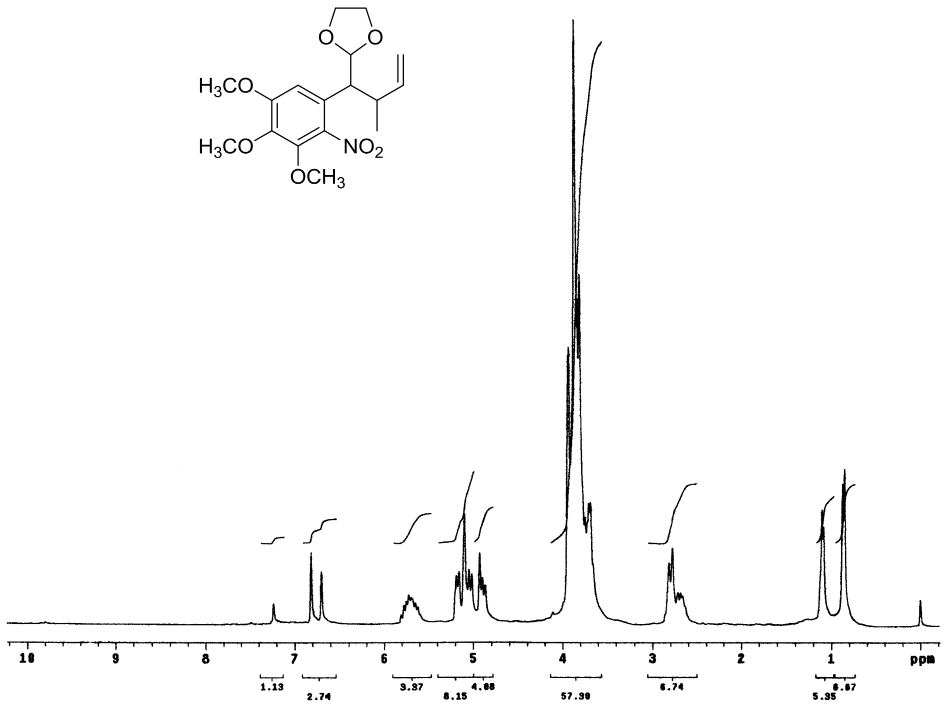

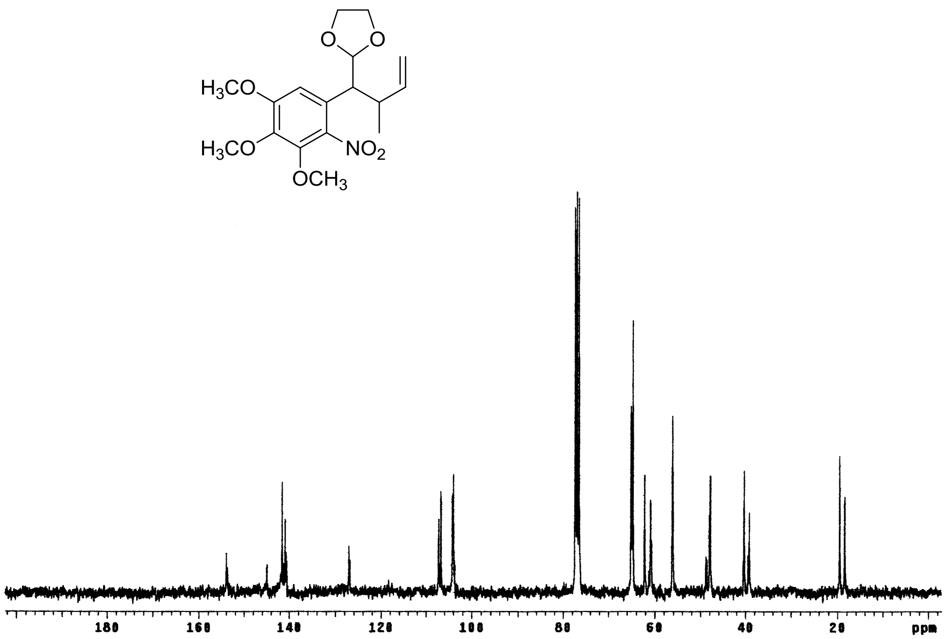

IR ($CHCl_3$) ν/cm^{-1} 2966.6, 1637.6, 1531.5, 1367.5, 1246.0, 1026.1; ^{1}H NMR ($CDCl_3$, 300 MHz, δ/ppm) 0.86 (d, *J* = 6.3 Hz, 3H, **H**$_3$C-CH-), 1.10 (d, *J* = 5.2 Hz, 3H, **H**$_3$C-CH-), 2.66-2.82 (m, 4H, 2 × -C**H**-CH$_3$ and 2 × -C**H**-Ar), 3.66-3.95 (m, 26H, 6 × **H**$_3$CO-Ar and 2 × -C**H**$_2$-C**H**$_2$-O-), 4.87-4.93 (m, 2H, 2 × -O-C**H**-O-), 5.01-5.19 (m, 4H, 2 × **H**$_2$C=CH-), 5.62-5.77 (m, 2H, 2 × -C**H**=CH$_2$), 6.70 (s, 1H, **H**-Ar), 6.82 (s, 1H, **H**-Ar); ^{13}C NMR ($CDCl_3$, 75 MHz, δ/ppm) 18.49, 19.60, 39.20, 39.45, 47.92, 48.07, 56.13, 56.23, 60.76, 60.97, 61.26, 62.24, 64.83, 65.18, 104.05, 104.22, 106.77, 107.25, 126.81, 127.08, 140.70, 140.92, 141.20, 141.58, 144.94, 153.74, 153.99; Anal. calcd for $C_{17}H_{23}NO_7$ C, 57.78; H, 6.56; N, 3.96; found C, 57.70; H, 6.61; N, 3.91.

Data for compound 4d. Thick viscous liquid, (92%), diastereomeric ratio = 1:1.66

5-(1-(1,3-Dioxolan-2-yl)-2-methylbut-3-en-1-yl)-6-nitrobenzo[*d*][1,3]dioxole (4d).

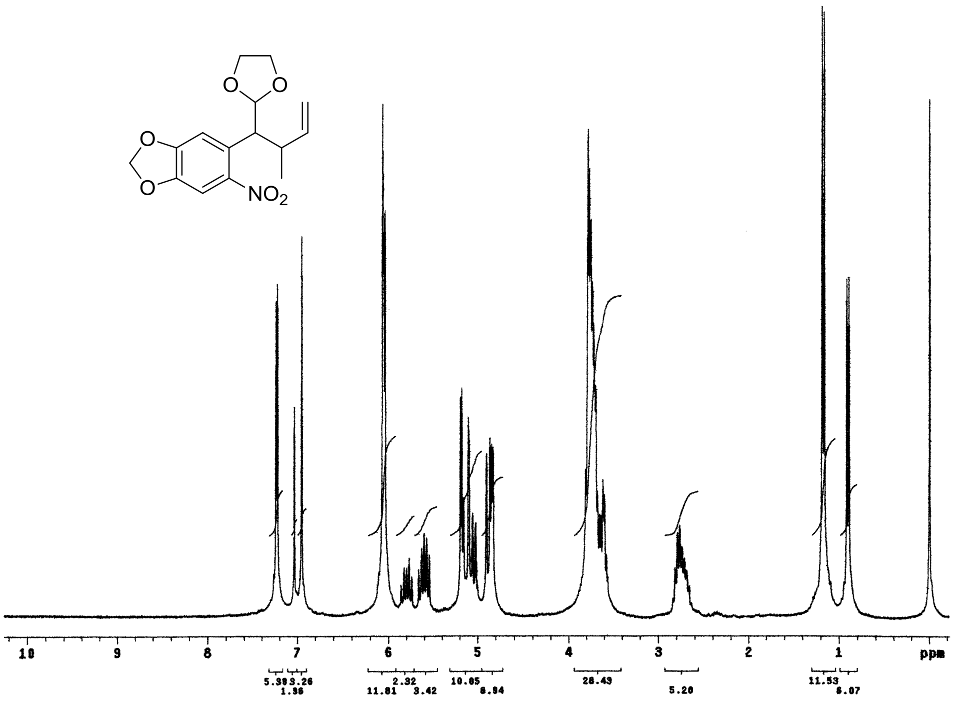

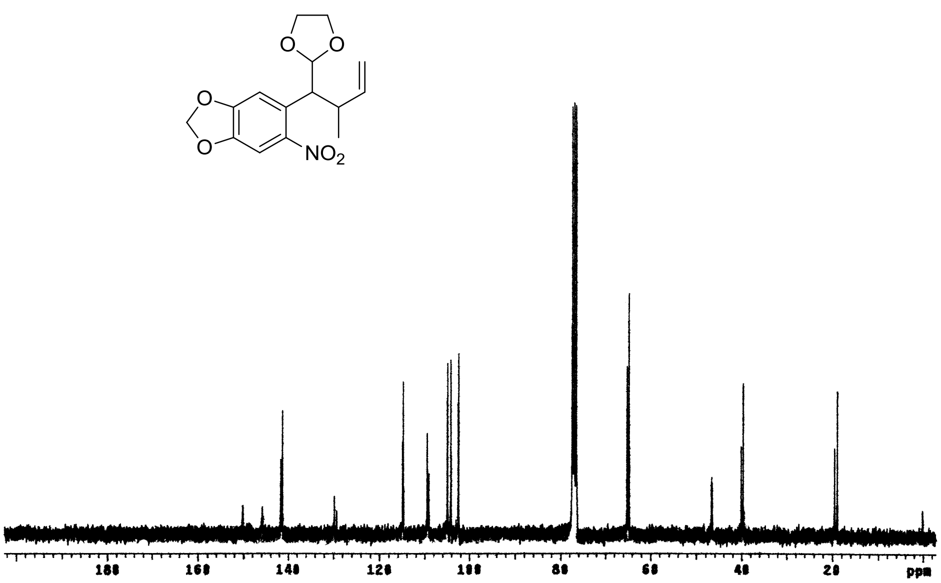

IR ($CHCl_3$) ν/cm^{-1} 2920.3, 1620.2, 1525.7, 1342.5, 1253.7, 1035.8; ^{1}H NMR ($CDCl_3$, 300 MHz, δ/ppm) 0.90 (d, *J* = 6.8 Hz, 3H, **H**$_3$C-CH-), 1.17 (d, *J* = 6.6 Hz, 3H, **H**$_3$C-CH-), 2.66-2.81 (m, 2H, 2 × -C**H**-CH_3), 3.56-3.80 (m, 10H, 2 × -C**H**$_2$-C**H**$_2$-O- and 2 × -C**H**-Ar), 4.83-4.90 (m, 2H, 2 × -O-C**H**-O-), 5.00-5.19 (m, 4H, 2 × **H**$_2$C=CH-), 5.54-5.66 (m, 1H, -C**H**=CH_2), 5.74-5.96 (m, 1H, -C**H**=CH_2), 6.03 (s, 2H, -O-C**H**$_2$-O-), 6.05 (s, 2H, -O-C**H**$_2$-O-), 6.95 (s, 1H, **H**-Ar), 7.03 (s, 1H, **H**-Ar), 7.22 (s, 1H, **H**-Ar), 7.24 (s, 1H, **H**-Ar); ^{13}C NMR ($CDCl_3$, 75 MHz, δ/ppm) 18.93, 19.53, 39.69, 40.11, 46.75, 64.77, 65.12, 102.46, 104.16, 104.93, 109.11, 109.45, 114.83, 114.97, 129.27, 129.79, 141.32, 141.68, 145.81, 150.15; Anal. calcd for $C_{15}H_{17}NO_6$ C, 58.63; H, 5.58; N, 4.56; found C, 58.58; H, 5.67 N, 4.51.

Data for compound 4e. Thick viscous liquid, (91%), diastereomeric ratio = 1:2.63

2-(2-Methyl-1-(2-nitrophenyl)but-3-en-1-yl)-1,3-dioxolane (4e).

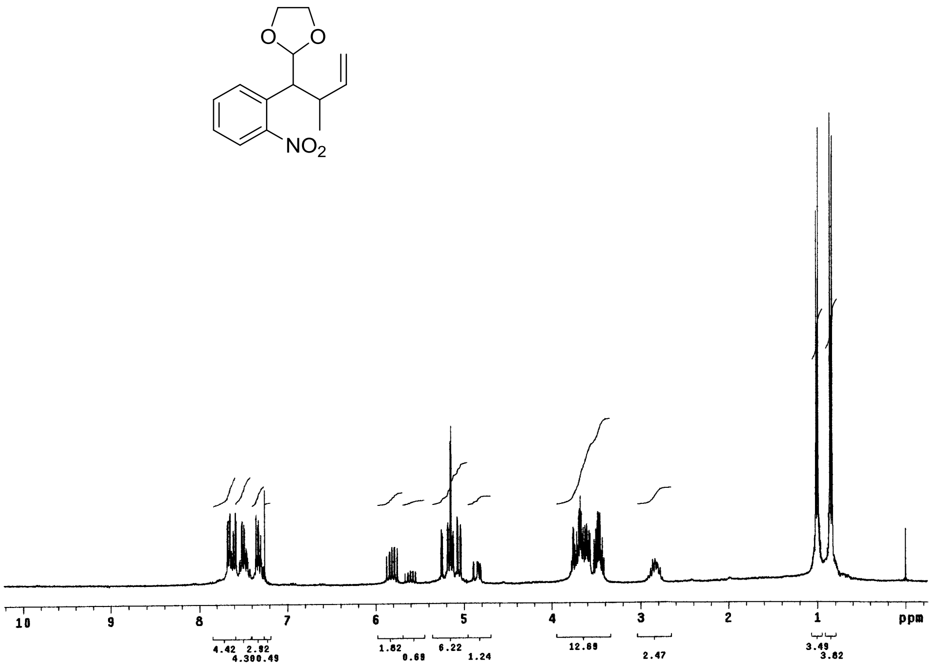

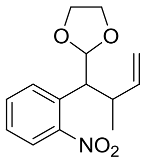

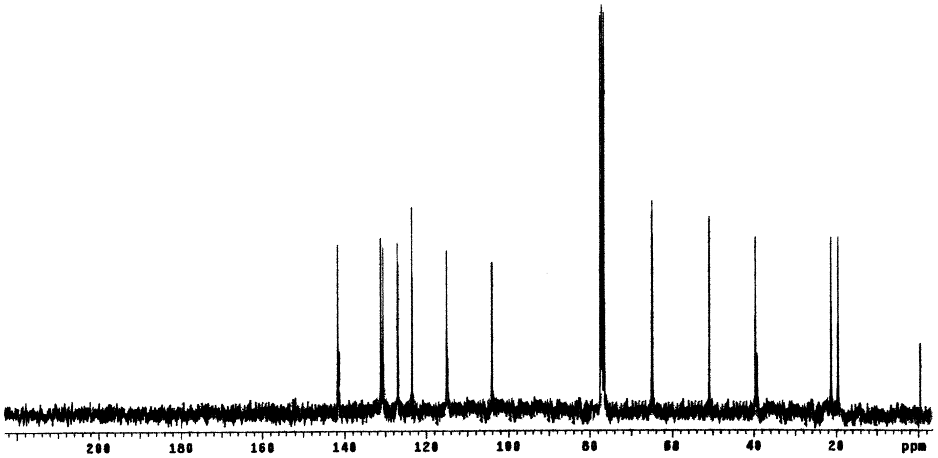

IR ($CHCl_3$) ν/cm^{-1} 3076.2, 1641.3, 1529.4, 1359.7, 1139.9; ^{1}H NMR ($CDCl_3$, 300 MHz, δ/ppm) 0.90 (d, *J* = 6.3 Hz, 3H, **H**$_3$C-CH-), 1.12 (d, *J* = 5.8 Hz, 3H, **H**$_3$C-CH-), 2.67-2.85 (m, 2H, 2 × -C**H**-CH_3), 3.41-3.79 (m, 10H, 2 × **-CH$_2$-CH$_2$-O-** and 2 × -C**H**-Ar), 5.03-5.25 (m, 6H, 2 × **H**$_2$C=CH- and 2 × **-O-CH-O-**), 5.54-5.66 (m, 1H, -C**H**=CH_2), 5.75-5.87 (m, 1H, -C**H**=CH_2), 7.26-7.37 (m, 2H, 2 × **H**-Ar), 7.42-7.54 (m, 2H, 2 × **H**-Ar), 7.58-7.69 (m, 4H, 4 × **H**-Ar); ^{13}C NMR ($CDCl_3$, 75 MHz, δ/ppm) 19.56, 21.23, 39.23, 39.60, 50.88, 64.87, 64.97, 104.04, 114.79, 115.03, 123.47, 126.96, 130.52, 130.89, 131.11, 132.41, 141.31, 141.67; Anal. calcd for $C_{14}H_{17}NO_4$ C, 63.87; H, 6.51; N, 5.32; found C, 63.96; H, 6.59; N, 5.25.

Data for compound 4f. Thick viscous liquid, (92%), diastereomeric ratio = 1:1

2-(1-(2-Bromo-3,4,5-trimethoxy-6-nitrophenyl)-2-methylbut-3-en-1-yl)-1,3-dioxolane (4f).

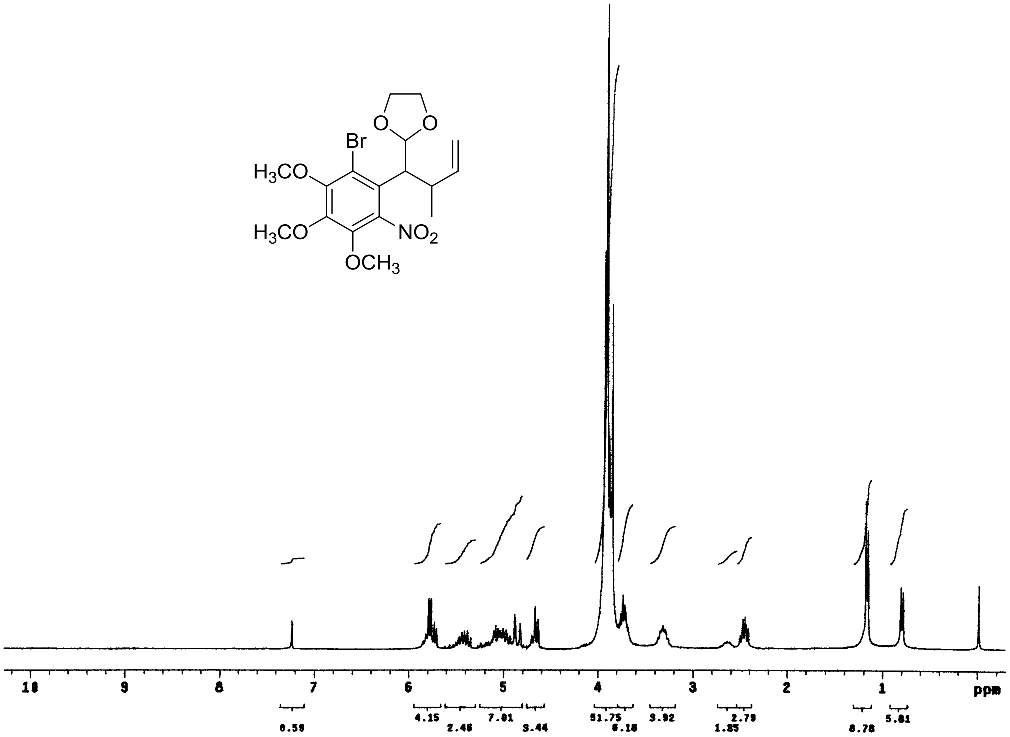

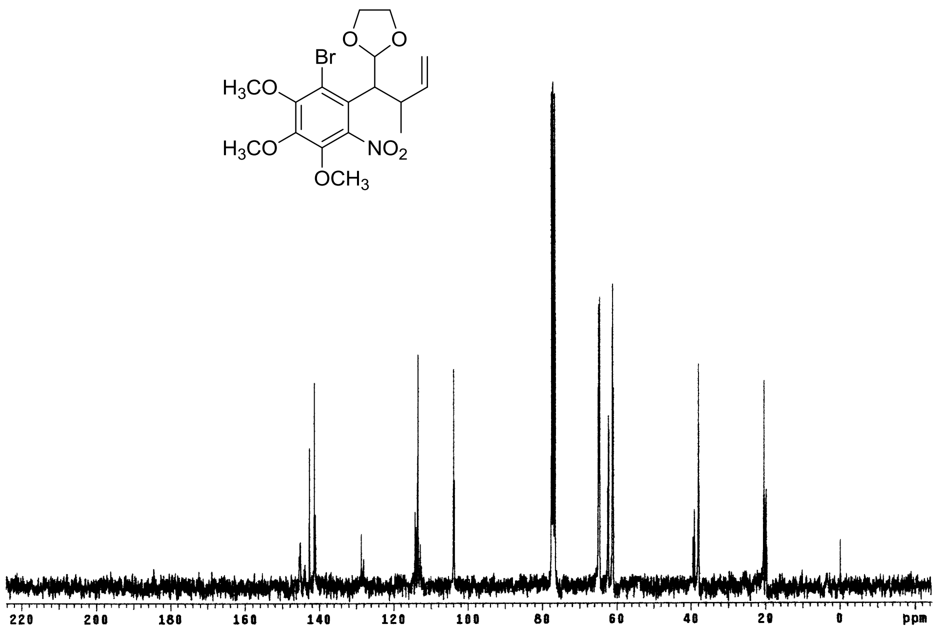

IR ($CHCl_3$) ν/cm^{-1} 2891.3, 1641.4, 1537.3, 1367.5, 1230.6, 1024.2; ^{1}H NMR ($CDCl_3$, 300 MHz, δ/ppm) 0.79 (d, *J* = 6.8 Hz, 3H, **H**$_3$C-CH-), 1.16 (d, *J* = 6.6 Hz, 3H, **H**$_3$C-CH-), 2.41-2.66 (m, 2H, 2 × -C**H**-CH_3), 3.25-3.36 (m, 2H, 2 × -C**H**-Ar), 3.59-4.08 (m, 26H, 6 × **H**$_3$CO-Ar and 2 × -O-C**H**$_2$-C**H**$_2$-O-), 4.62-4.70 (m, 2H, 2 × -O-C**H**-O-), 4.82-5.50 (m, 4H, 2 × **H**$_2$C=CH-), 5.70-5.84 (m, 2H, 2 × -C**H**=CH_2); ^{13}C NMR ($CDCl_3$, 75 MHz, δ/ppm) 20.13, 20.47, 37.88, 38.02, 39.09, 39.41, 60.99, 61.08, 61.18, 62.26, 62.42, 64.66, 64.77, 64.86, 64.95, 103.80, 103.94, 112.90, 113.52, 113.61, 114.05, 128.61, 141.07, 141.35, 142.68, 145.17; Anal. calcd for $C_{17}H_{22}BrNO_7$ C, 47.24; H, 5.13; N, 3.24; found C, 47.19; H, 5.21; N, 3.28.

Data for compound 4g. Thick viscous liquid, (91%), diastereomeric ratio = 1:1.11

2-(1-(5-Methoxy-2-nitrophenyl)-2-methylbut-3-en-1-yl)-1,3-dioxolane (4g).

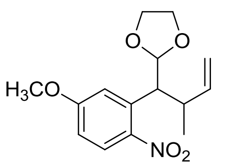

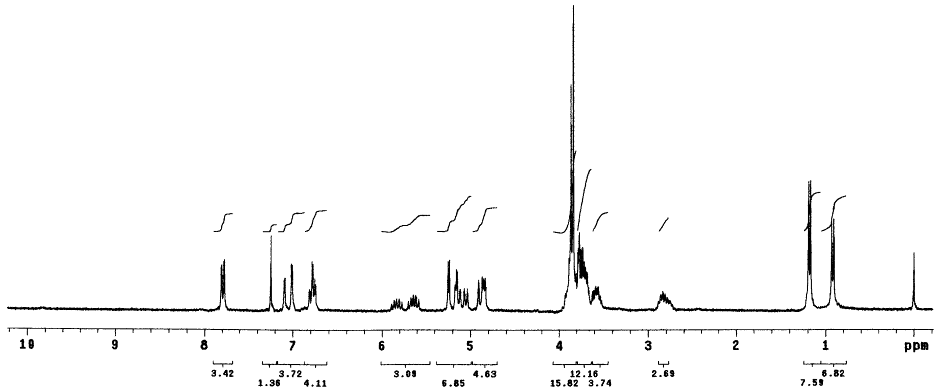

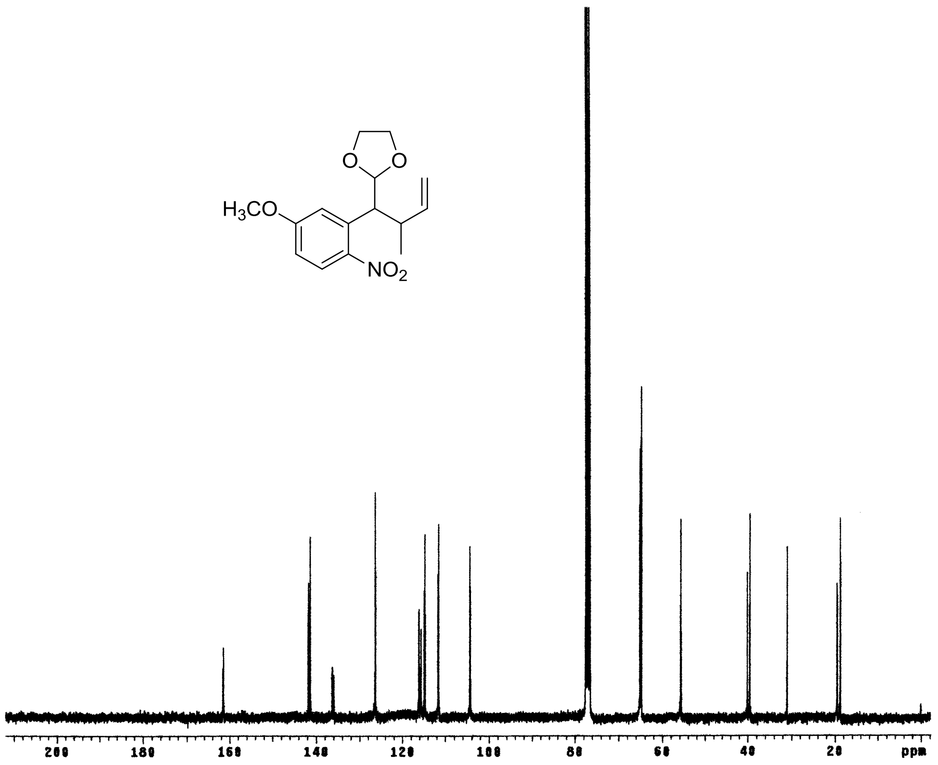

IR ($CHCl_3$) ν/cm^{-1} 2968.5, 1608.6, 1516.1, 1346.3, 1244.1, 1030.0; ^{1}H NMR ($CDCl_3$, 300 MHz, δ/ppm) 0.91 (d, J = 6.8 Hz, 3H, $\mathbf{H}_3$C-CH-), 1.17 (d, J = 7.0 Hz, 3H, $\mathbf{H}_3$C-CH-), 2.75-2.85 (m, 2H, 2 × -C**H**-CH_3), 3.51-3.63 (m, 2H, 2 × -C**H**-Ar), 3.66-3.82 (m, 8H, 2 × -C$\mathbf{H}_2$-C$\mathbf{H}_2$-O-), 3.85 (s, 3H, $\mathbf{H}_3$CO-Ar), 3.86 (s, 3H, $\mathbf{H}_3$CO-Ar), 4.83-4.90 (m, 2H, 2 × -O-C**H**-O-), 5.03-5.24 (m, 4H, 2 × $\mathbf{H}_2$C=CH-), 5.58-5.78 (m, 2H, 2 × -C**H**=CH_2), 6.74-6.81 (m, 2H, 2 × **H**-Ar), 7.01 (d, J = 2.7 Hz, 1H, **H**-Ar), 7.09 (d, J = 2.7 Hz, 1H, **H**-Ar), 7.77 (d, J = 1.9 Hz, 1H, **H**-Ar), 7.80 (d, J = 1.6 Hz, 1H, **H**-Ar); ^{13}C NMR ($CDCl_3$, 75 MHz, δ/ppm) 18.78, 19.53, 30.99, 39.55, 40.11, 55.68, 55.74, 64.81, 65.11, 104.30, 104.39, 111.58, 111.70, 114.73, 114.87, 115.68, 116.15, 126.36, 136.01, 136.33, 141.37, 141.77, 161.50, 161.62; Anal. calcd for $C_{15}H_{19}NO_5$ C, 61.42; H, 6.53; N, 4.78; found C, 61.35; H, 6.59; N, 4.83.

Data for compound 4h. Thick viscous liquid, (90%), diastereomeric ratio = 1:1.86

2-(1-(2-Chloro-6-nitrophenyl)-2-methylbut-3-en-1-yl)-1,3-dioxolane (4h).

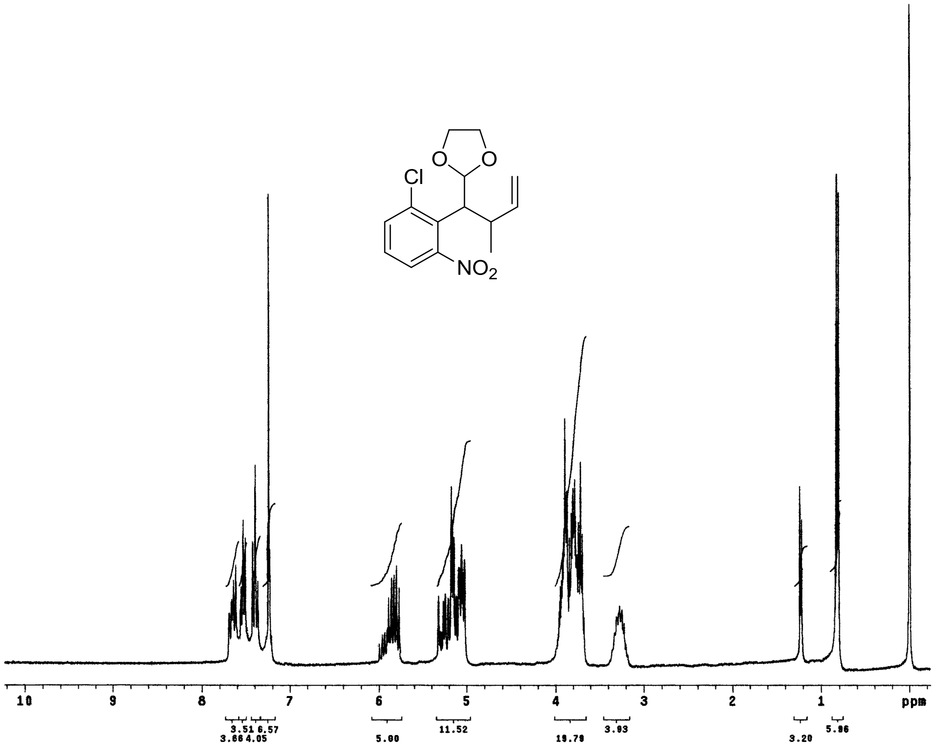

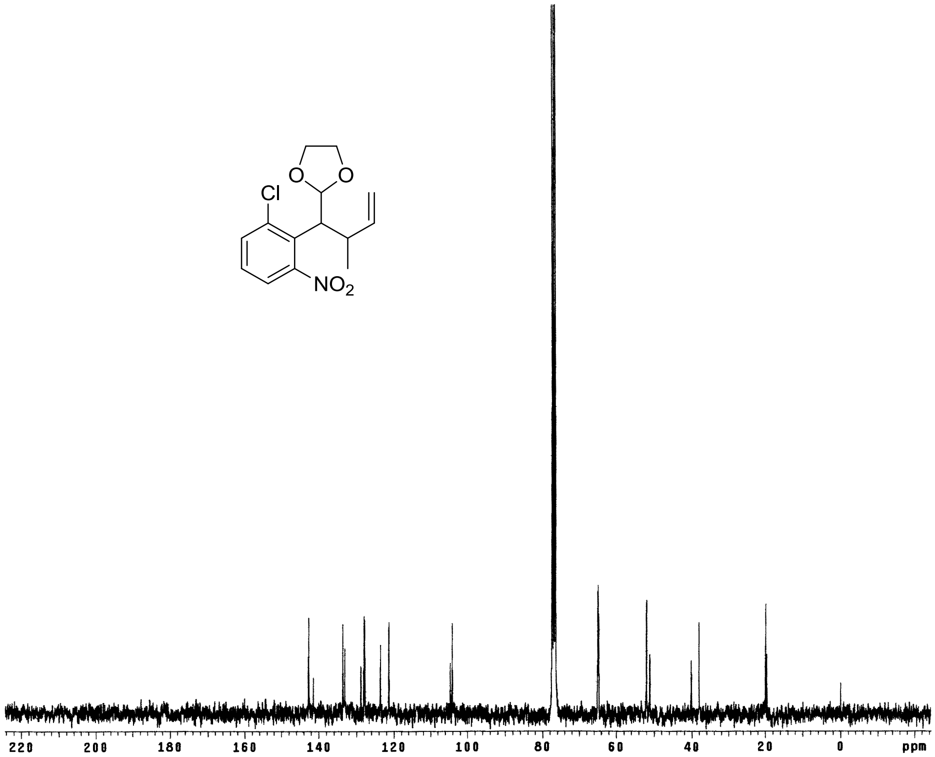

IR ($CHCl_3$) ν/cm^{-1} 2923.9, 1631.7, 1527.5, 1353.9, 1043.4; ^{1}H NMR ($CDCl_3$, 300 MHz, δ/ppm) 0.83 (d, J = 6.8 Hz, 3H, **H**$_3$C-CH-), 1.23 (d, J = 6.7 Hz, 3H, **H**$_3$C-CH-), 3.18-3.33 (m, 2H, 2 × -C**H**-CH$_3$), 3.67-3.98 (m, 10H, 2 × -C**H**$_2$-C**H**$_2$-O- and 2 × -C**H**-Ar), 5.02-5.38 (m, 6H, 2 × **H**$_2$C=CH- and 2 × -O-C**H**-O-), 5.80-5.99 (m, 2H, 2 × -C**H**=CH$_2$), 7.37-7.43 (m, 2H, 2 × **H**-Ar), 7.51-7.56 (m, 2H, 2 × **H**-Ar), 7.62-7.69 (m, 2H, 2 × **H**-Ar); ^{13}C NMR ($CDCl_3$, 75 MHz, δ/ppm) 19.60, 19.91, 38.07, 40.14, 51.11, 51.98, 64.68, 64.83, 64.90, 104.11, 104.64, 121.37, 123.64, 127.82, 128.03, 128.85, 133.11, 133.62, 141.47, 142.68; Anal. calcd for $C_{14}H_{16}NClO_4$ C, 56.48; H, 5.42; N, 4.70; found C, 56.50; H, 5.51; N, 4.65.

Data for compound 5a. Thick viscous liquid, (91%), diastereomeric ratio = 1:2.46

3-(3-Chloro-4,5-dimethoxy-2-nitrophenyl)-3-(1,3-dioxolan-2-yl)-2-methylpropanal (5a).

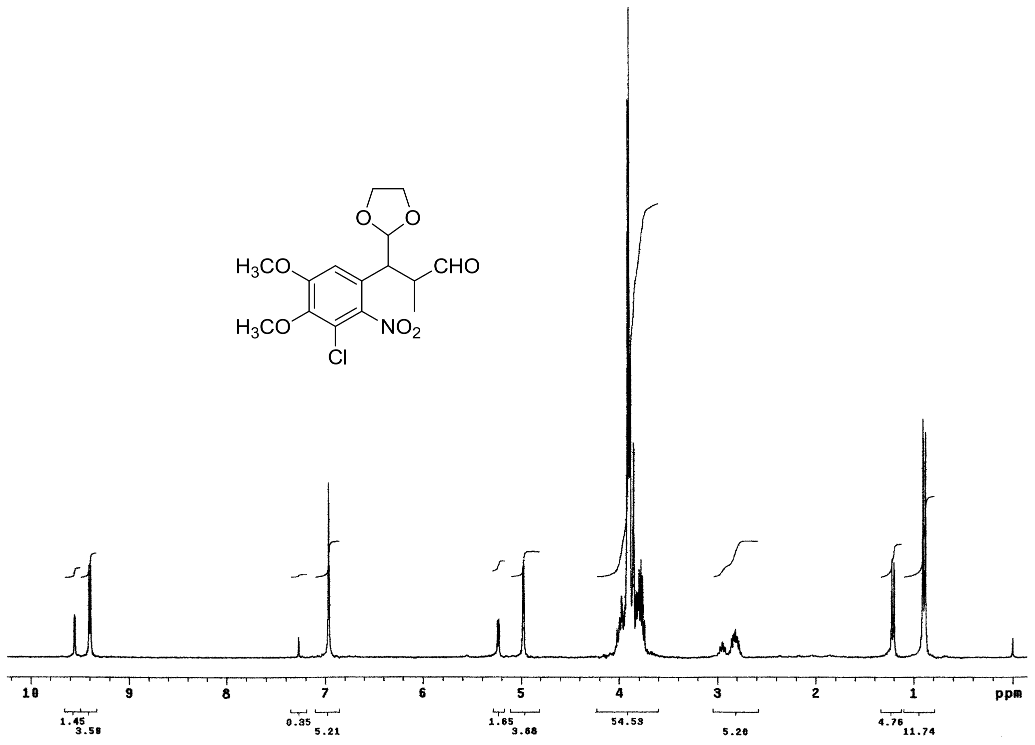

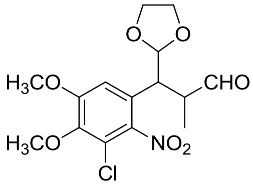

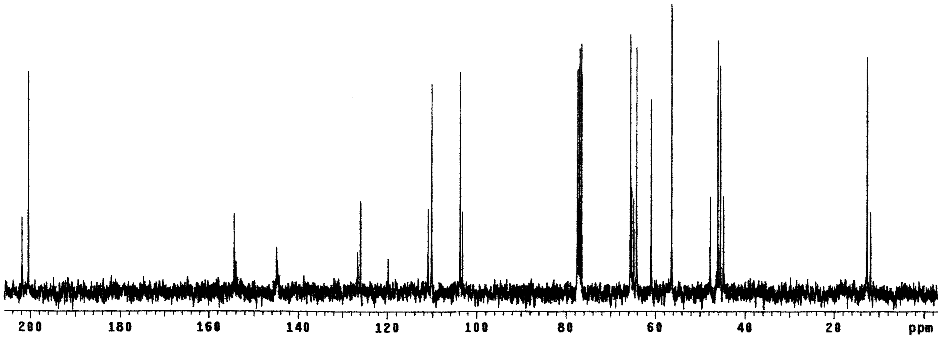

IR ($CHCl_3$) ν/cm^{-1} 2958.9, 2729.3, 1722.4, 1545.0, 1365.6, 1290.4, 1087.8; ^{1}H NMR ($CDCl_3$, 300 MHz, δ/ppm) 0.89 (d, *J* = 7.1 Hz, 3H, **H**$_3$C-CH-), 1.21 (d, *J* = 6.8 Hz, 3H, **H**$_3$C-CH-), 2.78-2.95 (m, 2H, 2 × -C**H**-CH_3), 3.74-4.01 (m, 22H, 4 × OC**H**$_3$-Ar, 2 × -C**H**$_2$-C**H**$_2$-O- and 2 × -C**H**-Ar), 4/97 (d, *J* = 2.7 Hz, 1H, -O-C**H**-O-), 5.23 (d, *J* = 4.1 Hz, 1H, -O-C**H**-O-), 6.96 (s, 2H, 2 × **H**-Ar), 9.40 (d, *J* = 4.6 Hz, 1H, -C**H**O), 9.50 (d, *J* = 2.2 Hz, 1H, -C**H**O); ^{13}C NMR ($CDCl_3$, 75 MHz, δ/ppm) 11.87, 12.68, 44.78, 45.52, 46.09, 47.76, 56.35, 60.95, 64.22, 64.85, 65.35, 65.60, 103.29, 103.80, 110.20, 110.99, 119.81, 126.00, 126.60, 144.95, 153.96, 154.35, 200.41, 201.87; Anal. calcd for $C_{15}H_{18}ClNO_7$ C, 50.08; H, 5.04; N, 3.89; found C, 50.00; H, 5.10; N, 3.84.

Data for compound 5b. Thick viscous liquid, (90%), diastereomeric ratio = 1:1.03

3-(3-Bromo-4,5-dimethoxy-2-nitrophenyl)-3-(1,3-dioxolan-2-yl)-2-methylpropanal (5b).

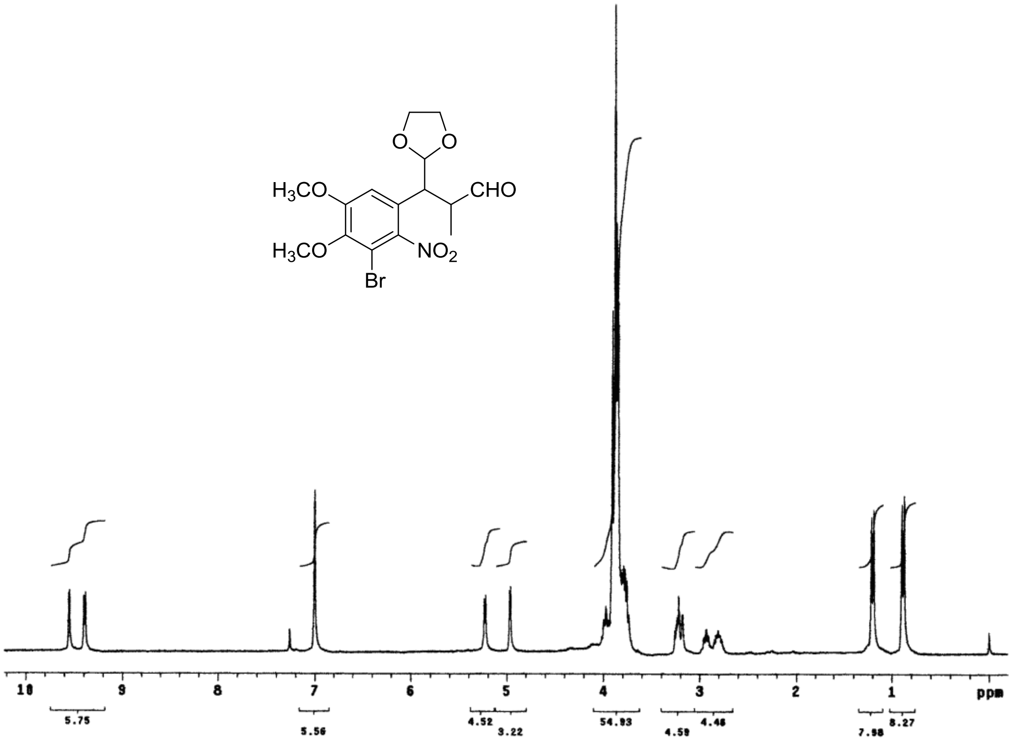

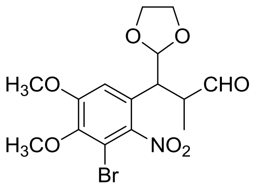

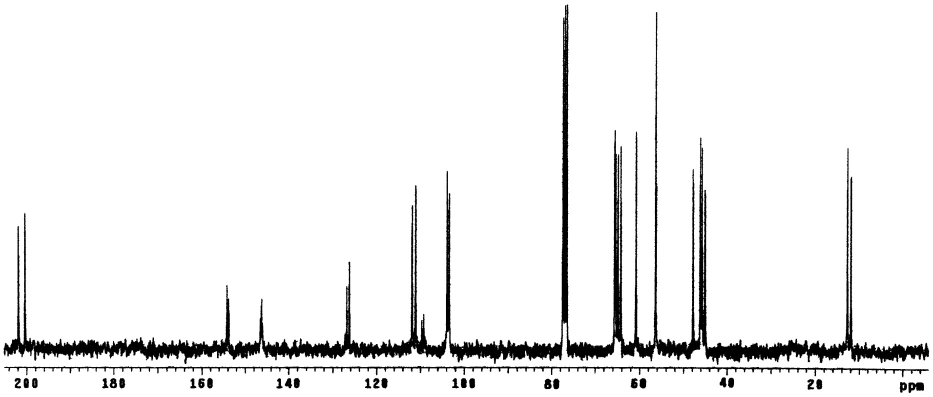

IR ($CHCl_3$) ν/cm^{-1} 2974.0, 2725.2, 1722.3, 1535.2, 1366.5, 1288.3, 1085.8; ^{1}H NMR ($CDCl_3$, 300 MHz, δ/ppm) 0.89 (d, *J* = 7.1 Hz, 3H, **H**$_3$C-CH-), 1.20 (d, *J* = 6.8 Hz, 3H, **H**$_3$C-CH-), 2.77-2.95 (m, 2H, 2 × -C**H**-CH_3), 3.18-3.26 (m, 2H, 2 × -C**H**-Ar), 3.73-4.05 (m, 20H, 4 × **H**$_3$CO-Ar and 2 × -C**H**$_2$-C**H**$_2$-O-), 4.96 (d, *J* = 2.7 Hz, 1H, -O-C**H**-O-), 5.23 (d, *J* = 4.1 Hz, 1H, -O-C**H**-O-), 7.00 (s, 2H, 2 × **H**-Ar), 9.39 (d, *J* = 4.6 Hz, 1H, -C**H**O), 9.55 (d, *J* = 2.2 Hz, 1H, -C**H**O); ^{13}C NMR ($CDCl_3$, 75 MHz, δ/ppm) 11.89, 12.70, 45.05, 45.76, 46.15, 47.83, 56.35, 60.79, 64.23, 64.86, 65.37, 65.62, 103.38, 103.86, 109.18, 111.07, 111.86, 126.26, 126.83, 146.15, 153.80, 154.18, 200.38, 201.88; Anal. calcd for $C_{15}H_{18}BrNO_7$ C, 44.57; H, 4.49; N, 3.47; found C, 44.51; H, 4.55; N, 3.40.

Data for compound 5c. Thick viscous liquid, (91%), diastereomeric ratio = 1:1.52

3-(1,3-Dioxolan-2-yl)-2-methyl-3-(3,4,5-trimethoxy-2-nitrophenyl)propanal (5c).

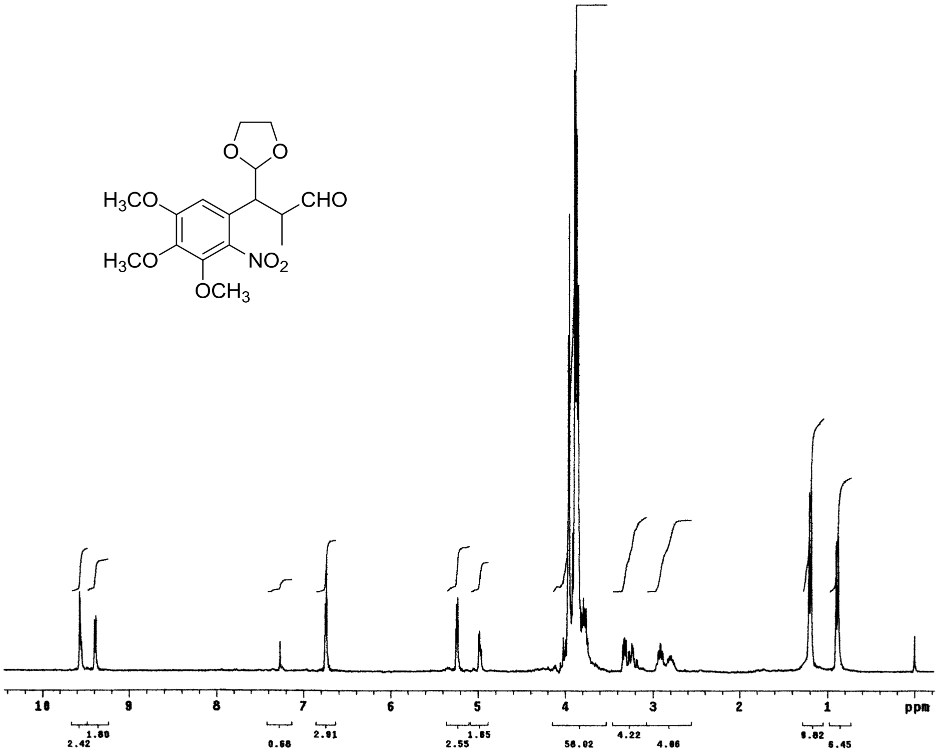

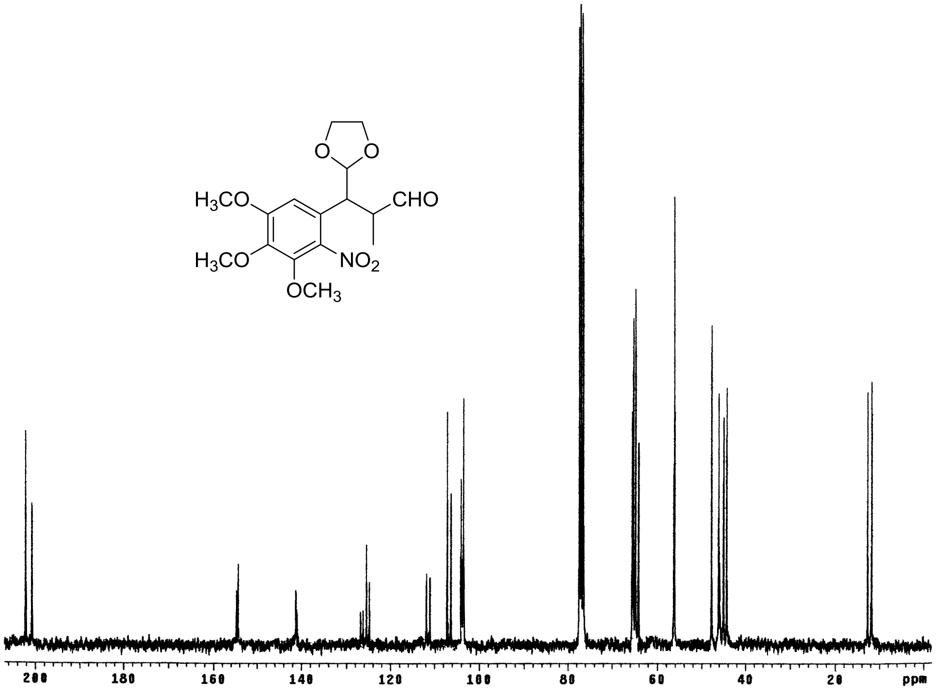

IR ($CHCl_3$) ν/cm^{-1} 2943.4, 2725.5, 1722.4, 1531.5, 1367.5, 1211.3, 1116.8; ^{1}H NMR ($CDCl_3$, 300 MHz, δ/ppm) 0.93 (d, *J* = 6.9 Hz, 3H, **H**$_3$C-CH-), 1.21 (d, *J* = 1.6 Hz, 3H, **H**$_3$C-CH-), 2.75-2.94 (m, 2H, 2 × -C**H**-CH_3), 3.12-3.34 (m, 2H, 2 × -C**H**-Ar), 3.73-4.05 (m, 26H, 6 × **H**$_3$CO-Ar and 2 × -C**H**$_2$-C**H**$_2$-O-), 4.98 (d, *J* = 3.0 Hz, 1H, -O-C**H**-O-), 5.23 (d, *J* = 4.4 Hz, 1H, -O-C**H**-O-), 6.74 (s, 2H, 2 × **H**-Ar), 9.39 (d, *J* = 4.6 Hz, 1H, -C**H**O), 9.56 (d, *J* = 2.2 Hz, 1H, -C**H**O); ^{13}C NMR ($CDCl_3$, 75 MHz, δ/ppm) 11.87, 12.68, 44.32, 45.05, 46.15, 47.49, 56.23, 56.34, 64.16, 64.83, 65.32, 65.57, 103.37, 103.56, 106.33, 107.15, 111.03, 111.83, 124.84, 125.50, 126.25, 126.82, 140.99, 141.26, 154.36, 154.72, 200.81, 202.23; Anal. calcd for $C_{16}H_{21}NO_8$ C, 54.08; H, 5.96; N, 3.94; found C, 54.01; H, 6.05; N, 4.01.

Data for compound 5d. Thick viscous liquid, (91%), diastereomeric ratio = 1:1.41

3-(1,3-Dioxolan-2-yl)-2-methyl-3-(6-nitrobenzo[*d*][1,3]dioxol-5-yl)propanal (5d).

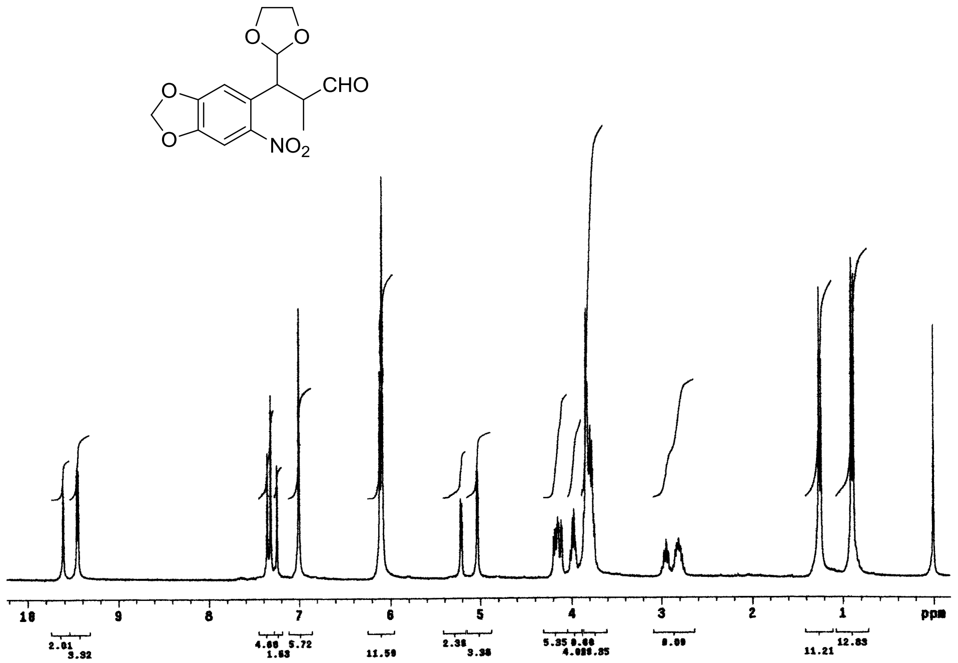

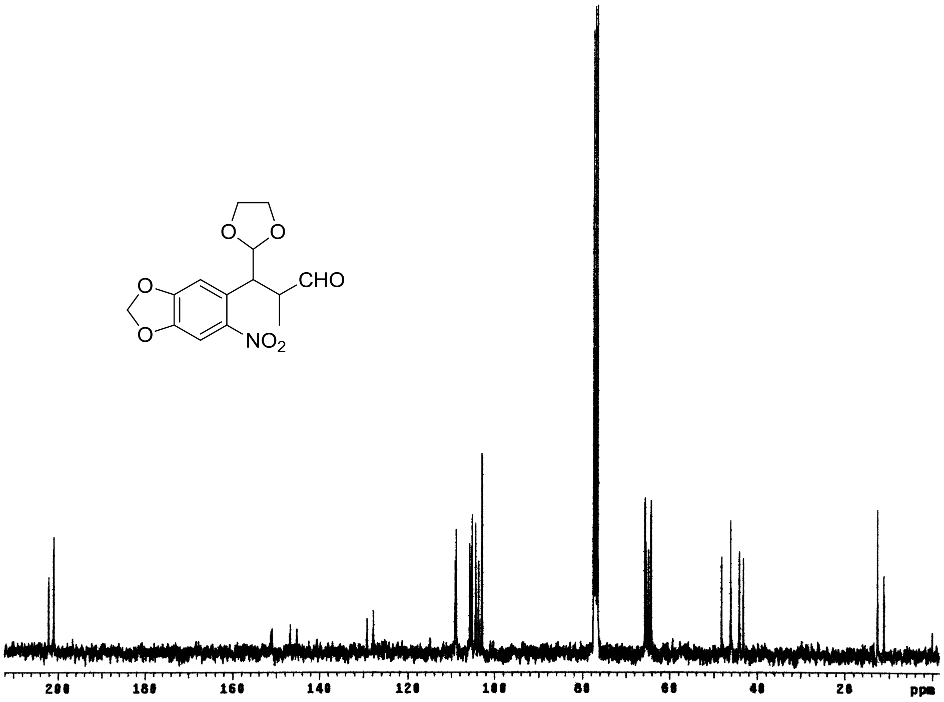

IR ($CHCl_3$) ν/cm^{-1} 2922.2, 2733.2, 1720.5, 1523.8, 1338.6, 1257.6, 1035.8; ^{1}H NMR ($CDCl_3$, 300 MHz, δ/ppm) 0.89 (d, *J* = 7.1 Hz, 3H, **H**$_3$C-CH-), 1.24 (d, *J* = 6.8 Hz, 3H, **H**$_3$C-CH-), 2.77-2.84 (m, 1H, -C**H**-CH_3), 2.92-2.97 (m, 1H, -C**H**-CH_3), 3.74-4.19 (m, 10H, 2 × -C**H**$_2$-C**H**$_2$-O- and 2 × -C**H**-Ar), 5.03 (d, *J* = 2.7 Hz, 1H, -O-C**H**-O-), 5.21 (d, *J* = 4.4 Hz, 1H, -O-C**H**-O-), 6.08 (s, 2H, -O-C**H**$_2$-O-), 6.09 (s, 2H, -O-C**H**$_2$-O-), 7.002 (s, 1H, **H**-Ar), 7.005 (s, 1H, **H**-Ar), 7.32 (s, 1H, **H**-Ar), 7.35 (s, 1H, **H**-Ar), 9.45 (d, *J* = 4.6 Hz, 1H, -C**H**O), 9.61 (d, *J* = 1.6 Hz, 1H, -C**H**O); ^{13}C NMR ($CDCl_3$, 75 MHz, δ/ppm) 11.20, 12.66, 43.24, 44.16, 46.15, 48.23, 64.22, 64.80, 65.38, 65.59, 102.87, 103.66, 104.33, 105.19, 105.67, 108.86, 109.10, 127.81, 129.10, 145.22, 146.75, 150.96, 200.95, 202.08; Anal. calcd for $C_{14}H_{15}NO_7$ C, 54.37; H, 4.89; N, 4.53; found C, 54.30; H, 4.96; N, 4.59.

Data for compound 5e. Thick viscous liquid, (90%), diastereomeric ratio = 1:6

3-(1,3-Dioxolan-2-yl)-2-methyl-3-(2-nitrophenyl)propanal (5e).

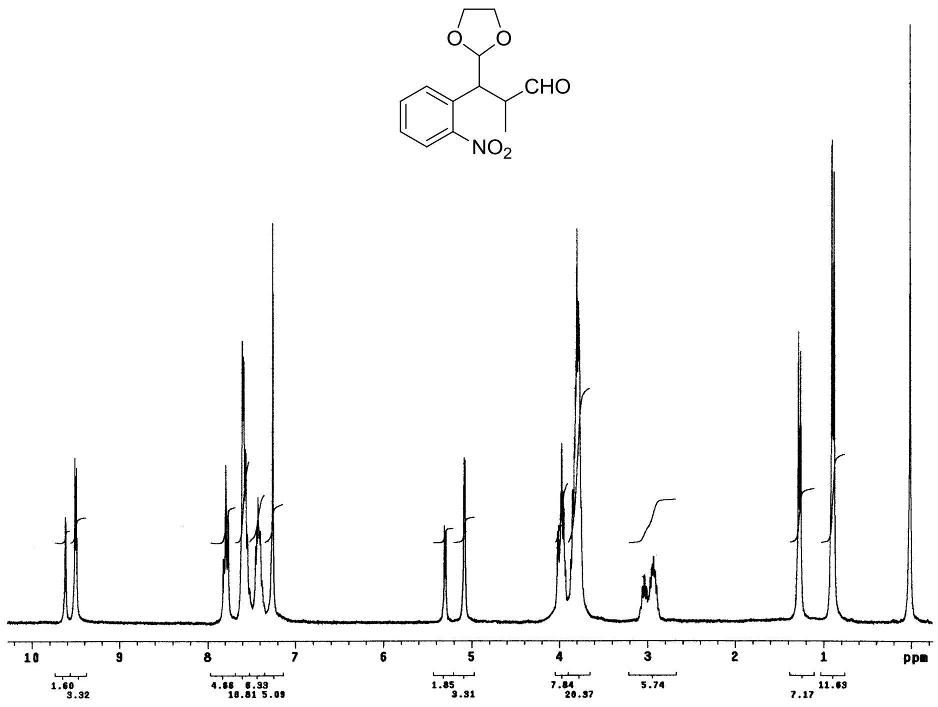

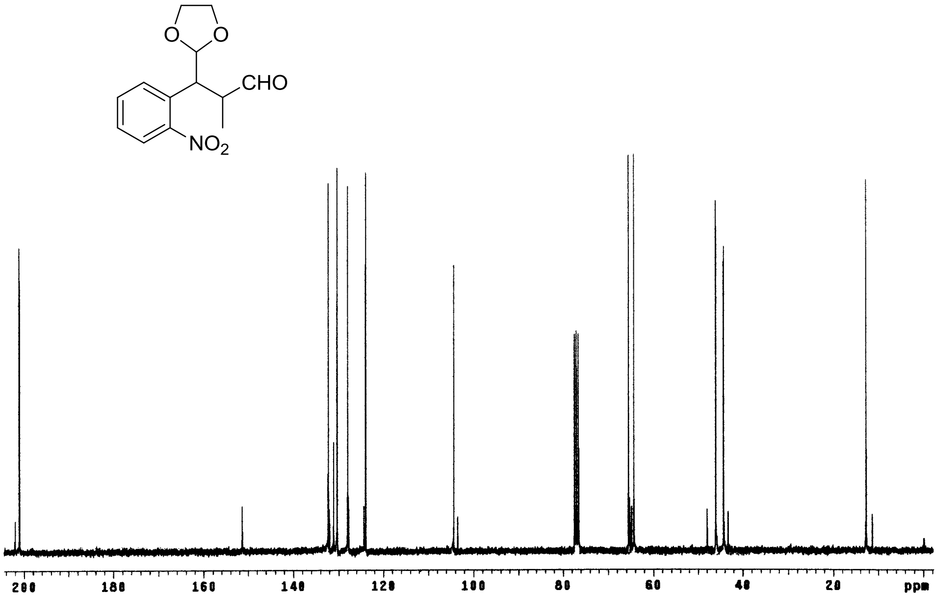

IR ($CHCl_3$) ν/cm^{-1} 2885.3, 2729.0, 1720.4, 1527.5, 1355.9, 1137.9; ^{1}H NMR ($CDCl_3$, 300 MHz, δ/ppm) 0.89 (d, *J* = 6.8 Hz, 3H, **H**$_3$C-CH-), 1.27 (d, *J* = 7.1 Hz, 3H, **H**$_3$C-CH-), 2.89-2.97 (m, 1H, -C**H**-CH_3), 3.01-3.05 (m, 1H, -C**H**-CH_3), 3.74-3.86 (m, 8H, 2 × -C**H**$_2$-C**H**$_2$-O-), 3.92-4.01 (m, 2H, 2 × -C**H**-Ar), 5.08 (d, *J* = 3.0 Hz, 1H, -O-C**H**-O-), 5.29 (d, *J* = 4.4 Hz, 1H, -O-C**H**-O-), 7.37-7.45 (m, 2H, 2 × **H**-Ar), 7.51-7.60 (m, 4H, 4 × **H**-Ar), 7.76-7.81 (m, 2H, 2 × **H**-Ar), 9.50 (d, *J* = 4.6 Hz, 1H, -C**H**O), 9.62 (d, *J* = 1.6 Hz, 1H, -C**H**O); ^{13}C NMR ($CDCl_3$, 75 MHz, δ/ppm) 11.30, 12.71, 43.24, 44.28, 46.04, 47.95, 64.33, 64.82, 65.28, 65.49, 103.49, 104.29, 123.97, 124.35, 127.72, 127.95, 130.32, 131.11, 132.01, 132.27, 151.38, 201.04, 201.97; Anal. calcd for $C_{13}H_{15}NO_5$ C, 58.86; H, 5.70; N, 5.28; found C, 58.79; H, 5.76; N, 5.31.

Data for compound 5f. Thick viscous liquid, (91%), diastereomeric ratio = 1:6.58

3-(2-Bromo-3,4,5-trimethoxy-6-nitrophenyl)-3-(1,3-dioxolan-2-yl)-2-methylpropanal (5f).

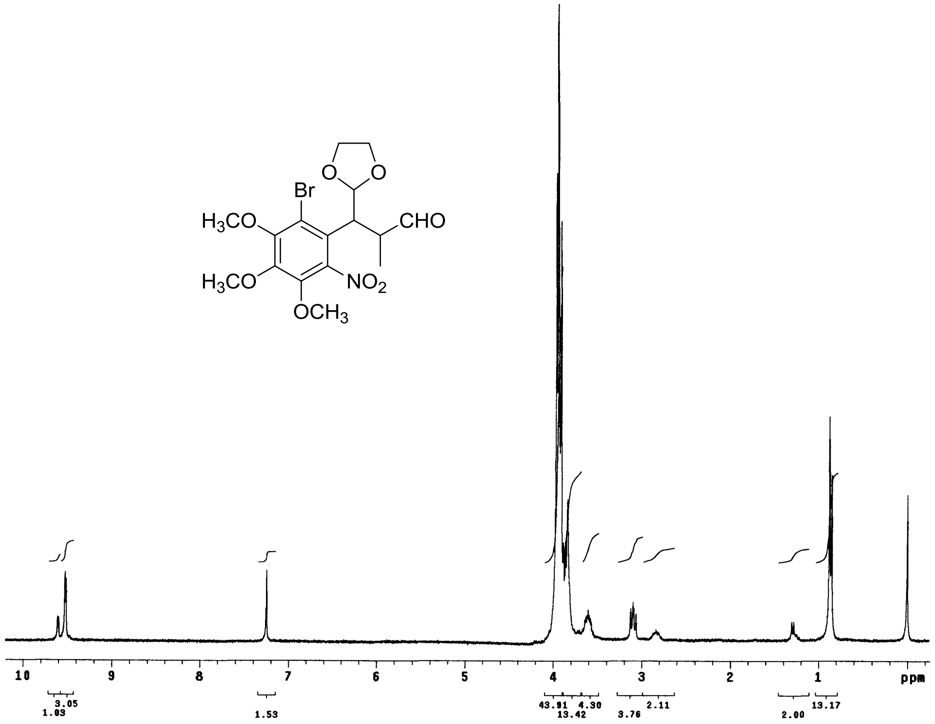

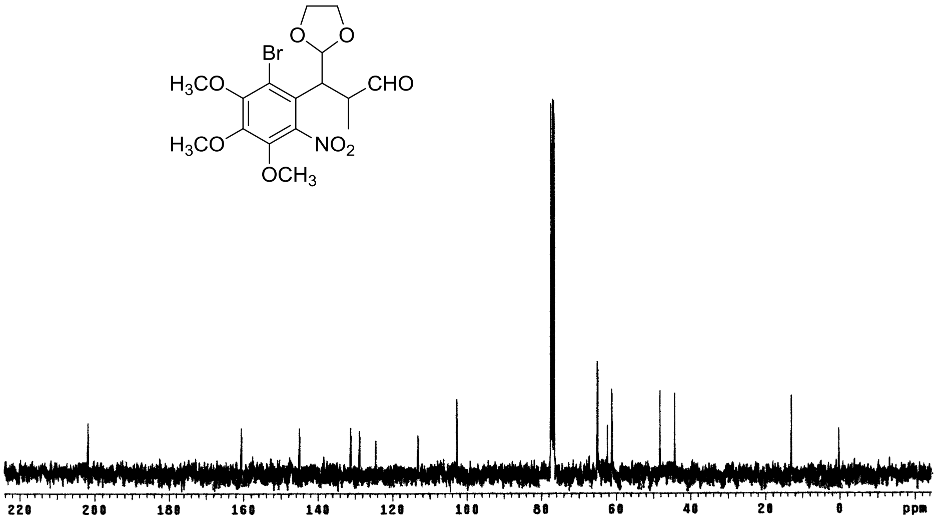

IR ($CHCl_3$) ν/cm^{-1} 2943.4, 2721.6, 1726.3, 1539.2, 1365.6, 1230.6, 1024.2; ^{1}H NMR ($CDCl_3$, 300 MHz, δ/ppm) 0.86 (d, *J* = 7.4 Hz, 3H, **H_3**C-CH-), 1.28 (d, *J* = 6.8 Hz, 3H, **H_3**C-CH-), 2.83-3.06 (m, 1H, -C**H**-CH_3), 3.08-3.12 (m, 1H, -C**H**-CH_3), 3.58-3.63 (m, 2H, 2 × -C**H**-Ar), 3.83-3.96 (m, 28H, 6 × **H_3**CO-Ar, 2 × -C**H_2**-C**H_2**-O- and 2 × -O-C**H**-O-), 9.51 (d, *J* = 3.5 Hz, 1H, -C**H**O), 9.59 (d, *J* = 3.3 Hz, 1H, -C**H**O); ^{13}C NMR ($CDCl_3$, 75 MHz, δ/ppm) 13.04, 44.29, 48.25, 61.22, 62.37, 64.96, 65.11, 102.76, 113.20, 124.50, 128.16, 130.12, 144.79, 160.58, 201.75; Anal. calcd for $C_{16}H_{20}BrNO_8$ C, 44.26; H, 4.64; N, 3.23; found C, 44.17; H, 4.66; N, 3.29.

Data for compound 5g. Thick viscous liquid, (90%), diastereomeric ratio = 1:1.5

3-(1,3-Dioxolan-2-yl)-3-(5-methoxy-2-nitrophenyl)-2-methylpropanal (5g).

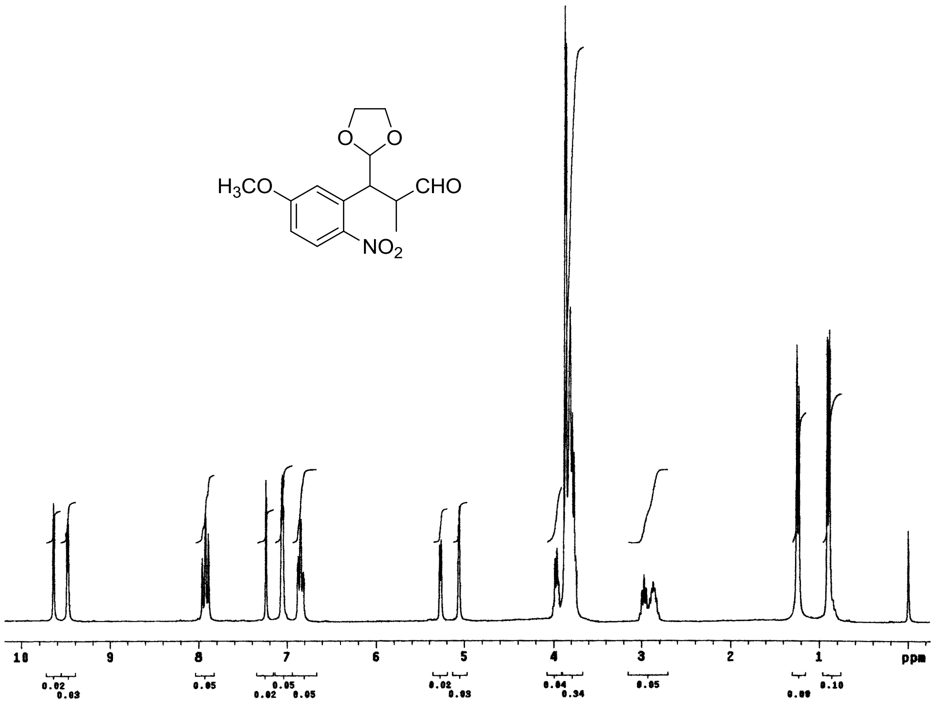

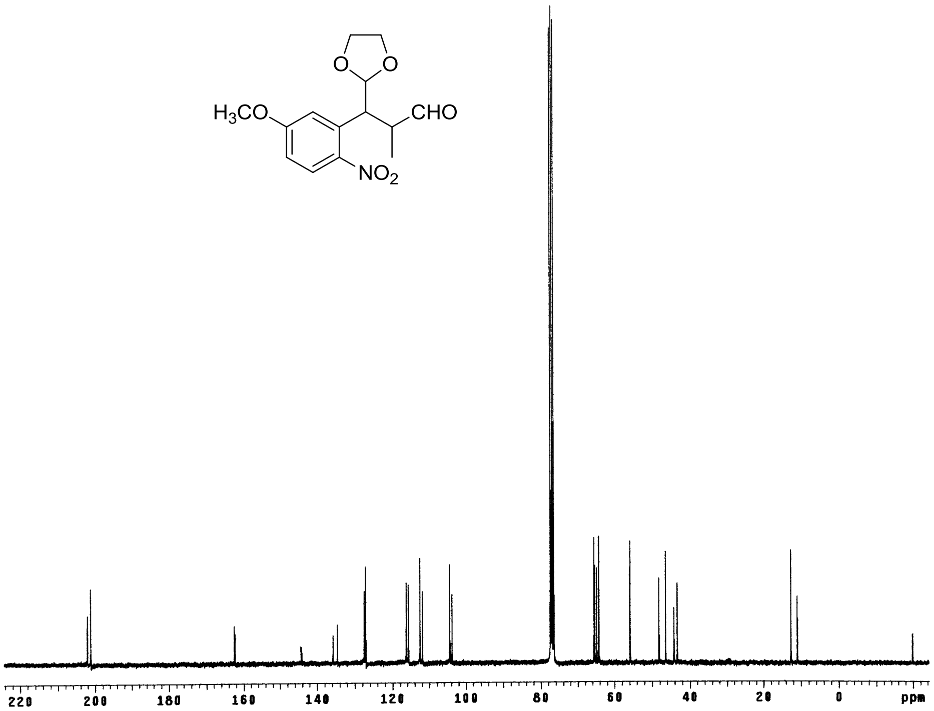

IR ($CHCl_3$) ν/cm^{-1} 2968.5, 2729.3, 1720.5, 1516.1, 1346.3, 1232.5, 1031.9; ^{1}H NMR ($CDCl_3$, 300 MHz, δ/ppm) 0.90 (d, *J* = 7.1 Hz, 3H, **H**$_3$C-CH-), 1.23 (d, *J* = 7.1 Hz, 3H, **H**$_3$C-CH-), 2.83-2.88 (m, 1H, -C**H**-CH_3), 2.90-2.99 (m, 1H, -C**H**-CH_3), 3.74-3.87 (m, 14H, 2 × **H**$_3$CO-Ar and 2 × -C**H**$_2$-C**H**$_2$-O-), 3.94-3.98 (m, 2H, 2 × -C**H**-Ar), 5.06 (d, *J* = 1.6 Hz, 1H, -O-C**H**-O-), 5.27 (d, *J* = 4.9 Hz, 1H, -O-C**H**-O-), 6.81-6.87 (m, 2H, 2 × **H**-Ar), 7.05 (d, *J* = 1.6 Hz, 2H, 2 × **H**-Ar), 7.89-7.96 (m, 2H, 2 × **H**-Ar), 9.48 (d, *J* = 4.6 Hz, 1H, -C**H**O), 9.84 (s, 1H, -C**H**O); ^{13}C NMR ($CDCl_3$, 75 MHz, δ/ppm) 10.99, 12.70, 43.35, 44.22, 46.44, 48.15, 55.84, 55.86, 64.29, 64.86, 65.40, 65.60, 103.77, 104.41, 111.86, 112.57, 115.73, 116.29, 127.12, 127.51, 134.62, 135.78, 144.18, 162.28, 162.51, 201.18, 202.11; Anal. calcd for $C_{14}H_{17}NO_6$ C, 56.94; H, 5.80; N, 4.74; found C, 56.89; H, 5.84; N, 4.79.

Data for compound 5h. Thick viscous liquid, (91%), diastereomeric ratio: 1:1.44

3-(2-Chloro-6-nitrophenyl)-3-(1,3-dioxolan-2-yl)-2-methylpropanal (5h).

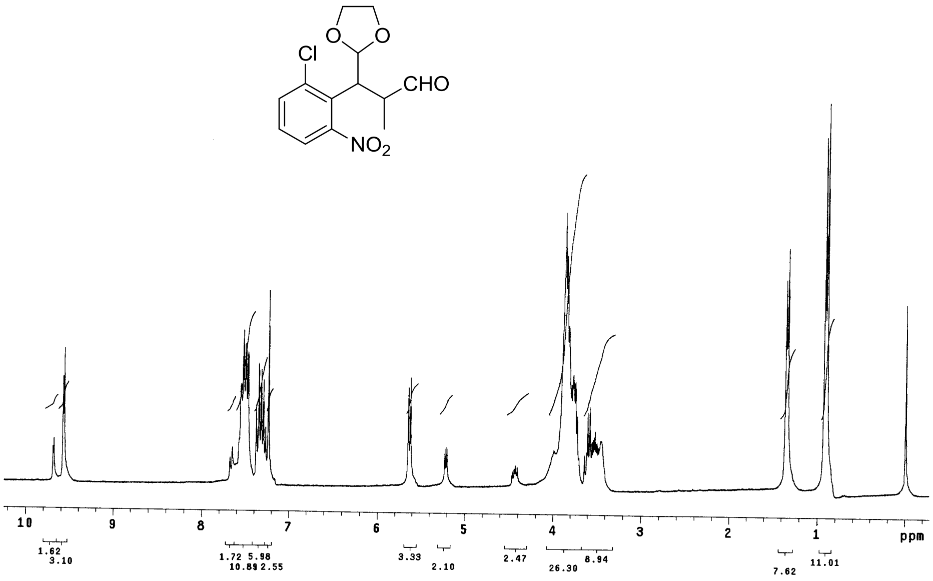

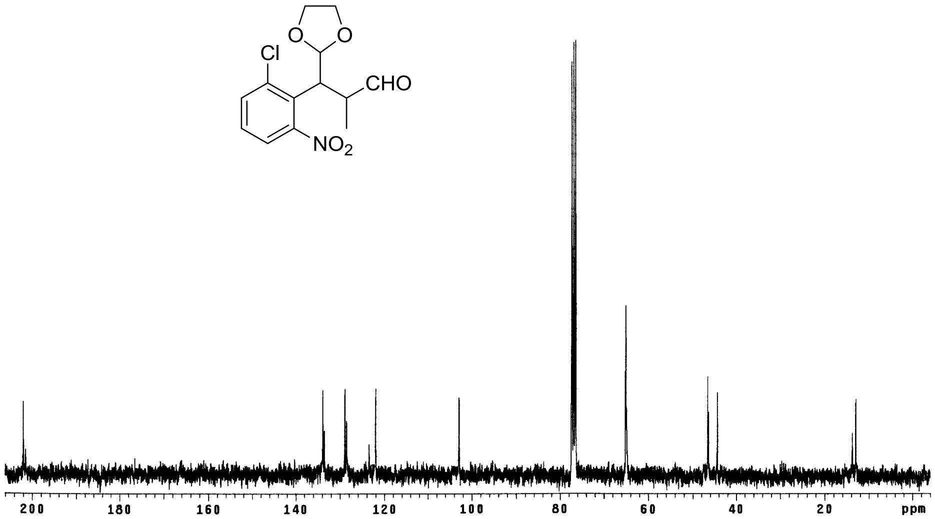

IR ($CHCl_3$) ν/cm^{-1} 2972.4, 2723.5, 1724.4, 1533.4, 1363.7, 1114.8; ^{1}H NMR ($CDCl_3$, 300 MHz, δ/ppm) 0.92 (d, *J* = 6.8 Hz, 3H, **H**$_3$C-CH-), 1.35 (d, *J* = 6.6 Hz, 3H, **H**$_3$C-CH-), 3.45-3.64 (m, 2H, 2 × -C**H**-CH$_3$), 3.70-3.99 (m, 10H, 2 × -C**H**$_2$-C**H**$_2$-O- and 2 × -C**H**-Ar), 4.41-4.46 (m, 2H, 2 × -C**H**-Ar), 5.21 (d, *J* = 6.3 Hz, 1H, -O-C**H**-O-), 5.64 (d, *J* = 7.1 Hz, 1H, -O-C**H**-O-), 7.27-7.38 (m, 2H, 2 × **H**-Ar), 7.48-7.68 (m, 4H, 4 × **H**-Ar), 9.58 (d, *J* = 3.5 Hz, 1H, -C**H**O), 9.69 (d, *J* = 2.7 Hz, 1H, -C**H**O); ^{13}C NMR ($CDCl_3$, 75 MHz, δ/ppm) 13.04, 13.83, 44.39, 46.41, 46.63, 65.13, 102.93, 122.07, 123.53, 128.61, 128.97, 133.58, 133.91, 201.87, 202.04; Anal. calcd for $C_{13}H_{14}ClNO_5$ C, 52.10; H, 4.71; N, 4.67; found C, 52.01; H, 4.78; N, 4.72.

Data for compound 6a. Viscous liquid, (85%)

8-Chloro-6,7-dimethoxy-3-methylquinoline-4-carbaldehyde (6a).

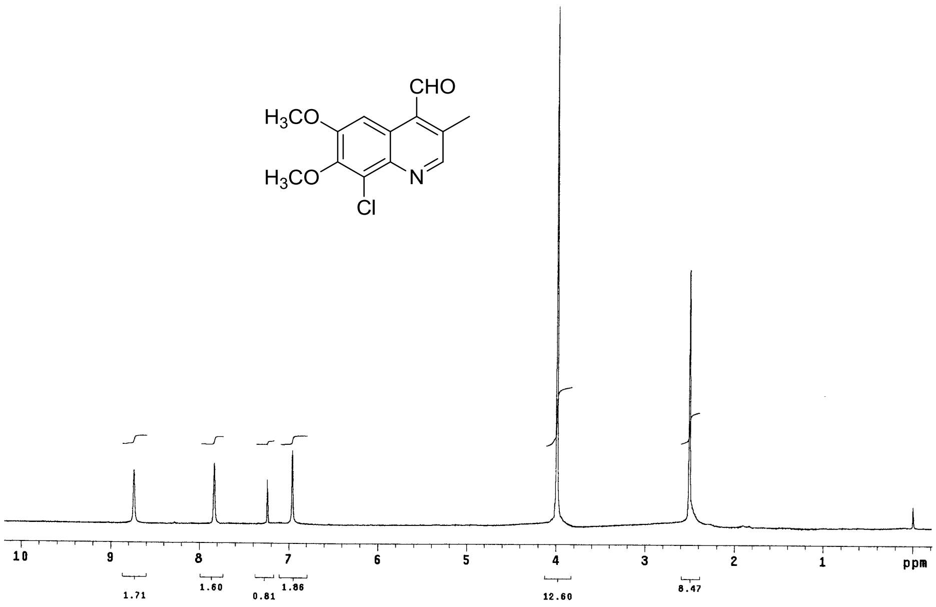

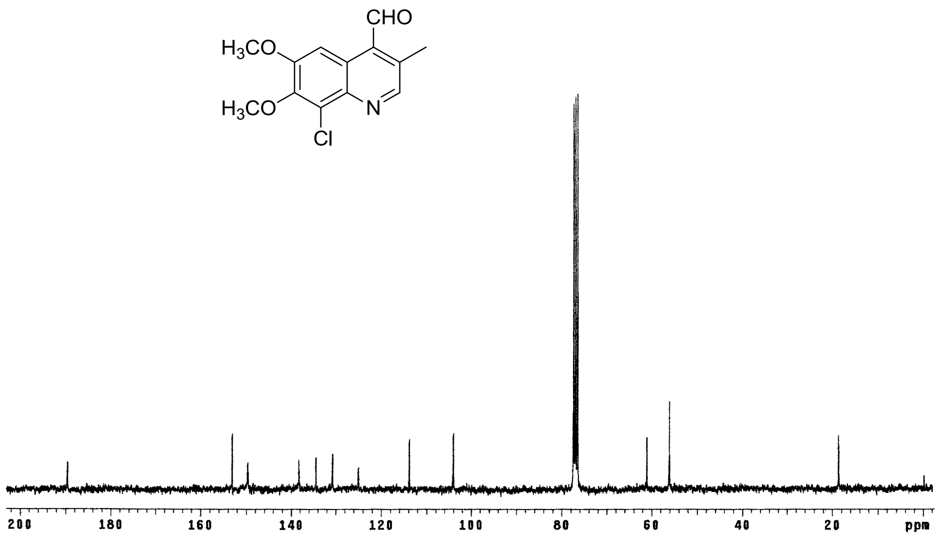

IR ($CHCl_3$) ν/cm^{-1} 2926.1, 1720.5, 1610.6, 1265.3; ^{1}H NMR ($CDCl_3$, 300 MHz, δ/ppm) 2.50 (s, 3H, **H**$_3$C-Ar), 3.99 (s, 3H, **H**$_3$CO-Ar), 4.00 (s, 3H, **H**$_3$CO-Ar), 6.96 (s, 1H, **H**-Ar), 7.83 (s, 1H, **H**-Ar), 8.74 (s, 1H, -C**H**O); ^{13}C NMR ($CDCl_3$, 75 MHz, δ/ppm) 18.63, 56.10, 61.07, 104.03, 114.06, 125.01, 130.76, 134.71, 138.28, 149.68, 153.11, 190.09; Anal. calcd for $C_{13}H_{12}ClNO_3$ C, 58.77; H, 4.55; N, 5.27; found C, 58.68; H, 4.64; N, 5.36.

Data for compound 6b. Viscous liquid, (84%)

8-Bromo-6,7-dimethoxy-3-methylquinoline-4-carbaldehyde (6b).

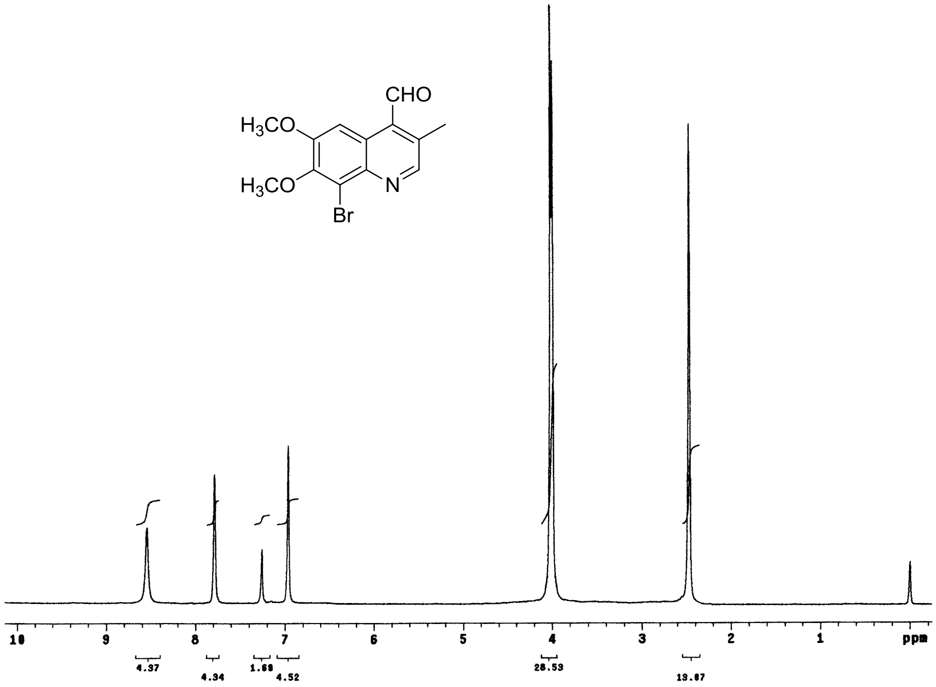

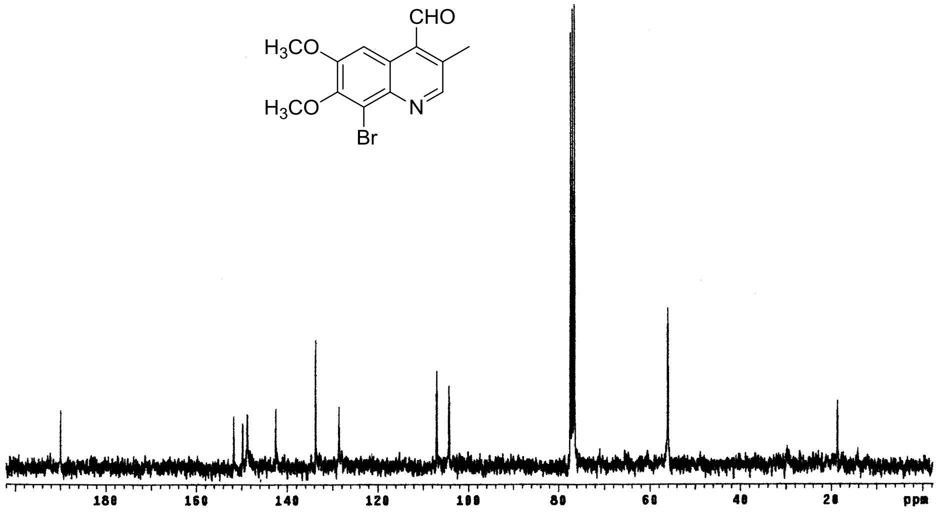

IR ($CHCl_3$) ν/cm^{-1} 2929.9, 2746.7, 1720.5, 1240.2; ^{1}H NMR ($CDCl_3$, 300 MHz, δ/ppm) 2.47 (s, 3H, **H**$_3$C-Ar), 3.99 (s, 3H, **H**$_3$CO-Ar), 4.01 (s, 3H, **H**$_3$CO-Ar), 6.96 (s, 1H, **H**-Ar), 7.78 (s, 1H, **H**-Ar), 8.54 (s, 1H, -C**H**O); ^{13}C NMR ($CDCl_3$, 75 MHz, δ/ppm) 18.23, 56.00, 56.10, 104.33, 106.99, 128.59, 133.80, 142.52, 148.75, 149.76, 151.73, 190.31; Anal. calcd for $C_{13}H_{12}BrNO_3$ C, 50.34; H, 3.90; N, 4.52; found C, 50.41; H, 3.95; N, 4.47.

Data for compound 6c. Viscous liquid, (85%)

6,7,8-Trimethoxy-3-methylquinoline-4-carbaldehyde (6c).

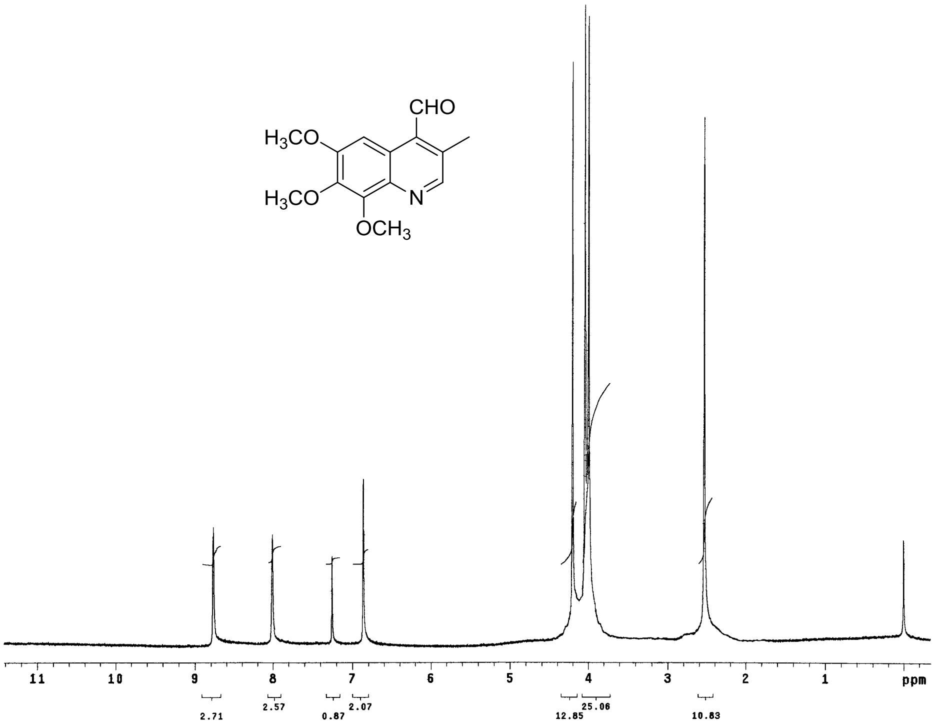

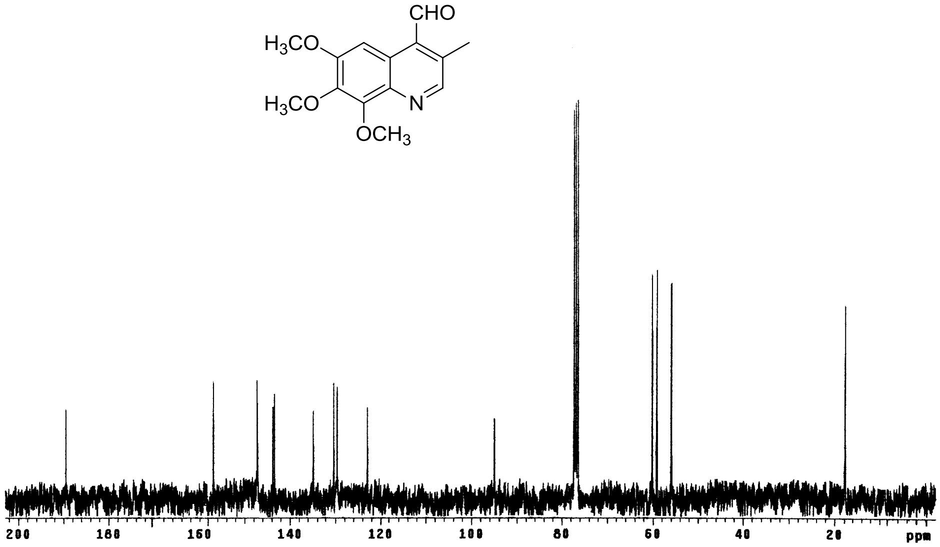

IR ($CHCl_3$) ν/cm^{-1} 3003.2, 1712.8, 1610.6, 1222.9; ^{1}H NMR ($CDCl_3$, 300 MHz, δ/ppm) 2.53 (s, 3H, **H**$_3$C-Ar), 3.99 (s, 3H, **H**$_3$CO-Ar), 4.04 (s, 3H, **H**$_3$CO-Ar), 4.20 (s, 3H, **H**$_3$CO-Ar), 6.86 (s, 1H, **H-**Ar), 8.01 (s, 1H, **H**-Ar), 8.77 (s, 1H, **-**C**H**O); ^{13}C NMR ($CDCl_3$, 75 MHz, δ/ppm) 18.07, 56.03, 59.21, 60.09, 94.92, 123.03, 129.73, 130.58, 135.03, 143.59, 143.89, 147.29, 158.94, 189.77; Anal. calcd for $C_{14}H_{15}NO_4$ C, 64.36; H, 5.79; N, 5.36; found C, 64.31; H, 5.78; N, 5.38.

Data for compound 6d. Viscous liquid, (83%)

7-Methyl-[1,3]dioxolo[4,5-*g*]quinoline-8-carbaldehyde (6d).

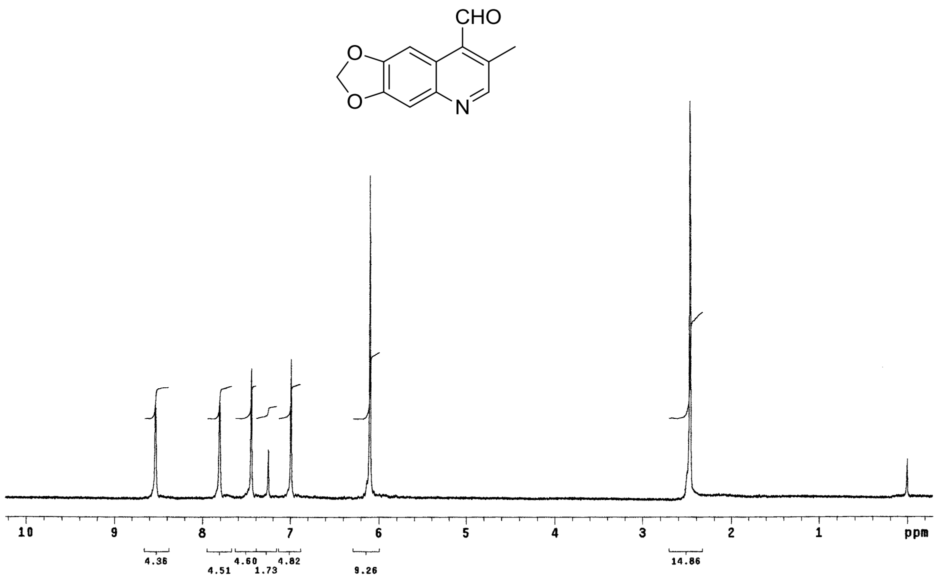

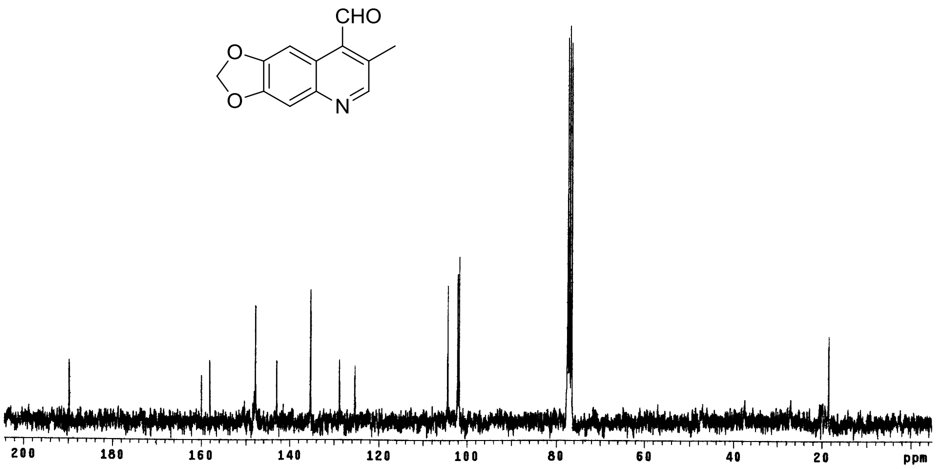

IR ($CHCl_3$) ν/cm^{-1} 2956.9, 1734.0, 1616.4, 1464.0, 1238.3, 1039.6; ^{1}H NMR ($CDCl_3$, 300 MHz, δ/ppm) 2.46 (s, 3H, $\mathbf{H_3}C$-Ar), 6.09 (s, 2H, -O-C$\mathbf{H_2}$-O-), 6.99 (s, 1H, **H**-Ar), 7.45 (s, 1H, **H**-Ar), 7.80 (s, 1H, **H**-Ar), 8.53 (s, 1H, -C**H**O); ^{13}C NMR ($CDCl_3$, 75 MHz, δ/ppm) 18.49, 101.80, 102.12, 104.38, 125.32, 128.82, 135.30, 143.01, 147.92, 158.03, 160.01, 190.02; Anal. calcd for $C_{12}H_9NO_3$ C, 66.97; H, 4.22; N, 6.51; found C, 66.91; H, 4.28; N, 6.59.

Data for compound 6e. White solid, mp 54 °C, (80%)

3-Methylquinoline-4-carbaldehyde (6e).

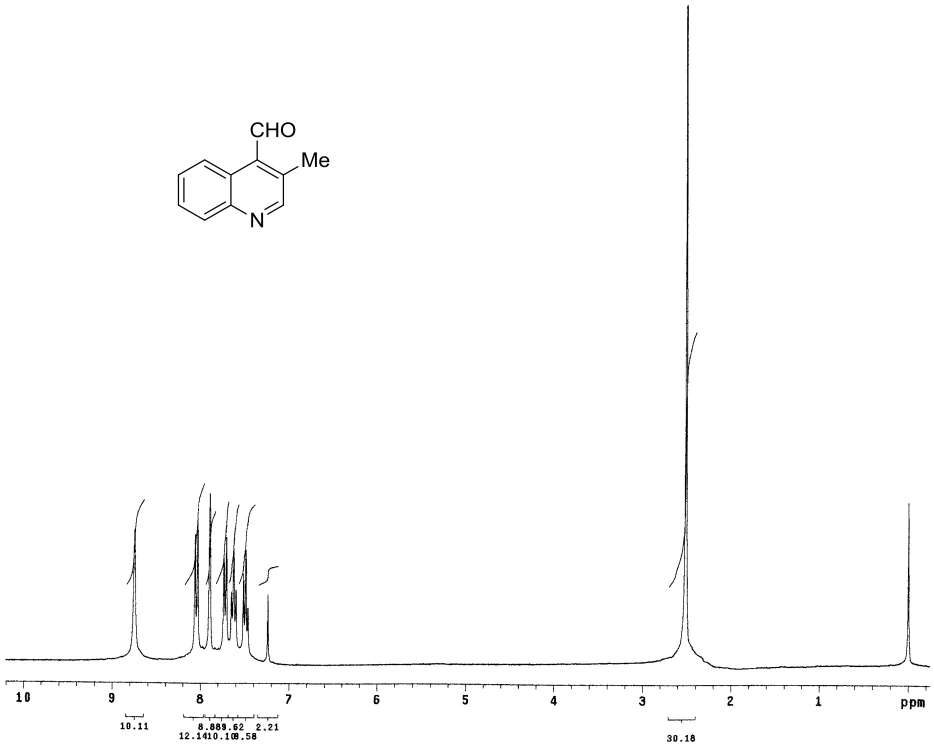

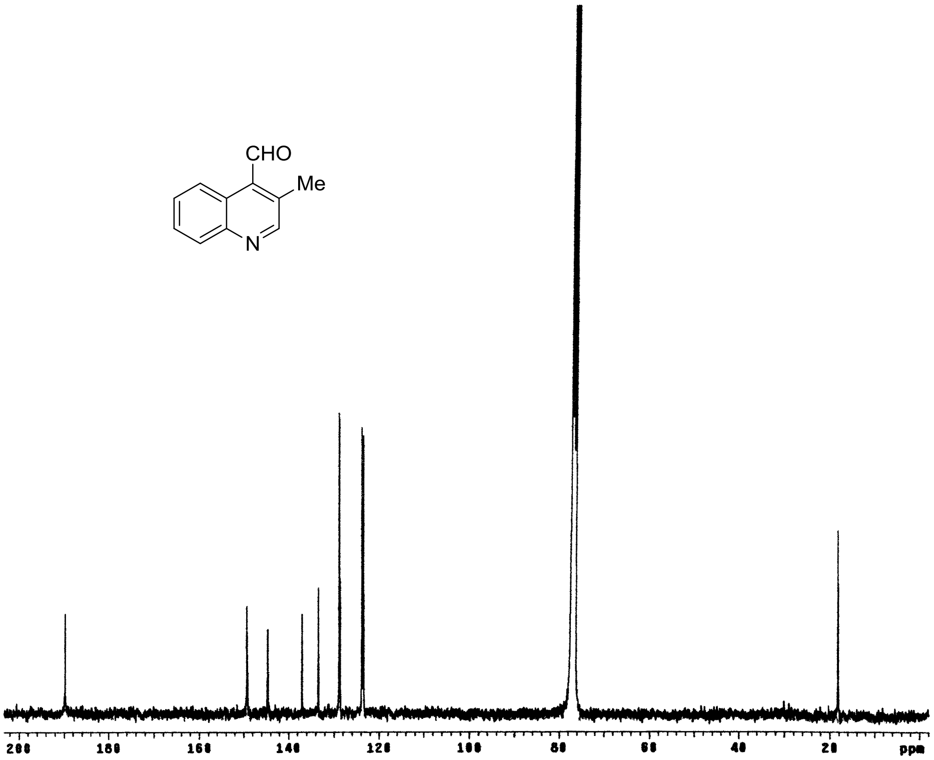

IR ($CHCl_3$) ν/cm^{-1} 2924.1, 1712.8, 1498.7; ^{1}H NMR ($CDCl_3$, 300 MHz, δ/ppm) 2.51 (s, 3H, **H**$_3$C-Ar), 7.49 (t, *J* = 7.1 Hz, 1H, **H**-Ar), 7.62 (t, *J* = 6.8 Hz, 1H, **H**-Ar), 7.72 (d, *J* = 7.9 Hz, 1H, **H**-Ar), 7.89 (s, 1H, **H**-Ar), 8.04 (d, *J* = 8.5 Hz, 1H, **H**-Ar), 8.75 (s, 1H, -C**H**O); ^{13}C NMR ($CDCl_3$, 75 MHz, δ/ppm) 18.03, 123.52, 123.81, 129.11, 133.79, 137.12, 144.85, 149.67, 189.93; Anal. calcd for $C_{11}H_9NO$ C, 77.17; H, 5.30; N, 8.18; found C, 77.21; H, 5.24; N, 8.27.

Data for compound 6f. Viscous liquid, (79%)

5-Bromo-6,7,8-trimethoxy-3-methylquinoline-4-carbaldehyde (6f).

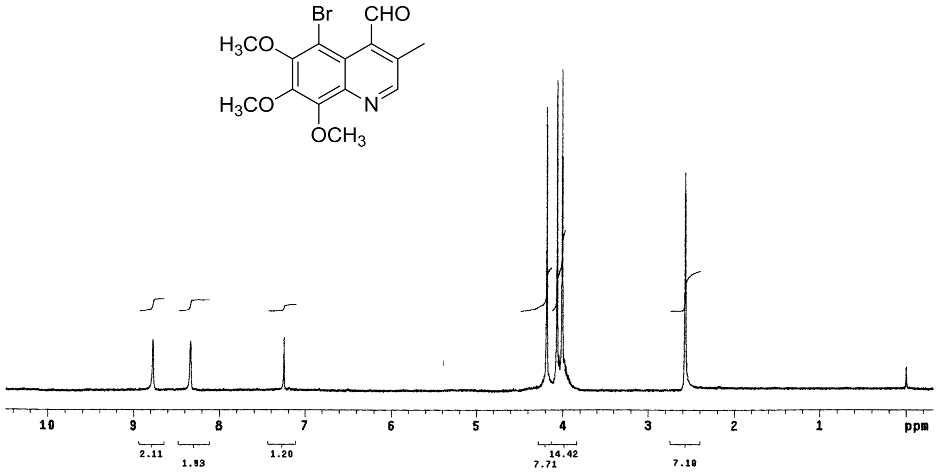

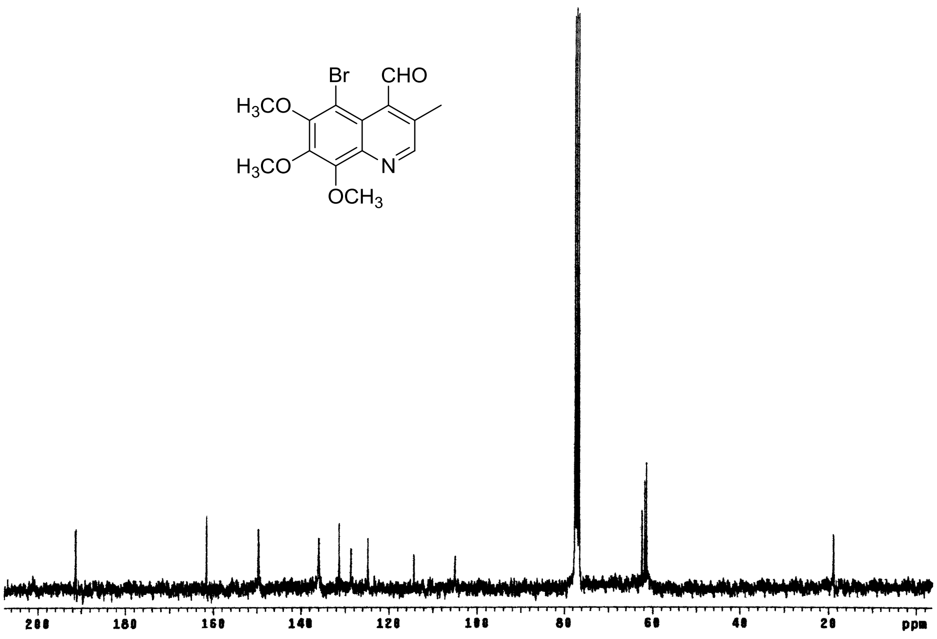

IR ($CHCl_3$) ν/cm^{-1} 2928.0, 1726.3, 1593.2, 1261.4; ^{1}H NMR ($CDCl_3$, 300 MHz, δ/ppm) 2.57 (s, 3H, **H**$_3$C-Ar), 4.00 (s, 3H, **H**$_3$CO-Ar), 4.06 (s, 3H, **H**$_3$CO-Ar), 4.18 (s, 3H, **H**$_3$CO-Ar), 8.33 (s, 1H, **H**-Ar), 8.77 (s, 1H, -C**H**O); ^{13}C NMR ($CDCl_3$, 75 MHz, δ/ppm) 18.84, 61.36, 61.74, 62.37, 105.02, 108.20, 124.80, 128.70, 131.35, 135.94, 149.59, 162.03, 191.05; Anal. calcd for $C_{14}H_{14}BrNO_4$ C, 49.43; H, 4.15; N, 4.12; found C, 49.49; H, 4.21; N, 4.08.

Data for compound 6g. Viscous liquid, (80%)

6-Methoxy-3-methylquinoline-4-carbaldehyde (6g).

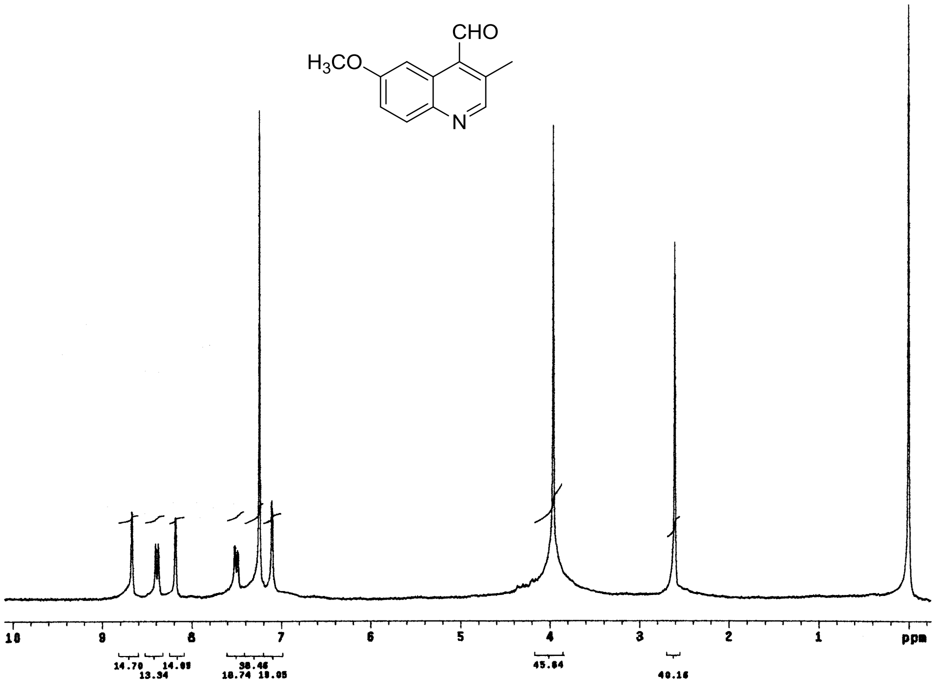

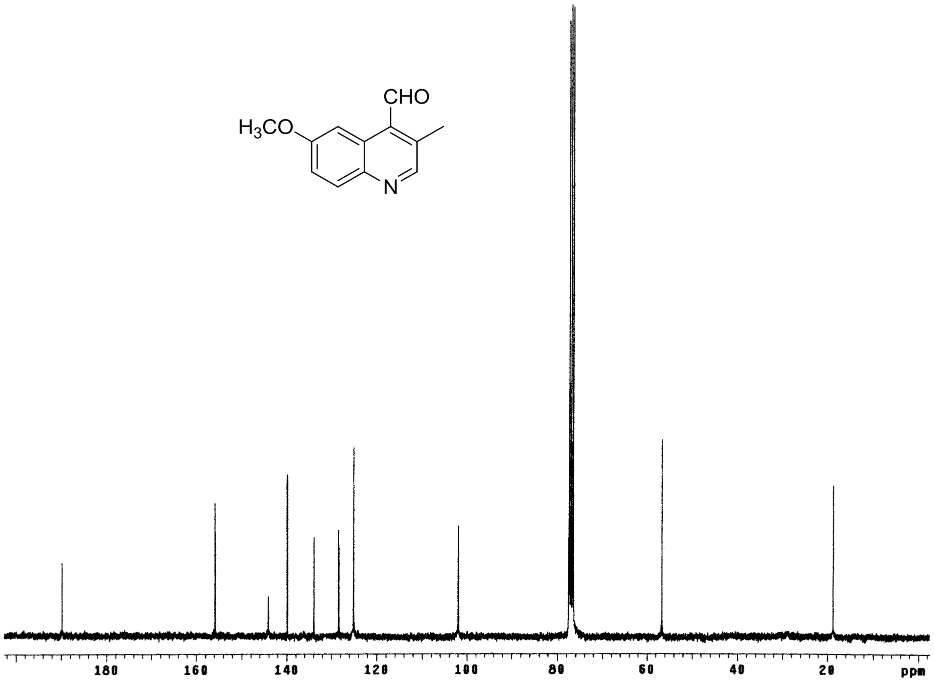

IR ($CHCl_3$) ν/cm^{-1} 2985.9, 2686.9, 1718.6, 1620.2, 1265.3; ^{1}H NMR ($CDCl_3$, 300 MHz, δ/ppm) 2.60 (s, 3H, $\mathbf{H}_3$C-Ar), 3.85 (s, 3H, $\mathbf{H}_3$CO-Ar), 7.10 (s, 1H, **H**-Ar), 7.46 (d, *J* = 9.3 Hz, 1H, **H**-Ar), 8.18 (s, 1H, **H**-Ar), 8.39 (d, *J* = 9.0 Hz, 1H, **H**-Ar), 8.67 (s, 1H, -C**H**O); ^{13}C NMR ($CDCl_3$, 75 MHz, δ/ppm) 18.58, 56.32, 102.00, 125.08, 128.24, 134.02, 140.09, 144.04, 155.98, 189.97; Anal. calcd for $C_{12}H_{11}NO_2$ C, 71.63; H, 5.51; N, 6.96; found C, 71.69; H, 5.59; N, 6.91.

Data for compound 6h. Viscous liquid, (82%)

5-Chloro-3-methylquinoline-4-carbaldehyde (6h).

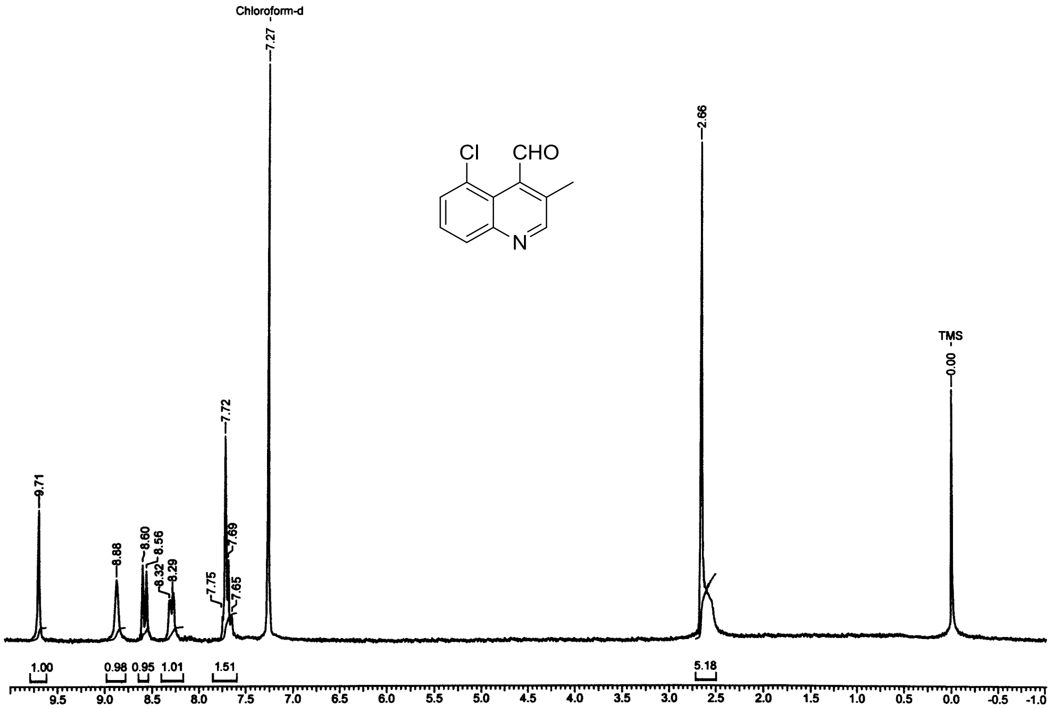

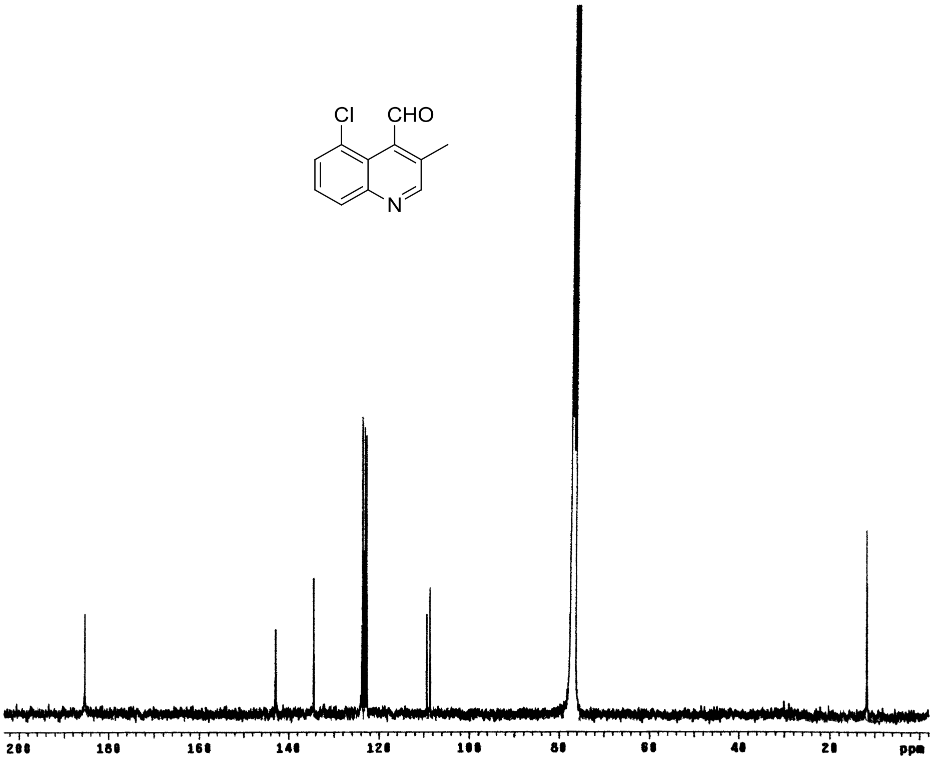

IR ($CHCl_3$) ν/cm^{-1} 3078.4, 1710.9, 1587.4; ^{1}H NMR ($CDCl_3$, 300 MHz, δ/ppm) 2.66 (s, 3H, **H**$_3$C-Ar), 7.65-7.75 (m, 1H, **H**-Ar), 8.31 (d, *J* = 9.1 Hz, 1H, **H**-Ar), 8.58 (d, *J* = 8.8 Hz, 1H, **H**-Ar), 8.88 (s, 1H, **H**-Ar), 9.71 (s, 1H, -C**H**O); ^{13}C NMR ($CDCl_3$, 75 MHz, δ/ppm) 11.86, 108.23, 109.17, 122.74, 122.92, 123.88, 134.12, 142.49, 184.71; Anal. calcd for $C_{11}H_8ClNO$ C, 64.25; H, 3.92; N, 6.81; found C, 64.19; H, 3.98; N, 6.80.
